# Synthesis of γ‑Lactams by Intermolecular (3 + 2) Annulation of Siloxy Alkynes and 3‑Aminooxetanes

**DOI:** 10.1021/prechem.5c00029

**Published:** 2025-04-11

**Authors:** Xiang Li, Qiang Feng, Shuxuan Liu, Hai Huang, Zhengyu Han, Jianwei Sun

**Affiliations:** † Jiangsu Key Laboratory of Advanced Catalytic Materials & Technology, School of Petrochemical Engineering, 554686 Changzhou University, Changzhou, Jiangsu 213164, China; ‡ Department of Chemistry and the Hong Kong Branch of Chinese National Engineering Research Centre for Tissue Restoration & Reconstruction, 58207 The Hong Kong University of Science and Technology, Clear Water Bay, Kowloon 999077, Hong Kong SAR, China

**Keywords:** Siloxy alkyne, 3-Aminooxetane, (3 + 2) Annulation, γ-Lactam, Annulation

## Abstract

A silver-catalyzed intermolecular (3 + 2) annulation of siloxy alkynes and 3-aminooxetanes has been developed. This process provides mild and convenient access to useful γ-butyrolactams with high regio- and stereoselectivity. Mechanistically, intermolecular C–N bond formation likely precedes oxetane ring opening.

## Graphical abstract

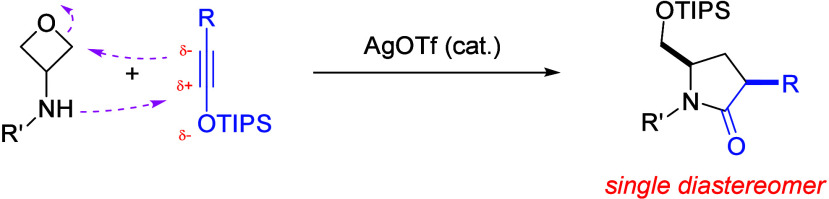

Siloxy alkynes represent a versatile family of synthetic building blocks.^1^ Their electron-rich triple bond is highly polarized and thus can serve as extraordinary C2 partner for annulation with a range of dipolar molecules.^1–3^ More importantly, such an annulation typically generates a silyl enol ether functionality, which can be further utilized for diverse transformations and thus permit the assembly of densely functionalized cyclic carbonyl compounds. In the past, a wide range of dipolar molecules, including those uncharged amphoteric molecules bearing both nucleophilic and electrophilic sites with proper distance, have been widely utilized for the synthesis of cyclic molecules of different ring sizes with siloxy alkynes.^1–3^ However, it is worth noting that the majority of these reaction partners contain highly electrophilic groups, such as ketenes, ketones, aldehydes, oxoniums, and imines.^1–3^ Such reactive electrophiles easily initiate the annulation by forming the carbon–electrophile bond via nucleophilic attack from the electron-rich siloxy alkyne, forming a ketenium species that triggers the ring closure with nucleophile motif (Scheme 1A). However, when the electrophile motif is not sufficiently reactive, we envision that annulation may reverse the bond forming order, thus providing opportunities for the discovery of new reactivities of this type of intriguing molecules.

**Scheme 1:**
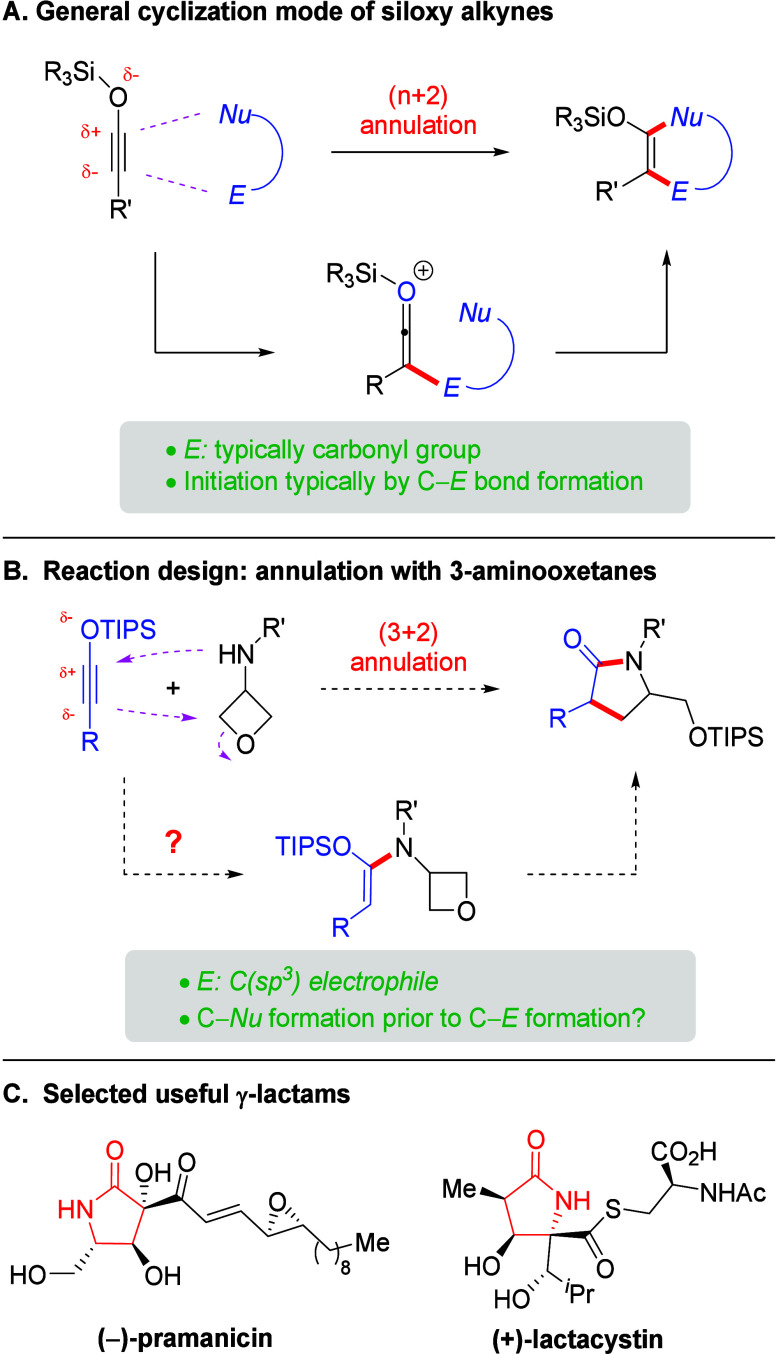
Introduction to Siloxy Alkyne Reactivity and Reaction Design

Oxetane is a highly useful strained heterocycle with broad application in medicinal chemistry and organic synthesis.^4^ In continuation of interests in the study of oxetanes,^5^ we have designed a type of amphoteric molecule, 3-aminooxetanes,^6,7^ that links oxetane and amine functionalities in the same molecule. They have been demonstrated to be versatile for the synthesis of useful cyclic molecules with electron-poor molecules, such as isocyanate, carbon disulfide, and CO_2_.^6^ Due to the low reactivity of oxetane (relative to aldehyde and ketone), particularly for intermolecular C–C bond formation, these reactions are normally initiated by nucleophilic addition of the amine functionality to the reaction partner, which generates a nucleophile *in situ* for intramolecular opening of the oxetane ring. However, its reactivity with an electron-rich species has not been demonstrated. Herein we report our study employing siloxy alkynes as electron-rich partners. We envisioned that, in the presence of a suitable Lewis acid catalyst, the alkyne hydroamination may proceed prior to C–C formation with the internal oxetane to form a lactam (Scheme 1B). Notably, the bond formation order might be different from those using carbonyl as the initiator shown in Scheme 1A. Indeed, after suitable optimization, we have achieved this process leading to intermolecular assembly of γ-butyrolactams, a type of important structures in medicinal chemistry and organic synthesis (Scheme 1C).^8,9^

We began to test our hypothesis with 3-aminooxetane **1a** and siloxy alkyne **2a** as the model substrates (see Table S1 for more details). Various Lewis acids were initially evaluated as potential catalysts (Table 1). Unfortunately, many of them did not give any desired products, including Sc­(OTf)_3_, IPrAuCl, AuCl_3_, and CF_3_CO_2_Ag (entries 1–4). However, AgSbF_6_ was able to provide the desired product **3a**, albeit in low yield (10%, entry 5). Further screening of other silver salts indicated that AgOTf is the most effective catalyst (59% yield, entry 6). AgNTf_2_ did not improve the results (entry 7). For comparison, the corresponding conjugate acid TfOH was not a good catalyst, suggesting that silver in AgOTf was responsible for the catalytic activity, rather than the *in situ* generated Brønsted acid (entry 8). HNTf_2_ did not improve the outcome either (entry 9). Changing the solvent to DCE did not make much difference, but if the catalyst loading was increased to 20 mol %, a big jump in yield was observed (82%, entry 11). Heating to a higher temperature resulted in a drop in yield (entry 12). We also evaluated other solvents, such as THF, dioxane, and EtOAc. Among them, EtOAc provided the highest efficiency (entry 15). It is worth mentioning that the product **3a** was formed as a single diastereomer in this process. The product identity revealed that the regiochemistry of this annulation was perfectly governed by matching the polarity of both reactants. Of note, in some of the above cases, the hydroamination product **3a’** was formed as a byproduct, which suggested that C–N bond formation preceded the oxetane opening.

**Table 1:**
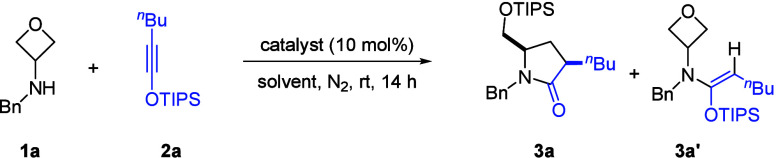
Evaluation of Conditions^a^

entry	catalyst	solvent	yield 3a (%)^b^
1	Sc(OTf)_3_	DCM	0
2	IPrAuCl	DCM	0
3	AuCl_3_	DCM	0
4	CF_3_CO_2_Ag	DCM	0
5	AgSbF_6_	DCM	12
6	AgOTf	DCM	59
7	AgNTf_2_	DCM	14
8	HOTf	DCM	0
9	HNTf_2_	DCM	0
10	AgOTf	DCE	63
11	AgOTf^c^	DCE	82
12^d^	AgOTf^c^	DCE	69
13	AgOTf^c^	THF	49
14	AgOTf^c^	1,4-dioxane	85
***15***	***AgOTf*** ^c^	***EtOAc***	***88***

a Reaction scale: **1a** (0.1 mmol), **2a** (1.5 equiv), catalyst (10–20 mol %), solvent (1.0 mL).

b Yield was determined by analysis of the ^1^H NMR spectra of the crude reaction mixture using mesitylene as an internal standard.

c 20 mol % of catalyst.

d Run at 70 °C.

A wide range of pyrrolidinone products **3** could be synthesized from the readily available substrates with excellent stereoselectivity (Scheme 2). Various functional groups, such as phthalimide, olefin, and aryl halide were well-tolerated. Heterocycles, including pyridine, furan, and thiophene could also be present in the substrates. It is noteworthy that the highly coordinating pyridine functionality also successfully led to the desired product. These pyrrolidinones represent an important skeleton widely present in numerous natural products and biologically active molecules.^8,9^ They are also valuable intermediates in organic synthesis. Thus, our intermolecular annulation represents an attractive convergent approach for the synthesis of these valuable building blocks.

**Scheme 2:**
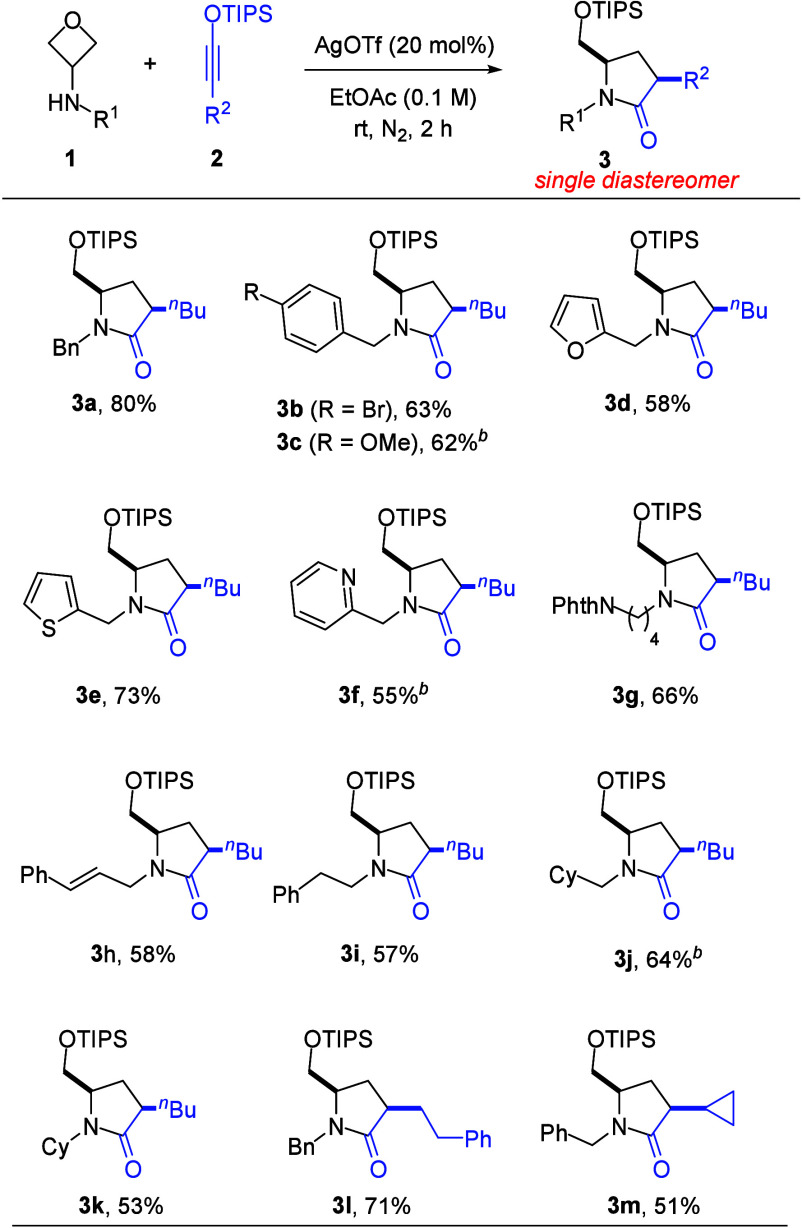
Reaction Scope^a^

a Reaction conditions: **1** (0.4 mmol), **2** (1.5 equiv), AgOTf (20 mol %), EtOAc (4.0 mL). Isolated yield.
b Run with DCE as solvent.

The pendant silyl ether motif in the product could be easily deprotected by TBAF. As shown in Scheme 3, after simple deprotection, the product **3c** could be converted to alcohol **3c’**, whose structure and relative stereochemistry were confirmed by X-ray crystallography. In a separate demonstration, the pendant hydroxymethyl group in the desilylated product could be easily oxidized to carboxylic acid **4**, which resembles the structure of a glutamate reuptake inhibitor and stimulator **5**.^10^ This example illustrates the expediency of our process in forming useful bioactive molecules.

**Scheme 3:**
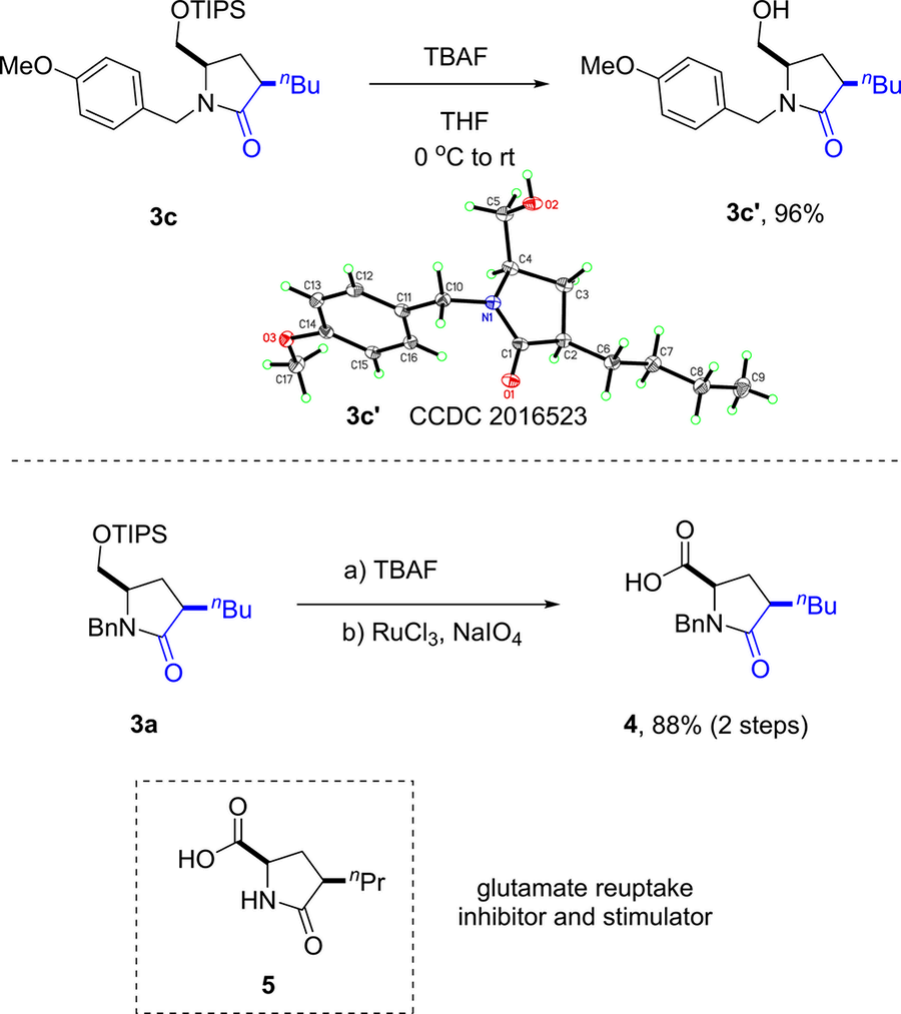
Product Derivatizations

A possible mechanism is proposed in Scheme 4. The silver catalyst can serve as a π-acid to activate the siloxy alkyne **1a** to form ketenium intermediate **IM1**, which is susceptible to nucleophilic attack by the amine **2a**.^11^ The intermolecular C–N bond formation results in intermediate **IM2**. Next, the oxetane ring opening is triggered by the internal carbon nucleophile to form intermediate **IM3**, which undergoes facile proton and silyl migration to form the observed lactam product **3a** with concomitant regeneration of the silver catalyst. During the study, we observed the formation of byproduct **3a’**, which might result from protodemtalation of **IM2** without ring-opening. Indeed, when **3a’** was treated with the AgOTf, it cannot lead to the desired product **3a**, suggesting that **3a’** is not a viable intermediate. Notably, in **IM2**, the Lewis basic oxetane motif may have favorable interaction with the silver center to dictate the *syn*-addition mode, which not only sets the correct stereochemistry for the subsequent oxetane opening by the C–Ag bond, but also activates the oxetane ring by lowering its LUMO level. This interaction is also consistent with the previous observation in related hydroamination of siloxy alkynes.^11^ Moreover, the formation of **3a’** also provided additional support of this proposed mechanism, particularly on the C–N bond formation prior to the oxetane ring-opening.

**Scheme 4:**
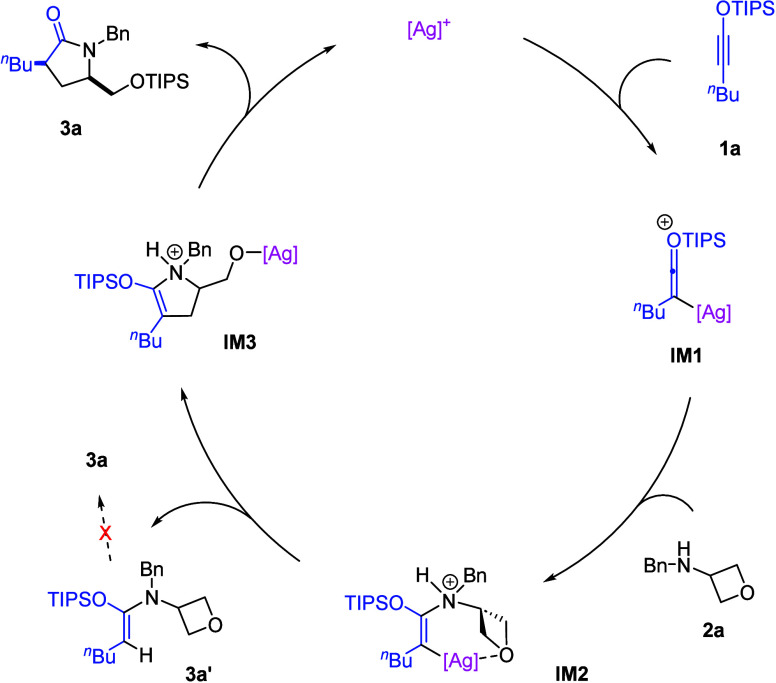
Proposed Mechanism

In summary, we have developed a new annulation process between siloxy alkynes and 3-aminooxetanes leading to convenient access to diversely functionalized γ-lactams. Different from previously related annulations of siloxy alkynes, this process employed amphoteric molecules bearing a less reactive electrophile motif, thus reversing the order of bond formation events. It features high regio- and stereoselectivity as well as mild conditions. The products are useful precursors to bioactive molecules. We believe that this study will contribute to a deeper understanding of the fundamental reactivity of both siloxy alkynes and 3-aminooxetanes.

## Supplementary Material





## Data Availability

The data underlying this study are available in the published article and its .

## Notes

Experimental procedure and compound characterization (PDF)
Crystal data (CIF)
