# Supplementary material for: Synthesis of γ‑Lactams by Intermolecular (3 + 2) Annulation of Siloxy Alkynes and 3‑Aminooxetanes
Source: Precis Chem. 2025 Apr 11;3(8):451–5. doi: 10.1021/prechem.5c00029 (PMC12381704; doi:10.1021/prechem.5c00029)
Supplement: Supplementary file 1 [file pc5c00029_si_001.pdf]

Supporting Information

Synthesis of γ-Lactams by Intermolecular (3+2) Annulation of Siloxy Alkynes and 3-Aminooxetanes

Xiang Li,[a†] Qiang Feng,[b†] Shuxuan Liu,[a] Hai Huang,[a] Zhengyu Han,[a*] and Jianwei Sun[b*]

[a]*Jiangsu Key Laboratory of Advanced Catalytic Materials & Technology, School of Petrochemical Engineering, Changzhou University, Changzhou, Jiangsu, 213164, China*

[b]*Department of Chemistry and the Hong Kong Branch of Chinese National Engineering Research Centre for Tissue Restoration & Reconstruction, The Hong Kong University of Science and Technology, Clear Water Bay, Kowloon, Hong Kong SAR, 999077, China*

Email to: sunjw@ust.hk, hanzhengyu@cczu.edu.cn

Table of Contents

I. General Information S-2

II. Substrate Preparation S-3

III. Ag-Catalyzed (3+2) Annulation with Siloxy Alkynes S-4

IV. Product Derivatizations S-15

V. Product Structure Determination S-18

NMR Spectra

I. General Information

All air or moisture sensitive reactions were conducted in oven-dried glassware under nitrogen atmosphere using dry solvents. Flash column chromatography was performed over silica gel (230-400 mesh) purchased from Qindao Puke Co., China. Anhydrous dichloromethane and tetrahydrofuran were purified by the Innovative® solvent purification system. ^{1}H and ^{13}C NMR spectra were collected on a Bruker AV 400 MHz NMR spectrometer using residue solvent peaks as an internal standard (^{1}H NMR: $CDCl_3$ at 7.26 ppm. ^{13}C NMR: $CDCl_3$ at 77.0 ppm). Mass spectra were collected on an Agilent GC/MS 5975C system, or a MALDI Micro MX mass spectrometer, or an API QSTAR XL System.

II. Substrate Preparation

Siloxy alkynes used in this work are all known compounds.[1] 3-Aminooxatanes were synthesized according to the following procedures and are known compounds.[2]

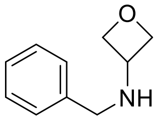

1a

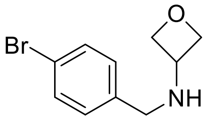

1b

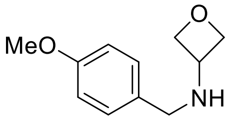

1c

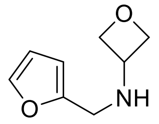

1d

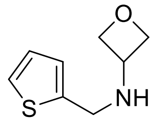

1e

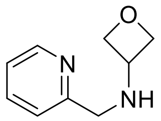

1f

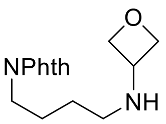

1g

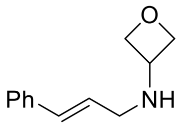

1h

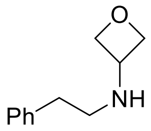

1i

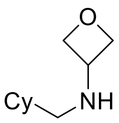

1j

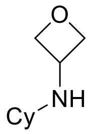

1k

(1) Wu, A.; Feng, Q.; Sung, H. H. Y.; Williams, I. D.; Sun, J. Synthesis of Eight-Membered Lactams through Formal [6+2] Cyclization of Siloxy Alkynes and Vinylazetidines. *Angew. Chem., Int. Ed.* **2019**, *58*, 6776–6780.

(2) Lai, Z.; Zhang, R.; Feng, Q.; Sun, J. 3-Aminooxetanes: Versatile 1,3-Amphoteric Molecules for Intermolecular Annulation Reactions. *Chem. Sci.* **2020**, *11*, 9945–9949.

III. Ag-Catalyzed (3+2) Annulation with Siloxy Alkynes

Table S1. Optimization of Conditions.[a]

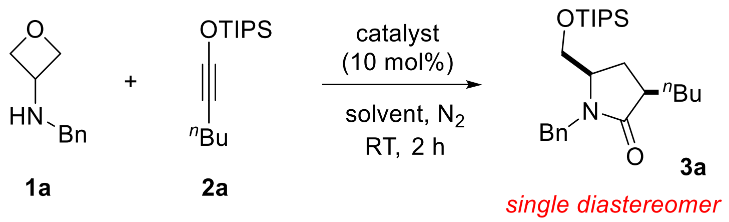

entry	catalyst	solvent	yield (%)[b]
1	AgOTf	DCM	59
2	$AgNTf_2$	DCM	14
3	HOTf	DCM	0
4	$HNTf_2$	DCM	0
5	$AgSbF_6$	DCM	12
6	$AgPF_6$	DCM	10
7	AgTFA	DCM	0
8	CuCl	DCM	0
9	$[Cu(MeCN)_3]PF_6$	DCM	0
10	$AuCl_3$	DCM	0
11	$Au(PPh_3)NTf_2$	DCM	0
12	IPrAuCl	DCM	0
13	$MesAuNTf_2$	DCM	0
14	$Sc(OTf)_3$	DCM	0
15	$PtBr_2$	DCM	0

16	AgOTf	DCE	63
17	AgOTf (20 mol%)	DCE	82
18	AgOTf (20 mol%)	DCE	69[b]
19	AgOTf (20 mol%)	THF	49
20	AgOTf (20 mol%)	1,4-dioxane	85
21	AgOTf (20 mol%)	EtOAc	88 (80)[c]

[a]Reaction scale: **1a** (0.1 mmol), **2a** (1.5 equiv), solvent (1.0 mL). Yield was determined by analysis of the ^{1}H NMR spectra of the crude reaction mixture using mesitylene as an internal standard. [b]Run at 70 ^{o}C. [c]Yield in parentheses is isolated yield.

General Procedure A

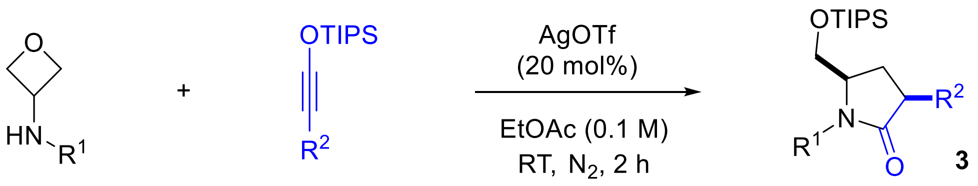

1 **2** *single diastereomer*

In a glove box, to an oven-dried 10-mL vial charged with 3-aminooxetane (0.4 mmol), siloxy alkyne (0.6 mmol), and anhydrous EtOAc (4 mL) was added AgOTf (20.6 mg, 0.08 mmol, 20 mol%). The vial was capped and removed from the glove box. The reaction mixture was diluted with EtOAc (10 mL) after stirring at room temperature for 2 h. Then a saturated aqueous NH_4Cl solution (10 mL) was added, and the aqueous layer was extracted with EtOAc (10 mL × 2). The combined organic layers were dried over anhydrous Na_2SO_4 and concentrated under reduced pressure. The residue was purified by silica gel flash column chromatography to give the desired product.

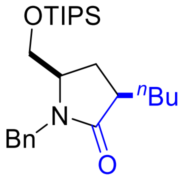

3a

1-Benzyl-3-butyl-5-(((triisopropylsilyl)oxy)methyl)pyrrolidin-2-one (3a) was prepared as a colorless oil from **1a** (65.2 mg, 0.4 mmol) and (hex-1-yn-1-yloxy)triisopropylsilane **2a** (152.4 mg, 0.6 mmol, 1.5 equiv) according to the General Procedure A (purification by silica gel chromatography, eluent: hexanes/EtOAc = 15:1→10:1) in 80% yield (134 mg).

^{1}H NMR (400 MHz, $CDCl_3$) δ 7.28 – 7.20 (m, 5H), 5.03 (d, J = 15.0 Hz, 1H), 4.07 (d, J = 15.0 Hz, 1H), 3.74 (dd, J = 10.6, 4.2 Hz, 1H), 3.65 (dd, J = 10.6, 3.9 Hz, 1H), 3.46 (tt, J = 7.8, 4.0 Hz, 1H), 2.45 – 2.41 (m, 1H), 2.20 – 2.13 (m, 1H), 1.97 – 1.94 (m, 1H), 1.61 – 1.53 (m ,1H), 1.35 – 1.33 (m, 5H), 1.07 – 1.02 (m, 21H), 0.90 (t, J = 6.6 Hz, 3H).

^{13}C NMR (100 MHz, $CDCl_3$) δ 177.5, 137.0, 128.4, 127.8, 127.2, 63.9, 56.8, 44.5, 41.3, 31.4, 29.4, 27.6, 22.5, 17.9, 13.9, 11.8.

IR (neat) 2940, 2865, 1689, 1457, 1420, 1385, 1356, 1247, 1126, 1073, 1005 cm^{-1}.

HRMS m/z (CI) calculated for $C_{25}H_{44}NO_2Si$ [M+H]$^+$: 418.3163, found: 418.3158.

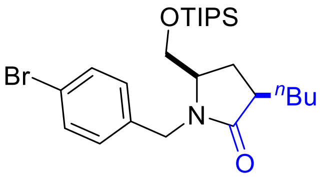

3b

1-(4-Bromobenzyl)-3-butyl-5-(((triisopropylsilyl)oxy)methyl)pyrrolidin-2-one (3b) was prepared as a colorless oil from **1b** (96.4 mg, 0.4 mmol) and (hex-1-yn-1-yloxy)triisopropylsilane **2a** (152.4 mg, 0.6 mmol, 1.5 equiv) according to the General Procedure A (purification by silica gel chromatography, eluent: hexanes/EtOAc = 15:1→10:1) in 63% yield (124 mg).

^{1}H NMR (400 MHz, $CDCl_3$) δ 7.39 (d, J = 8.3 Hz, 2H), 7.10 (d, J = 8.3 Hz, 2H),

4.90 (d, *J* = 15.1 Hz, 1H), 4.10 (d, *J* = 15.1 Hz, 1H), 3.72 (dd, *J* = 10.8, 3.8 Hz, 1H), 3.63 (dd, *J* = 10.7, 4.3 Hz, 1H), 3.46 (tt, *J* = 7.8, 4.1 Hz, 1H), 2.43 – 2.36 (m, 1H), 2.20 – 2.13 (m, 1H), 1.96 – 1.91 (m, 1H), 1.56 – 1.48 (m, 1H), 1.38 – 1.33 (m, 5H), 1.06 – 1.01 (m, 21H), 0.89 (t, *J* = 6.7 Hz, 3H).

^{13}C NMR (100 MHz, $CDCl_3$) δ 177.6, 136.2, 131.5, 129.6, 121.1, 64.3, 57.1, 44.1, 41.3, 31.3, 29.4, 27.6, 22.5, 17.9, 13.9, 11.8.

IR (neat) 2939, 2864, 1686, 1458, 1395, 1354, 1247, 1173, 1129, 1069, 1007 cm^{-1}.

HRMS m/z (CI) calculated for $C_{25}H_{43}BrNO_2Si$ [M+H]$^+$: 496.2241, found: 496.2223.

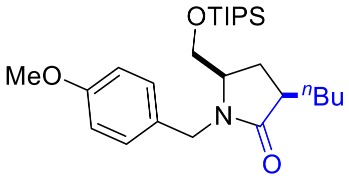

3c

3-Butyl-1-(4-methoxybenzyl)-5-(((triisopropylsilyl)oxy)methyl)pyrrolidin-2-one (3c) was prepared as a colorless oil from **1c** (77.2 mg, 0.4 mmol) and (hex-1-yn-1-yloxy)triisopropylsilane **2a** (152.4 mg, 0.6 mmol, 1.5 equiv) according to the General Procedure A except for DCE as solvent (purification by silica gel chromatography, eluent: hexanes/EtOAc = 15:1→10:1) in 62% yield (111 mg).

^{1}H NMR (400 MHz, $CDCl_3$) δ 7.15 (d, *J* = 8.6 Hz, 2H), 6.81 (d, *J* = 8.6 Hz, 2H), 4.97 (d, *J* = 14.8 Hz, 1H), 3.99 (d, *J* = 14.8 Hz, 1H), 3.77 – 3.72 (m, 4H), 3.65 (dd, *J* = 10.6, 3.9 Hz, 1H), 3.44 (tt, *J* = 7.7, 4.0 Hz, 1H), 2.42 – 2.35 (m, 1H), 2.18 – 2.11 (m, 1H), 1.97 – 1.92 (m, 1H), 1.59 – 1.54 (m, 1H), 1.39 – 1.32 (m, 5H), 1.08 – 1.00 (m, 21H), 0.89 (t, *J* = 6.8 Hz, 3H).

^{13}C NMR (100 MHz, $CDCl_3$) δ 177.4, 158.8, 129.2, 129.1, 113.8, 63.9, 56.6, 55.2, 43.8, 41.4, 31.4, 29.5, 27.6, 22.6, 17.9, 13.9, 11.9.

IR (neat) 2941, 2865, 1685, 1613, 1511, 1457, 1421, 1387, 1356, 1294, 1244, 1174, 1132, 1035 cm^{-1}.

HRMS m/z (CI) calculated for $C_{26}H_{46}NO_3Si$ $[M+H]^+$: 448.3241, found: 448.3239.

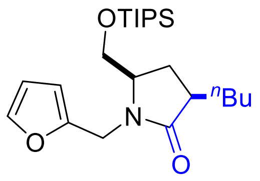

3d

3-Butyl-1-(furan-2-ylmethyl)-5-(((triisopropylsilyl)oxy)methyl)pyrrolidin-2-one (3d) was prepared as a colorless oil from **1d** (61.2 mg, 0.4 mmol) and (hex-1-yn-1-yloxy)triisopropylsilane **2a** (152.4 mg, 0.6 mmol, 1.5 equiv) according to the General Procedure A (purification by silica gel chromatography, eluent: hexanes/EtOAc = 15:1→10:1) in 58% yield (94 mg).

^{1}H NMR (400 MHz, $CDCl_3$) δ 7.32 – 7.29 (m, 1H), 6.27 (dd, *J* = 3.1, 1.9 Hz, 1H), 6.18 (d, *J* = 3.1 Hz, 1H), 4.97 (d, *J* = 15.6 Hz, 1H), 4.09 (d, *J* = 15.6 Hz, 1H), 3.86 (dd, *J* = 10.6, 4.2 Hz, 1H), 3.73 (dd, *J* = 10.6, 3.9 Hz, 1H), 3.52 (tt, *J* = 7.8, 4.0 Hz, 1H), 2.39 – 2.32 (m, 1H), 2.20 – 2.13 (m, 1H), 1.94 – 1.90 (m, 1H), 1.58 – 1.50 (m, 1H), 1.33 – 1.32 (m, 5H), 1.09 – 1.03 (m, 21H), 0.90 – 0.88 (m, 3H).

^{13}C NMR (100 MHz, $CDCl_3$) δ 177.3, 150.7, 141.9, 110.2, 107.9, 63.9, 57.1, 41.2, 37.4, 31.1, 29.4, 27.6, 22.6, 17.9, 13.9, 11.9.

IR (neat) 1979, 2943, 2867, 1697, 1458, 1385, 1355, 1292, 1251, 1133, 1076, 1011 cm^{-1}.

HRMS m/z (CI) calculated for $C_{23}H_{42}NO_3Si$ $[M+H]^+$: 408.2928, found: 408.2942.

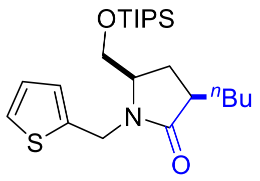

3e

3-Butyl-1-(thiophen-2-ylmethyl)-5-(((triisopropylsilyl)oxy)methyl)pyrrolidin-2-one (3e) was prepared as a colorless oil from **1e** (67.6 mg, 0.4 mmol) and (hex-1-yn-1-yloxy)triisopropylsilane **2a** (152.4 mg, 0.6 mmol, 1.5 equiv)

according to the A (purification by silica gel chromatography, eluent: hexanes/EtOAc = 15:1→10:1) in 73% yield (123 mg).

^{1}H NMR (400 MHz, $CDCl_3$) δ 7.16 (dd, *J* = 5.0, 0.9 Hz, 1H), 6.97 – 6.83 (m, 2H), 5.14 (d, *J* = 15.3 Hz, 1H), 4.31 (d, *J* = 15.3 Hz, 1H), 3.83 (dd, *J* = 10.7, 3.9 Hz, 1H), 3.71 (dd, *J* = 10.7, 4.4 Hz, 1H), 3.60 – 3.54 (m, 1H), 2.38 – 2.27 (m, 1H), 2.19 – 2.12 (m, 1H), 1.94 – 1.90 (m, 1H), 1.53 – 1.45 (m, 1H), 1.32 – 1.31 (m, 5H), 1.20 – 1.02 (m, 21H), 0.88 (t, *J* = 5.1 Hz, 3H).

^{13}C NMR (100 MHz, $CDCl_3$) δ 177.2, 139.3, 126.5, 126.3, 124.9, 64.5, 56.5, 41.2, 39.1, 31.2, 29.3, 27.5, 22.5, 17.9, 13.9, 11.8.

IR (neat) 2942, 2866, 1690, 1457, 1426, 1383, 1357, 1288, 1244, 1133, 1076, 1005 cm^{-1}.

HRMS m/z (CI) calculated for $C_{23}H_{42}NO_2SSi$ $[M+H]^+$: 424.2700, found: 424.2693.

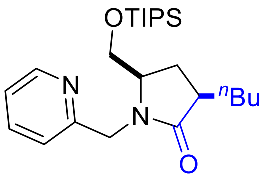

3f

3-Butyl-1-(pyridin-2-ylmethyl)-5-(((triisopropylsilyl)oxy)methyl)pyrrolidin-2-one (3f) was prepared as a colorless oil from **1f** (65.6 mg, 0.4 mmol) and (hex-1-yn-1-yloxy)triisopropylsilane **2a** (152.4 mg, 0.6 mmol, 1.5 equiv) according to the General Procedure A except that DCE was used as solvent (purification by silica gel chromatography, eluent: hexanes/EtOAc = 2:1→1:1) in 55% yield (92 mg).

^{1}H NMR (400 MHz, $CDCl_3$) δ 8.47 (dd, *J* = 6.8, 1.7 Hz, 2H), 7.58 (dt, *J* = 7.8, 1.8 Hz, 1H), 7.21 (dd, *J* = 7.8, 4.8 Hz, 1H), 4.92 (d, *J* = 15.2 Hz, 1H), 4.24 (d, *J* = 15.2 Hz, 1H), 3.75 (dd, *J* = 10.8, 3.6 Hz, 1H), 3.63 (dd, *J* = 10.8, 4.8 Hz, 1H), 3.53 – 3.44 (m, 1H), 2.42 – 2.35 (m, 1H), 2.21 – 2.14 (m, 1H), 1.95 – 1.90 (m, 1H), 1.52 – 1.45 (m, 1H), 1.33 – 1.32 (m, 5H), 1.04 – 1.00 (m, 21H), 0.88 (t, *J* = 6.8 Hz, 3H).

^{13}**C NMR** (100 MHz, $CDCl_3$) δ 177.7, 149.1, 148.6, 135.8, 132.9, 123.5, 64.7, 57.3, 42.3, 41.2, 31.3, 29.4, 27.6, 22.5, 17.9, 13.9, 11.8.

IR (neat) 2978, 2943, 2866, 1685, 1582, 1458, 1419, 1389, 1356, 1251, 1134, 1077, 1013 cm^{-1}.

HRMS m/z (CI) calculated for $C_{24}H_{43}N_2O_2Si$ $[M+H]^+$: 419.3088, found: 419.3077.

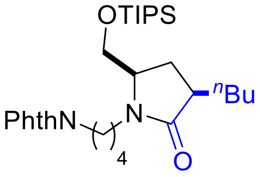

3g

2-(4-(3-Butyl-2-oxo-5-(((triisopropylsilyl)oxy)methyl)pyrrolidin-1-yl)butyl)isoindoline-1,3-dione (3g) was prepared as a colorless oil from **1g** (109.6 mg, 0.4 mmol) and (hex-1-yn-1-yloxy)triisopropylsilane **2a** (152.4 mg, 0.6 mmol, 1.5 equiv) according to the General Procedure A (purification by silica gel chromatography, eluent: hexanes/EtOAc = 4:1→2:1) in 66% yield (139 mg).

^{1}**H NMR** (400 MHz, $CDCl_3$) δ 7.77 – 7.74 (m, 2H), 7.67 – 7.63 (m, 2H), 3.73 (dd, J = 10.6, 3.4 Hz, 1H), 3.65 – 3.53 (m, 5H), 3.13 – 3.08 (m, 1H), 2.33 – 2.26 (m, 1H), 2.18 – 2.10 (m, 1H), 1.83 – 1.81 (m, 1H), 1.64 – 1.49 (m, 4H), 1.37 – 1.25 (m, 6H), 1.04 – 0.97 (m, 21H), 0.83 (t, J = 6.7 Hz, 3H).

^{13}**C NMR** (100 MHz, $CDCl_3$) δ 177.2, 168.1, 133.7, 132.0, 123.0, 65.2, 57.5, 41.1, 40.3, 37.5, 31.2, 29.4, 27.7, 26.0, 24.7, 22.5, 17.8, 13.8, 11.7.

IR (neat) 2977, 2942, 2866, 1771, 1709, 1682, 1458, 1390, 1365, 1248, 1134, 1073, 1003 cm^{-1}.

HRMS m/z (CI) calculated for $C_{30}H_{49}N_2O_4Si$ $[M+H]^+$: 529.3456, found: 529.3472.

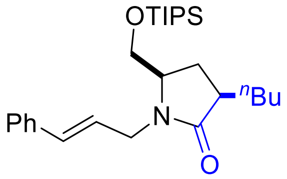

3h

3-Butyl-1-cinnamyl-5-(((triisopropylsilyl)oxy)methyl)pyrrolidin-2-one (3h) was prepared as a colorless oil from **1h** (70.8 mg, 0.4 mmol) and (hex-1-yn-1-yloxy)triisopropylsilane **2a** (152.4 mg, 0.6 mmol, 1.5 equiv) according to the General Procedure A (purification by silica gel chromatography, eluent: hexanes/EtOAc = 15:1→10:1) in 58% yield (103 mg).

^{1}H NMR (400 MHz, $CDCl_3$) δ 7.34 – 7.26 (m, 4H), 7.22 – 7.19 (m, 1H), 6.51 (d, J = 15.9 Hz, 1H), 6.14 (ddd, J = 15.9, 7.7, 5.4 Hz, 1H), 4.50 (ddd, J = 15.1, 5.3, 1.3 Hz, 1H), 3.83 – 3.73 (m, 3H), 3.69 – 3.63 (m, 1H), 2.42 – 2.36 (m, 1H), 2.22 (ddd, J = 12.7, 9.3, 7.6 Hz, 1H), 1.96 – 1.92 (m, 1H), 1.57 – 1.49 (m, 1H), 1.35 – 1.34 (m, 5H), 1.12 – 1.04 (m, 21H), 0.97 – 0.81 (m, 3 H).

^{13}C NMR (100 MHz, $CDCl_3$) δ 177.2, 136.5, 132.7, 128.4, 127.5, 126.3, 124.6, 64.6, 57.3, 43.0, 41.3, 31.4, 29.5, 27.7, 22.5, 17.9, 13.9, 11.8.

IR (neat) 2981, 2944, 2867, 1688, 1456, 1385, 1356, 1289, 1244, 1134, 1074, 1007 cm^{-1}.

HRMS m/z (CI) calculated for $C_{27}H_{46}NO_2Si$ $[M+H]^+$: 444.3292, found: 444.3306.

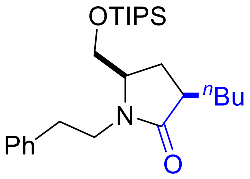

3i

3-Butyl-1-phenethyl-5-(((triisopropylsilyl)oxy)methyl)pyrrolidin-2-one (3i) was prepared as a colorless oil from **1i** (70.8 mg, 0.4 mmol) and (hex-1-yn-1-yloxy)triisopropylsilane **2a** (152.4 mg, 0.6 mmol, 1.5 equiv) according to the General Procedure A (purification by silica gel chromatography, eluent: hexanes/EtOAc = 15:1→10:1) in 57% yield (98 mg).

^{1}**H NMR** (400 MHz, $CDCl_3$) δ7.29 – 7.17 (m, 5H), 3.87 (ddd, J = 13.8, 9.1, 6.5 Hz, 1H), 3.73 (dd, J = 10.7, 3.9 Hz, 1H), 3.66 (dd, J = 10.7, 4.7 Hz, 1H), 3.51 – 3.45 (m, 1H), 3.38 – 3.32 (m, 1H), 2.95 – 2.78 (m, 2H), 2.31 – 2.28 (m, 1H), 2.19 – 2.12 (m, 1H), 1.92 – 1.90 (m, 1H), 1.45 – 1.32 (m, 6H), 1.13 – 1.01 (m, 21H), 0.90 (t, J = 6.6 Hz, 3H).

^{13}**C NMR** (100 MHz, $CDCl_3$) δ 177.2, 139.1, 128.7, 128.3, 126.2, 65.0, 57.9, 42.4, 41.2, 33.7, 31.3, 29.4, 27.9, 22.5, 17.9, 14.0, 11.8.

IR (neat) 2982, 2945, 2869, 1691, 1455, 1386, 1356, 1294, 1135, 1077 cm^{-1}.

HRMS m/z (CI) calculated for $C_{26}H_{46}NO_2Si$ $[M+H]^+$: 432.3292, found: 432.3305.

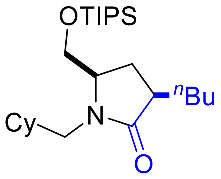

3j

3-Butyl-1-(cyclohexylmethyl)-5-(((triisopropylsilyl)oxy)methyl)pyrrolidin-2-one (3j) was prepared as a colorless oil from **1j** (67.6 mg, 0.4 mmol) and (hex-1-yn-1-yloxy)triisopropylsilane **2a** (152.4 mg, 0.6 mmol, 1.5 equiv) according to the General Procedure A except that DCE was used as solvent (purification by silica gel chromatography, eluent: hexanes/EtOAc = 15:1→10:1) in 64% yield (109 mg).

^{1}**H NMR** (400 MHz, $CDCl_3$) δ 3.75 (dd, J = 10.7, 3.6 Hz, 1H), 3.66 (dd, J = 10.6, 4.8 Hz, 1H), 3.61 – 3.52 (m, 1H), 3.47 (dd, J = 13.6, 9.2 Hz, 1H), 2.89 (dd, J = 13.6, 5.7 Hz, 1H), 2.35 – 2.31 (m, 1H), 2.20 – 2.12 (m, 1H), 1.91 – 1.83 (m, 1H), 1.66 – 1.48 (m, 7H), 1.42 – 1.22 (m, 7H), 1.13 – 1.01 (m, 24H), 0.88 – 0.86 (m, 3H).

^{13}**C NMR** (100 MHz, $CDCl_3$) δ 177.3, 65.0, 57.8, 46.8, 41.2, 35.6, 31.3, 31.1, 30.4, 29.5, 27.9, 26.4, 25.8, 25.7, 22.5, 17.9, 13.9, 11.8.

IR (neat) 2981, 2929, 2864, 1688, 1455, 1384, 1249, 1134, 1074, 1005 cm^{-1}.

HRMS m/z (CI) calculated for $C_{25}H_{50}NO_2Si$ $[M+H]^+$: 424.3605, found: 424.3619.

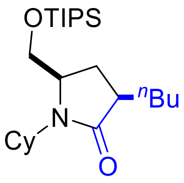

3k

3-Butyl-1-cyclohexyl-5-(((triisopropylsilyl)oxy)methyl)pyrrolidin-2-one (3k) was prepared as a colorless oil from **1k** (62 mg, 0.4 mmol) and (hex-1-yn-1-yloxy)triisopropylsilane **2a** (152.4 mg, 0.6 mmol, 1.5 equiv) according to the General Procedure A (purification by silica gel chromatography, eluent: hexanes/EtOAc = 15:1→10:1) in 53% yield (89 mg).

^{1}H NMR (400 MHz, $CDCl_3$) δ 3.85 – 3.81 (m, 1H), 3.72 – 3.62 (m, 3H), 2.31 – 2.17 (m, 2H), 1.94 – 1.59 (m, 8H), 1.57 – 1.51 (m, 1H), 1.34 – 1.25 (m, 7H), 1.14 – 1.05 (m, 22H), 0.91 – 0.89 (m, 3H).

^{13}C NMR (100 MHz, $CDCl_3$) δ 177.5, 66.5, 58.0, 53.2, 41.6, 32.0, 30.9, 29.7, 29.6, 28.0, 26.0, 25.9, 25.5, 22.6, 17.9, 13.9, 11.9.

IR (neat 2981, 2941, 2867, 1688, 1456, 1385, 1292, 1251, 1135, 1077 cm^{-1}.

HRMS m/z (CI) calculated for $C_{24}H_{48}NO_2Si$ [M+H]$^+$: 410.3449, found: 410.3450.

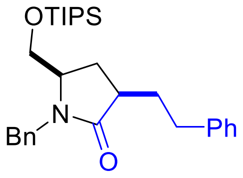

3l

1-Benzyl-3-phenethyl-5-(((triisopropylsilyl)oxy)methyl)pyrrolidin-2-one (3l) was prepared as a colorless oil from **1a** (65.2 mg, 0.4 mmol) and triisopropyl((4-phenylbut-1-yn-1-yl)oxy)silane **2b** (181.2 mg, 0.6 mmol, 1.5 equiv) according to the General Procedure A (purification by silica gel chromatography, eluent: hexanes/EtOAc = 15:1→10:1) in 71% yield (132 mg).

^{1}H NMR (400 MHz, $CDCl_3$) δ 7.33 – 7.18 (m, 10H), 5.09 (d, J = 15.0 Hz, 1H), 4.10 (d, J = 15.0 Hz, 1H), 3.79 (dd, J = 10.7, 4.1 Hz, 1H), 3.68 (dd, J = 10.7, 3.7 Hz,

1H), 3.52 – 3.46 (m, 1H), 2.85 – 2.70 (m, 2H), 2.51 – 2.42 (m, 1H), 2.41 – 2.31 (m, 1H), 2.24– 2.17 (m, 1H), 1.79 – 1.61 (m, 2H), 1.13 – 1.07 (m, 21H).

^{13}C NMR (100 MHz, $CDCl_3$) δ 177.1, 141.5, 136.8, 128.4, 128.3, 128.2, 127.8, 127.2, 125.7, 63.6, 56.7, 44.4, 40.6, 33.4 (2C), 27.6, 17.9, 11.8.

IR (neat) 2982, 2946, 2869, 1689, 1450, 1386, 1356, 1295, 1252, 1135, 1077 cm^{-1}.

HRMS m/z (CI) calculated for $C_{29}H_{44}NO_2Si$ [M+H]$^+$: 466.3136, found: 466.3146.

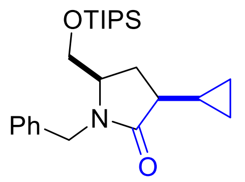

3m

1-Benzyl-3-cyclopropyl-5-(((triisopropylsilyl)oxy)methyl)pyrrolidin-2-one (3m) was prepared as a colorless oil from **1a** (65.2 mg, 0.4 mmol) and ((cyclopropylethynyl)oxy)triisopropylsilane **2c** (164.4 mg, 0.6 mmol, 1.5 equiv) according to the General Procedure A (purification by silica gel chromatography, eluent: hexanes/EtOAc = 15:1→10:1) in 51% yield (81 mg).

^{1}H NMR (400 MHz, $CDCl_3$) δ 7.31 – 7.21 (m, 5H), 5.04 (d, *J* = 15.0 Hz, 1H), 4.09 (d, *J* = 15.0 Hz, 1H), 3.75 (dd, *J* = 10.6, 4.4 Hz, 2H), 3.67 (dd, *J* = 10.6, 4.0 Hz, 1H), 3.46 – 3.40 (m, 1H), 2.13 (ddd, *J* = 12.3, 9.4, 7.6 Hz, 1H), 2.04 (dd, *J* = 17.3, 8.4 Hz, 1H), 1.66 (dt, *J* = 12.3, 8.0 Hz, 1H), 1.08 – 0.99 (m, 21H), 0.67 – 0.59 (m, 1H), 0.50 – 0.40 (m, 2H), 0.26 – 0.20 (m, 1H).

^{13}C NMR (100 MHz, $CDCl_3$) δ 176.6, 136.9, 128.4, 127.9, 127.2, 64.0, 56.5, 44.8, 44.5, 27.1, 17.9, 12.8, 11.8, 3.6, 1.7.

IR (neat) 2983, 2870, 1693, 1452, 1386, 1294, 1136, 1077 cm^{-1}.

HRMS m/z (CI) calculated for $C_{24}H_{40}NO_2Si$ [M+H]$^+$: 402.2823, found: 402.2834.

IV. Product Derivatizations

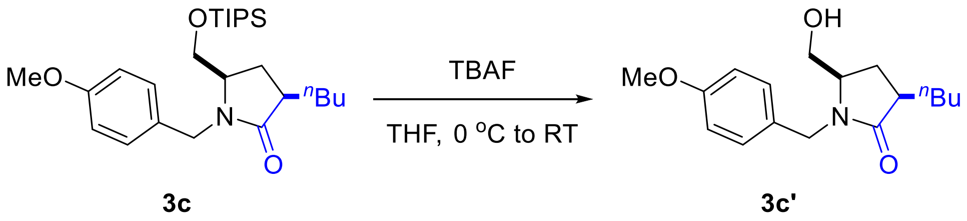

3-Butyl-5-(hydroxymethyl)-1-(4-methoxybenzyl)pyrrolidin-2-one (3c′). At 0 °C, to a solution of product **3c** (112 mg, 0.25 mmol) in THF (1 mL) was added TBAF (0.3 mL, 0.3 mmol, 1 M in THF). The reaction mixture was allowed to warm to room temperature and stir for 3 h. Diethyl ether (10 mL) and a saturated aqueous NH_4Cl solution (10 mL) were added. The layers were separated, and aqueous layer was extracted with diethyl ether (10 mL × 2). The combined organic layers were washed with brine and dried over anhydrous Na_2SO_4. The solvent was removed under reduced pressure, and the residue was purified by silica gel flash column chromatography (eluent: hexanes/EtOAc = 4:1→1:1) to give the desired product as a white solid in 96% yield (70 mg).

^{1}H NMR (400 MHz, $CDCl_3$) δ 7.15 (d, J = 8.5 Hz, 2H), 6.80 (d, J = 8.5 Hz, 2H), 4.69 (d, J = 14.8 Hz, 1H), 4.18 (d, J = 14.8 Hz, 1H), 3.74 – 3.71 (m, 4H), 3.48 – 3.41 (m, 2H), 2.65 (s, 1H), 2.41 – 2.34 (m, 1H), 2.17 – 2.10 (m, 1H), 1.95 – 1.93 (m, 1H), 1.56 (dt, J = 12.8, 8.8 Hz, 1H), 1.36 – 1.29 (m, 5H), 0.88 (t, J = 6.3 Hz, 3H).

^{13}C NMR (100 MHz, $CDCl_3$) δ 178.0, 158.9, 129.1, 129.0, 114.0, 62.1, 57.2, 55.1, 44.0, 41.5, 31.2, 29.4, 27.1, 22.5, 13.9.

IR (neat) 3389, 2984, 2870, 1661, 1625, 1511, 1450, 1386, 1355, 1295, 1246, 1136, 1082, 1035 cm^{-1}.

HRMS m/z (CI) calculated for $C_{17}H_{26}NO_3$ [M+H]$^+$: 292.1907, found: 292.1909.

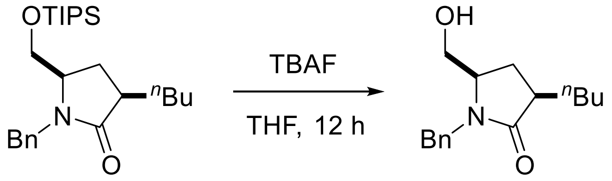

3a **S4**

1-Benzyl-3-butyl-5-(hydroxymethyl)pyrrolidin-2-one (S4). At 0 °C, to a solution of **3a** (805 mg, 1.8 mmol) in THF (20 mL) was added TBAF (2.0 mL, 2.0 mmol, 1 M in THF). The reaction mixture was allowed to warm to room temperature and stir for 12 h. Diethyl ether (20 mL) and a saturated aqueous NH_4Cl solution (20 mL) were added. The layers were separated, and the aqueous layer was extracted with diethyl ether (20 mL × 2). The combined organic layers were washed with brine and dried over anhydrous Na_2SO_4. The solvent was removed under reduced pressure, and the residue was purified by silica gel flash column chromatography (eluent: hexanes/EtOAc = 4:1→2:1) to give alcohol **S4** as a colorless oil in 91% yield (427 mg).

^{1}H NMR (400 MHz, $CDCl_3$) δ 7.28 – 7.18 (m, 5H), 4.81 (d, J = 15.0 Hz, 1H), 4.18 (d, J = 15.0 Hz, 1H), 3.70 (dd, J = 12.5, 4.1 Hz, 1H), 3.47 – 3.41 (m, 2H), 3.19 (s, 1H), 2.39 (dt, J = 13.2, 7.6 Hz, 1H), 2.13 (ddd, J = 12.7, 9.3, 7.4 Hz, 1H), 1.96 – 1.83 (m, 1H), 1.60 – 1.52 (m, 1H), 1.42 – 1.20 (m, 5H), 0.88 (t, J = 6.4 Hz, 3H).

^{13}C NMR (100 MHz, $CDCl_3$) δ 178.1, 136.8, 128.5, 127.7, 127.3, 62.0, 57.1, 44.5, 41.4, 31.1, 29.4, 27.2, 22.4, 13.9.

IR (neat) 2934, 2863, 1687, 1457, 1420, 1346, 1248, 1176, 1118, 1069, 1004 cm^{-1}.

HRMS m/z (CI) calculated for $C_{17}H_{24}NO_2$ [M+H]$^+$: 262.1802, found: 262.1794.

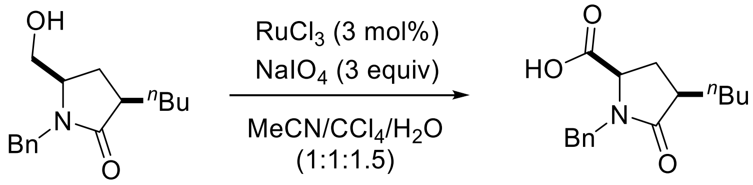

S4 **4**

1-Benzyl-4-butyl-5-oxopyrrolidine-2-carboxylic acid (4). To a solution of **S4**

(418 mg, 1.6 mmol) and $NaIO_4$ (684 mg, 3.2 mmol, 2 equiv) in a mixed solvent ($MeCN/CCl_4/H_2O$, 1/1/1.5, 17.5 mL) was added $RuCl_3•2H_2O$ (7.2 mg, 32 μmol, 2 mol%). After stirring at room temperature for 12 h, $^{i}PrOH$ (5 mL) was added and the mixture was stirred for 30 min before it was filtered through a pad of celite. The filtrate was concentrated under reduced pressure. The residue was diluted with ethyl acetate (40 mL). The resulting mixture was extracted with a saturated aqueous $NaHCO_3$ solution (30 mL × 2). To the combined aqueous layers was added an aqueous HCl solution (1 M) to adjust the pH to 3~4. Then the aqueous mixture was extracted with EtOAc (40 mL × 2). The combined organic layers were dried over anhydrous Na_2SO_4 and concentrated under reduced pressure to afford pure acid **4** as a light yellow oil in 97% yield (426 mg).

^{1}H NMR (400 MHz, $CDCl_3$) δ 10.23 (s, 1H), 7.33 – 7.19 (m, 5H), 5.23 (d, J = 14.8 Hz, 1H), 4.03 (d, J = 14.8 Hz, 1H), 3.95 (dd, J = 8.8, 6.2 Hz, 1H), 2.60 – 2.47 (m, 2H), 1.92 – 1.79 (m, 2H), 1.45 – 1.25 (m, 5H), 0.89 (t, J = 6.8 Hz, 3H).

^{13}C NMR (100 MHz, $CDCl_3$) δ 178.3, 174.7, 135.2, 128.8, 18.5, 127.8, 56.9, 45.6, 41.4, 31.1, 29.3, 28.8, 22.4, 13.9.

IR (neat) 3031, 2928, 2864, 1736, 1691, 1637, 1495, 1445, 1354, 1234, 1170, 1121, 1081 cm^{-1}.

HRMS m/z (CI) calculated for $C_{16}H_{22}NO_3$ $[M+H]^+$: 276.1594, found: 276.1595.

V. Product Structure Determination

The structures of the products **3c′** were determined by X-ray crystallography. The X-ray data have been deposited at the Cambridge Crystallographic Data Center (CCDC 2016523). The structures of other products were assumed by analogy.

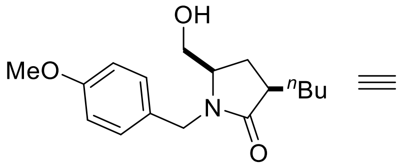

3c'

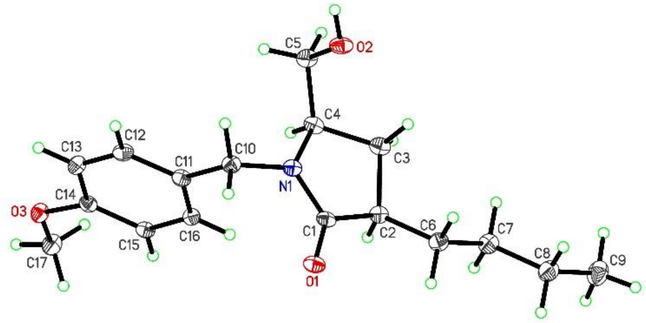

Table S2. Crystal data and structure refinement for 3c'.

Identification code	**3c'**
Empirical formula	$C_{17}H_{25}NO_3$
Formula weight	291.38
Temperature/K	100.01(10)
Crystal system	monoclinic
Space group	$P2_1/c$
a/Å	24.6267(8)
b/Å	7.3138(2)
c/Å	9.0880(3)
α/°	90
β/°	97.077(3)
γ/°	90
Volume/Å^3	1624.40(9)
Z	4
ρ_{calc}g/cm^3	1.191
μ/mm^{-1}	0.648
F(000)	632.0
Crystal size/mm^3	0.25 × 0.2 × 0.03
Radiation	CuKα (λ = 1.54184)
2Θ range for data collection/°	7.234 to 143.828

Index ranges	$-30 \le h \le 30$, $-8 \le k \le 8$, $-11 \le l \le 6$
Reflections collected	8481
Independent reflections	3112 [R_{int} = 0.0413, R_{sigma} = 0.0500]
Data/restraints/parameters	3112/0/193
Goodness-of-fit on F^2	1.024
Final R indexes [I>=2σ (I)]	R_1 = 0.0474, wR_2 = 0.1097
Final R indexes [all data]	R_1 = 0.0655, wR_2 = 0.1179
Largest diff. peak/hole / e $Å^{-3}$	0.20/-0.24

Table S3. Fractional Atomic Coordinates (×10^4) and Equivalent Isotropic Displacement Parameters ($Å^2$×10^3) for 3c'. U_{eq} is defined as 1/3 of the trace of the orthogonalised U_{IJ} tensor.

Atom	*x*	*y*	*z*	U(eq)
O1	2481.0(5)	6861.6(18)	6201.5(14)	22.1(3)
O2	2152.3(6)	408.4(18)	5697.9(14)	24.2(3)
O3	4539.6(5)	5409.5(18)	1255.0(14)	21.3(3)
N1	2572.6(6)	4041(2)	5165.2(16)	18.4(3)
C1	2300.2(8)	5590(2)	5373.7(18)	17.9(4)
C2	1754.3(8)	5565(2)	4412.0(19)	19.0(4)
C3	1691.2(8)	3556(3)	3946(2)	22.0(4)
C4	2280.3(8)	2859(3)	4009.8(19)	19.9(4)
C5	2352.5(8)	838(3)	4342(2)	21.9(4)
C6	1287.6(8)	6382(3)	5162(2)	22.3(4)
C7	759.3(8)	6561(3)	4118(2)	24.3(4)
C8	289.5(8)	7383(3)	4839(2)	30.3(5)
C9	-237.2(9)	7566(3)	3793(3)	34.9(5)
C10	3152.9(8)	3848(3)	5667(2)	20.2(4)
C11	3507.0(8)	4286(3)	4456.7(19)	19.4(4)
C12	4019.7(8)	3465(3)	4466(2)	21.7(4)
C13	4354.5(8)	3870(3)	3396(2)	21.7(4)
C14	4179.9(8)	5113(2)	2275(2)	18.7(4)
C15	3672.6(7)	5948(2)	2235.5(19)	18.0(4)
C16	3341.0(8)	5527(2)	3332(2)	18.7(4)
C17	4342.8(8)	6456(3)	-35(2)	24.9(4)

Table S4. Anisotropic Displacement Parameters ($Å^2$×10^3) for 3c'. The Anisotropic displacement factor exponent takes the form: $-2\pi^2[h^2a^{*2}U_{11}+2hka^*b^*U_{12}+…]$.

Atom	U_{11}	U_{22}	U_{33}	U_{23}	U_{13}	U_{12}

O1	28.6(7)	17.7(7)	19.1(6)	-0.3(5)	-0.8(5)	-3.0(6)
O2	39.2(8)	16.4(7)	18.4(6)	1.9(5)	9.0(6)	-0.7(6)
O3	21.9(7)	23.4(7)	19.3(6)	3.2(5)	5.5(5)	2.8(5)
N1	23.3(8)	17.1(7)	14.8(7)	0.6(6)	2.9(6)	-1.0(6)
C1	25.7(10)	15.6(9)	13.1(8)	3.4(7)	5.1(7)	-3.0(7)
C2	24.3(9)	19.2(9)	13.9(8)	1.1(7)	3.8(7)	-0.4(7)
C3	28.6(10)	20.5(10)	17.0(9)	-1.3(7)	2.6(7)	-2.9(8)
C4	26.1(10)	19.6(9)	14.1(8)	-0.2(7)	2.9(7)	-4.0(8)
C5	31.1(10)	20.9(9)	14.7(9)	-1.9(7)	6.7(7)	-0.5(8)
C6	25.1(10)	22.3(10)	19.4(9)	-3.6(7)	2.1(7)	-0.3(8)
C7	26.8(10)	25.3(10)	20.9(9)	2.7(8)	3.4(8)	2.0(8)
C8	26.9(11)	27.5(11)	36.4(11)	-5.4(9)	3.1(9)	2.2(9)
C9	29.8(12)	38.3(13)	37.3(12)	11.3(10)	6.3(9)	8.9(10)
C10	23.0(10)	19.1(9)	18.3(9)	4.1(7)	2.4(7)	0.6(7)
C11	22.9(10)	18.3(9)	16.8(9)	1.1(7)	1.7(7)	-1.9(8)
C12	27.5(10)	18.4(9)	18.8(9)	4.5(7)	0.9(7)	1.4(8)
C13	20.6(9)	21.9(9)	22.5(9)	1.0(8)	1.8(7)	3.6(8)
C14	21.5(9)	18.1(9)	16.7(9)	-2.0(7)	3.6(7)	-3.2(7)
C15	22.5(9)	16.4(8)	14.6(8)	1.6(7)	0.2(7)	-1.6(7)
C16	19.0(9)	18.4(9)	18.4(8)	-0.1(7)	1.8(7)	0.3(7)
C17	27.5(10)	27.7(10)	20.6(9)	5.3(8)	7.0(8)	3.8(8)

Table S5. Bond Lengths for 3c'.

Atom	Atom	Length/Å	Atom	Atom	Length/Å
O1	C1	1.244(2)	C4	C5	1.515(3)
O2	C5	1.418(2)	C6	C7	1.519(3)
O3	C14	1.376(2)	C7	C8	1.522(3)
O3	C17	1.434(2)	C8	C9	1.517(3)
N1	C1	1.341(2)	C10	C11	1.519(2)
N1	C4	1.477(2)	C11	C12	1.397(3)
N1	C10	1.452(2)	C11	C16	1.391(3)
C1	C2	1.511(3)	C12	C13	1.382(3)
C2	C3	1.532(3)	C13	C14	1.393(3)
C2	C6	1.528(2)	C14	C15	1.387(3)
C3	C4	1.532(3)	C15	C16	1.398(2)

Table S6. Bond Angles for 3c'.

Atom	Atom	Atom	Angle/°	Atom	Atom	Atom	Angle/°
C14	O3	C17	117.02(15)	C7	C6	C2	112.85(15)
C1	N1	C4	112.73(15)	C6	C7	C8	113.78(16)
C1	N1	C10	121.85(16)	C9	C8	C7	113.70(18)
C10	N1	C4	122.71(15)	N1	C10	C11	112.49(15)
O1	C1	N1	124.67(18)	C12	C11	C10	120.26(16)
O1	C1	C2	125.84(17)	C16	C11	C10	121.80(17)
N1	C1	C2	109.45(15)	C16	C11	C12	117.93(16)
C1	C2	C3	102.96(15)	C13	C12	C11	121.39(17)
C1	C2	C6	113.68(15)	C12	C13	C14	119.80(17)
C6	C2	C3	116.25(16)	O3	C14	C13	115.42(16)
C2	C3	C4	104.20(15)	O3	C14	C15	124.43(17)
N1	C4	C3	102.02(14)	C15	C14	C13	120.15(16)
N1	C4	C5	113.25(15)	C14	C15	C16	119.18(17)
C5	C4	C3	114.82(16)	C11	C16	C15	121.55(17)
O2	C5	C4	110.14(15)				

Table S7. Hydrogen Bonds for 3c'.

D	H	A	d(D-H)/Å	d(H-A)/Å	d(D-A)/Å	D-H-A/°
O2	H2	O1[1]	0.82	1.93	2.7390(19)	169.8

[1]+X,-1+Y,+Z

Table S8. Torsion Angles for 3c'.

A	B	C	D	Angle/°	A	B	C	D	Angle/°
O1	C1	C2	C3	-167.09(16)	C4	N1	C1	C2	3.80(19)
O1	C1	C2	C6	-40.5(2)	C4	N1	C10	C11	65.3(2)
O3	C14	C15	C16	179.59(17)	C6	C2	C3	C4	-151.66(16)
N1	C1	C2	C3	14.90(18)	C6	C7	C8	C9	179.81(18)
N1	C1	C2	C6	141.52(16)	C10	N1	C1	O1	-12.4(3)
N1	C4	C5	O2	59.9(2)	C10	N1	C1	C2	165.59(15)
N1	C10	C11	C12	-151.98(17)	C10	N1	C4	C3	177.65(15)
N1	C10	C11	C16	29.3(2)	C10	N1	C4	C5	53.7(2)
C1	N1	C4	C3	-20.73(18)	C10	C11	C12	C13	-178.60(18)
C1	N1	C4	C5	-144.70(16)	C10	C11	C16	C15	178.88(17)
C1	N1	C10	C11	-94.67(19)	C11	C12	C13	C14	-0.4(3)
C1	C2	C3	C4	-26.69(17)	C12	C11	C16	C15	0.2(3)

C1	C2	C6	C7	172.86(15)	C12	C13	C14	O3	-179.33(17)
C2	C3	C4	N1	28.50(17)	C12	C13	C14	C15	0.3(3)
C2	C3	C4	C5	151.40(15)	C13	C14	C15	C16	0.0(3)
C2	C6	C7	C8	-179.67(16)	C14	C15	C16	C11	-0.2(3)
C3	C2	C6	C7	-67.8(2)	C16	C11	C12	C13	0.1(3)
C3	C4	C5	O2	-56.8(2)	C17	O3	C14	C13	170.73(16)
C4	N1	C1	O1	-174.24(16)	C17	O3	C14	C15	-8.9(3)

Table S9. Hydrogen Atom Coordinates (Å×10^4) and Isotropic Displacement Parameters (Å^2×10^3) for 3c'.

**Atom**	***x***	***y***	***z***	**U(eq)**
H2	2217	-667	5905	36
H2A	1790	6289	3522	23
H3A	1500	3448	2950	26
H3B	1492	2878	4623	26
H4	2415	3122	3061	24
H5A	2156	130	3544	26
H5B	2737	519	4406	26
H6A	1223	5614	5993	27
H6B	1396	7581	5549	27
H7A	651	5360	3735	29
H7B	827	7321	3284	29
H8A	221	6621	5671	36
H8B	398	8582	5225	36
H9A	-186	8432	3028	52
H9B	-524	7984	4335	52
H9C	-335	6400	3354	52
H10A	3226	2604	6005	24
H10B	3251	4658	6502	24
H12	4138	2629	5208	26
H13	4695	3314	3425	26
H15	3555	6778	1489	22
H16	3001	6090	3308	22
H17A	4614	6465	-708	37
H17B	4271	7687	252	37
H17C	4012	5917	-513	37

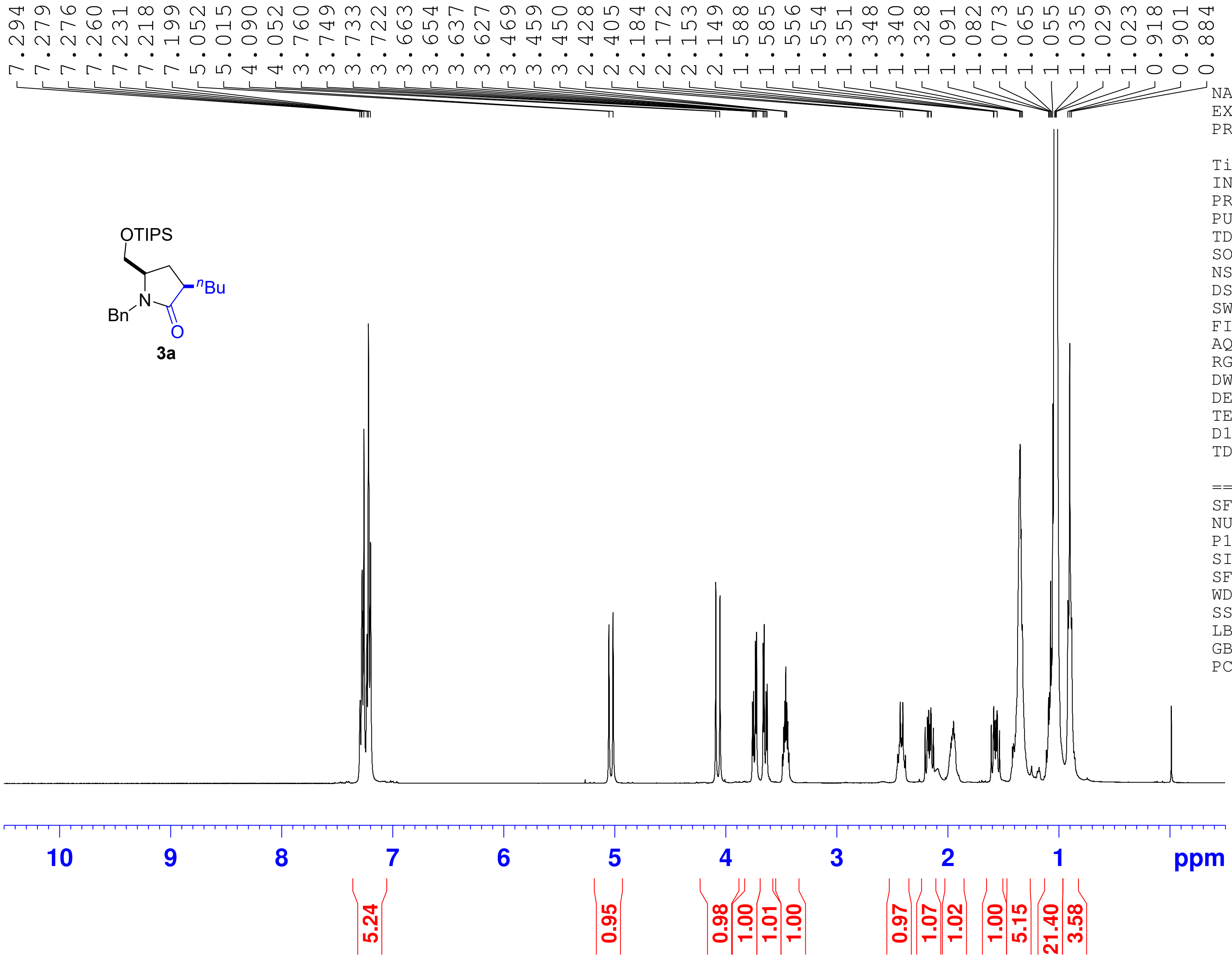


```
NAME                    8-149
EXPNO                       1
PROCNO                      1

Time                    22.14
INSTRUM                 spect
PROBHD      5 mm PABBO BB/
PULPROG                  zg30
TD                      65536
SOLVENT                 CDCl3
NS                          8
DS                          0
SWH                  8012.820 Hz
FIDRES               0.122266 Hz
AQ                  4.0894966 sec
RG                      17.38
DW                     62.400 usec
DE                       6.50 usec
TE                      297.3 K
D1                 1.00000000 sec
TD0                         1

======== CHANNEL f1 ========
SFO1              400.1324710 MHz
NUC1                       1H
P1                      14.50 usec
SI                      65536
SF                400.1300098 MHz
WDW                        EM
SSB                         0
LB                       0.30 Hz
GB                          0
PC                       1.00
```

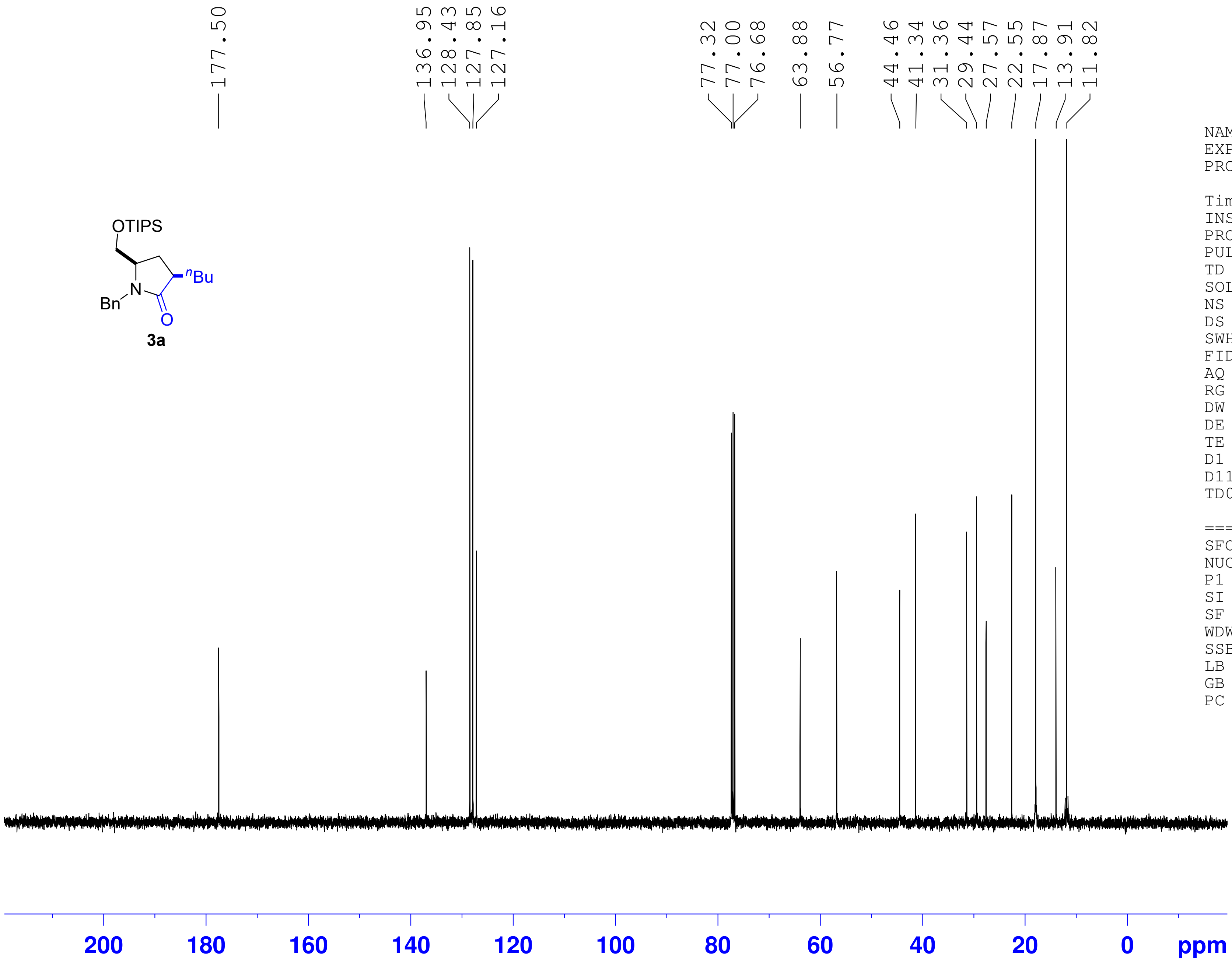

NAME 8-149
EXPNO 2
PROCNO 1

Time 22.17
INSTRUM spect
PROBHD 5 mm PABBO BB/
PULPROG zgpg30
TD 65536
SOLVENT CDCl3
NS 48
DS 0
SWH 24038.461 Hz
FIDRES 0.366798 Hz
AQ 1.3631988 sec
RG 196.92
DW 20.800 usec
DE 6.50 usec
TE 298.1 K
D1 2.00000000 sec
D11 0.03000000 sec
TD0 1

======== CHANNEL f1 ========
SFO1 100.6228298 MHz
NUC1 13C
P1 9.70 usec
SI 32768
SF 100.6127787 MHz
WDW EM
SSB 0
LB 1.00 Hz
GB 0
PC 1.40

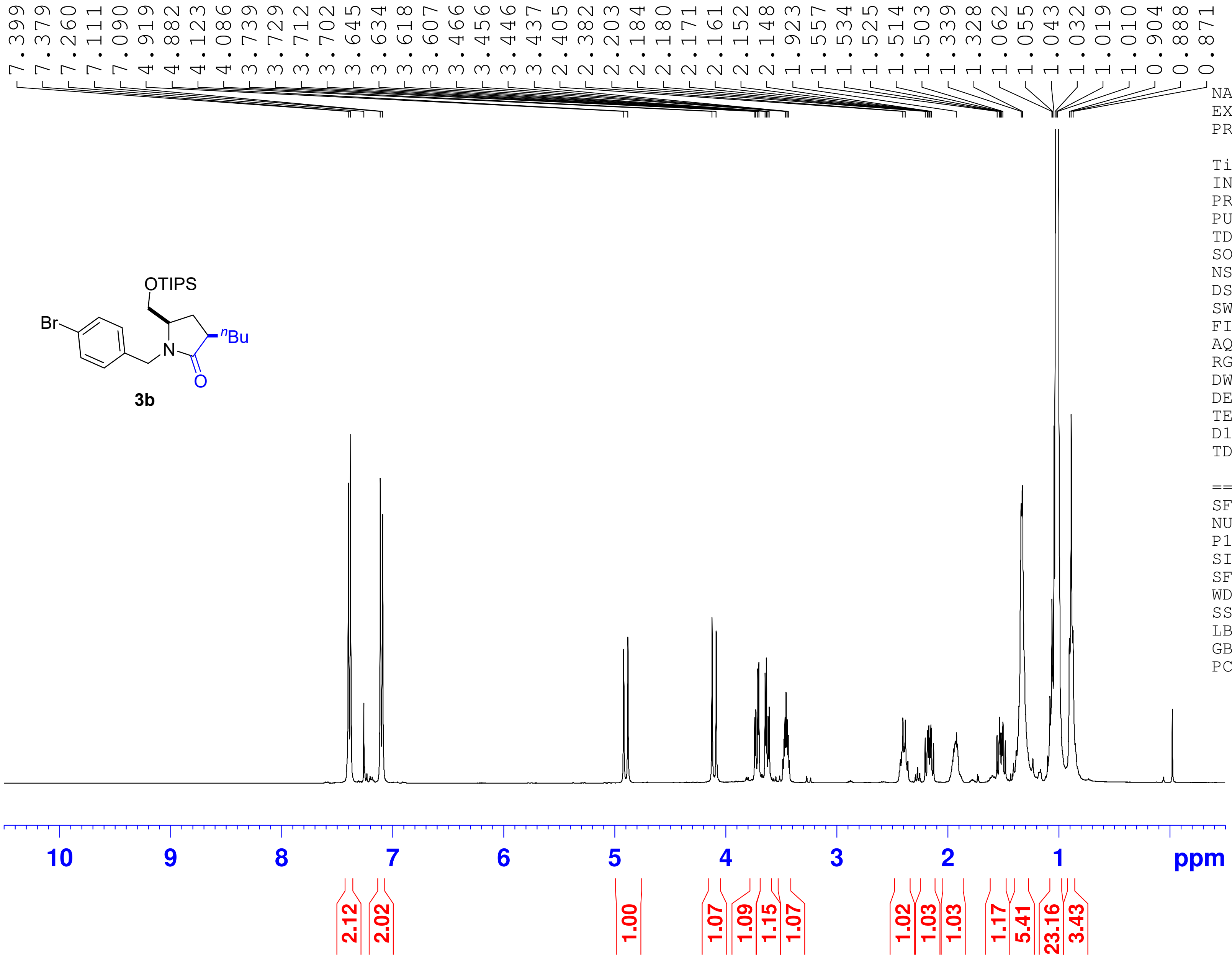

NAME	9-2-2-2
EXPNO	1
PROCNO	1
Time	10.32
INSTRUM	spect
PROBHD	5 mm PABBO BB/
PULPROG	zg30
TD	65536
SOLVENT	CDCl3
NS	8
DS	0
SWH	8012.820 Hz
FIDRES	0.122266 Hz
AQ	4.0894966 sec
RG	19.7
DW	62.400 usec
DE	6.50 usec
TE	297.5 K
D1	1.00000000 sec
TD0	1
======== CHANNEL f1 ========	
SFO1	400.1324710 MHz
NUC1	1H
P1	14.50 usec
SI	65536
SF	400.1300100 MHz
WDW	EM
SSB	0
LB	0.30 Hz
GB	0
PC	1.00

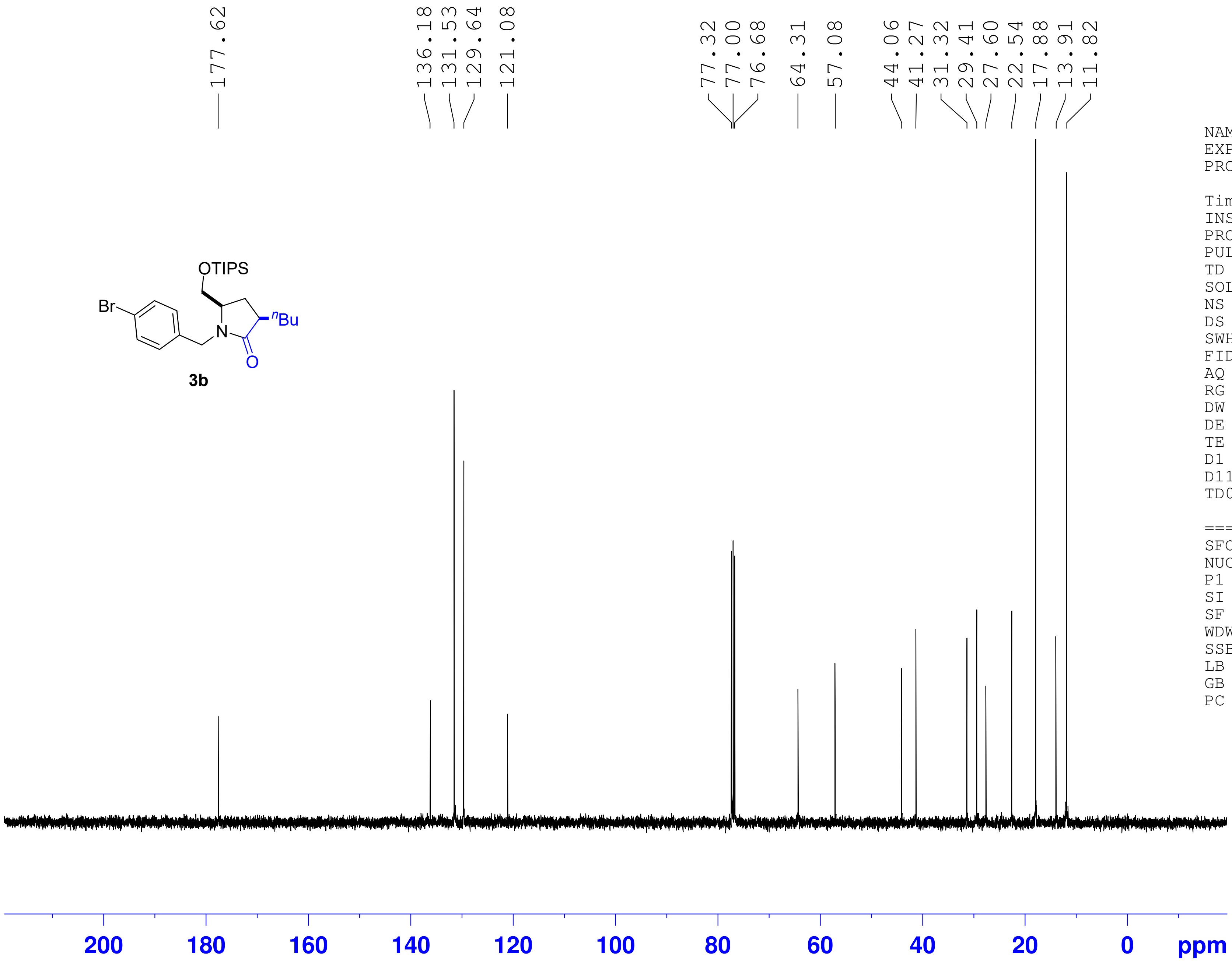


```
NAME                 9-2-2-2
EXPNO                      2
PROCNO                     1

Time                   10.34
INSTRUM                spect
PROBHD      5 mm PABBO BB/
PULPROG              zgpg30
TD                    65536
SOLVENT               CDCl3
NS                        40
DS                         0
SWH              24038.461 Hz
FIDRES            0.366798 Hz
AQ               1.3631988 sec
RG                  196.92
DW                  20.800 usec
DE                    6.50 usec
TE                   298.2 K
D1              2.00000000 sec
D11             0.03000000 sec
TD0                        1

======== CHANNEL f1 ========
SFO1          100.6228298 MHz
NUC1                   13C
P1                    9.70 usec
SI                   32768
SF            100.6127765 MHz
WDW                     EM
SSB                      0
LB                    1.00 Hz
GB                       0
PC                    1.40
```

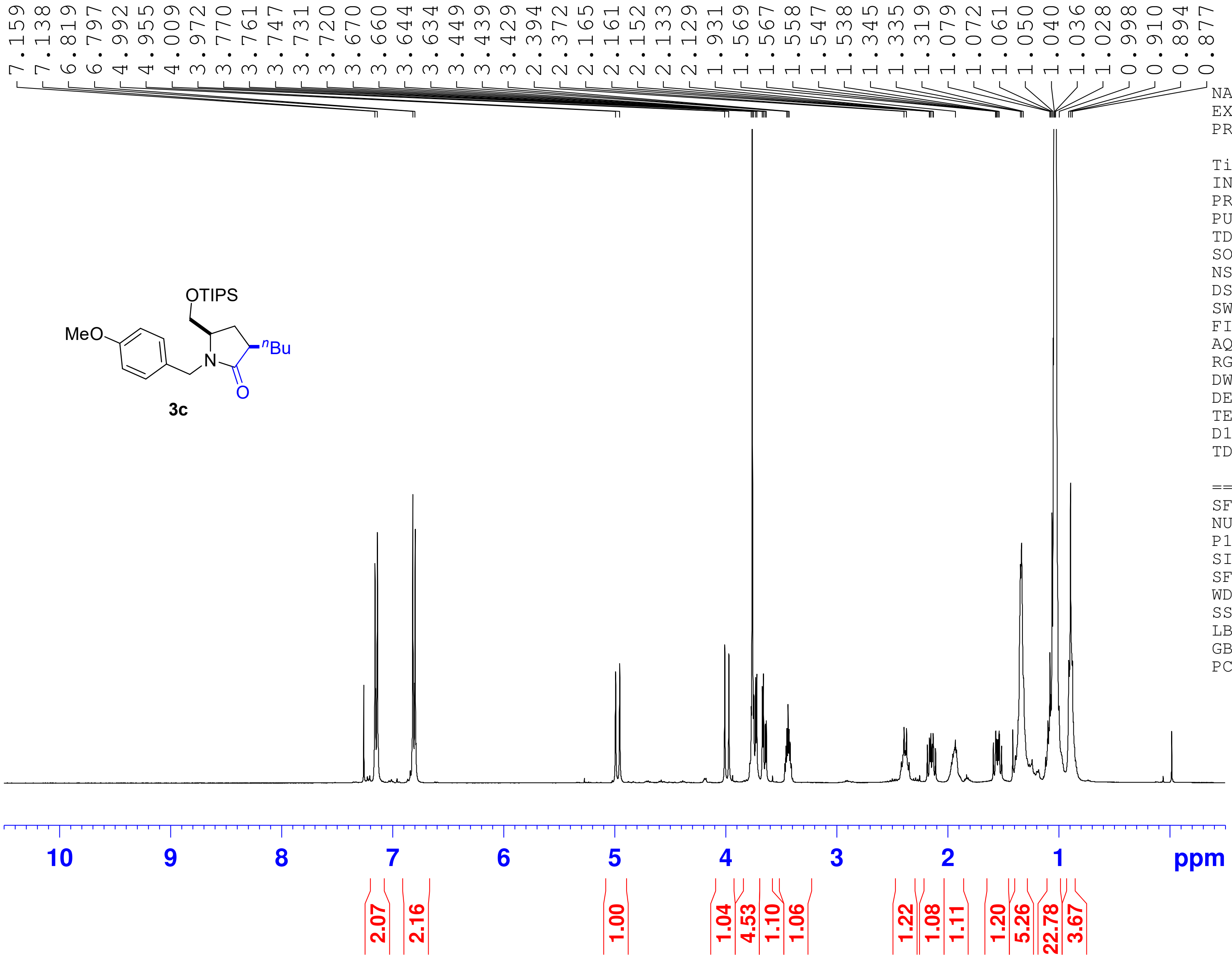

NAME	8-150
EXPNO	1
PROCNO	1
Time	22.20
INSTRUM	spect
PROBHD	5 mm PABBO BB/
PULPROG	zg30
TD	65536
SOLVENT	CDCl3
NS	8
DS	0
SWH	8012.820 Hz
FIDRES	0.122266 Hz
AQ	4.0894966 sec
RG	19.7
DW	62.400 usec
DE	6.50 usec
TE	297.4 K
D1	1.00000000 sec
TD0	1

======== CHANNEL f1 ========

SFO1	400.1324710 MHz
NUC1	1H
P1	14.50 usec
SI	65536
SF	400.1300099 MHz
WDW	EM
SSB	0
LB	0.30 Hz
GB	0
PC	1.00

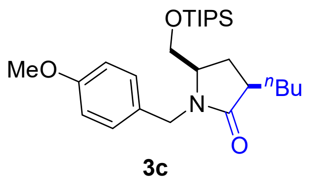

3c

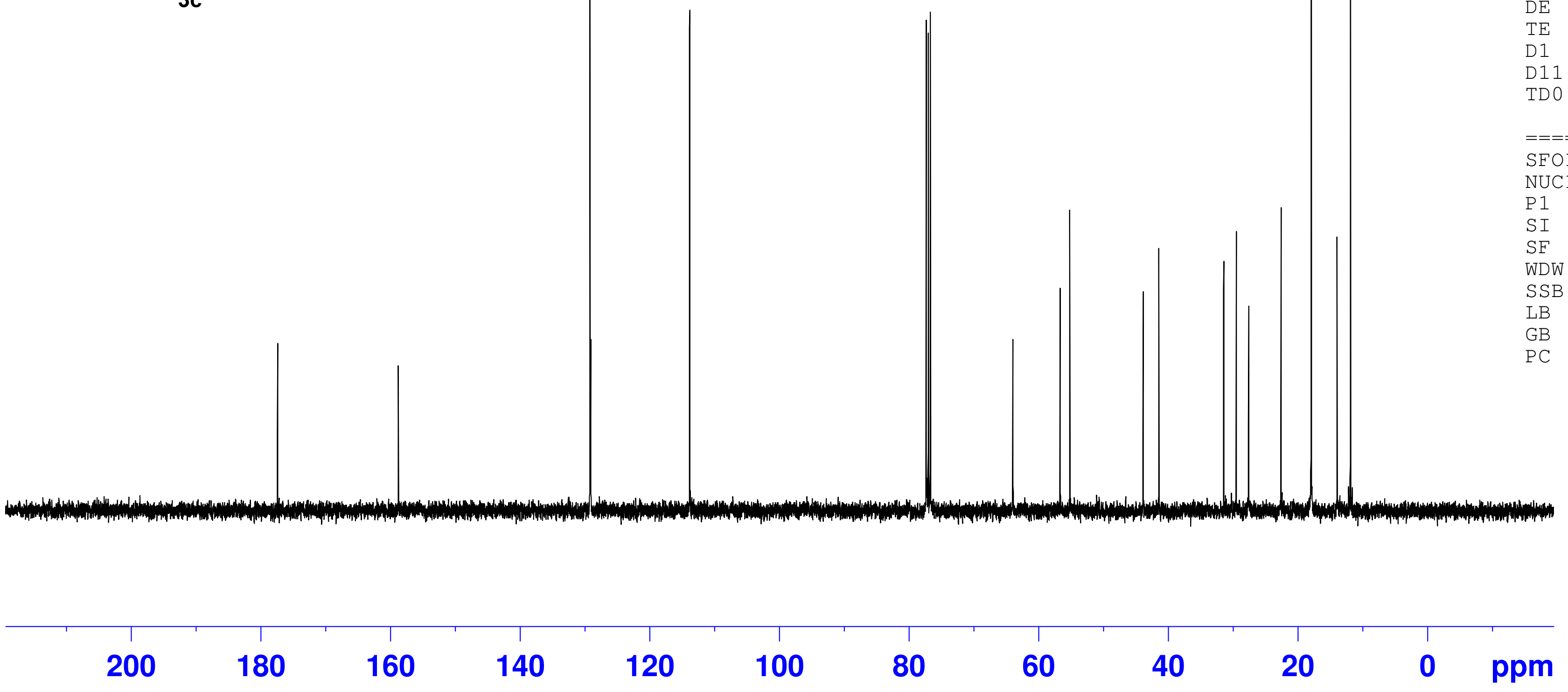

NAME	8-150
EXPNO	2
PROCNO	1
Time	22.22
INSTRUM	spect
PROBHD	5 mm PABBO BB/
PULPROG	zgpg30
TD	65536
SOLVENT	CDCl3
NS	43
DS	0
SWH	24038.461 Hz
FIDRES	0.366798 Hz
AQ	1.3631988 sec
RG	196.92
DW	20.800 usec
DE	6.50 usec
TE	298.1 K
D1	2.00000000 sec
D11	0.03000000 sec
TD0	1

======== CHANNEL f1 ========

SFO1	100.6228298 MHz
NUC1	13C
P1	9.70 usec
SI	32768
SF	100.6127762 MHz
WDW	EM
SSB	0
LB	1.00 Hz
GB	0
PC	1.40

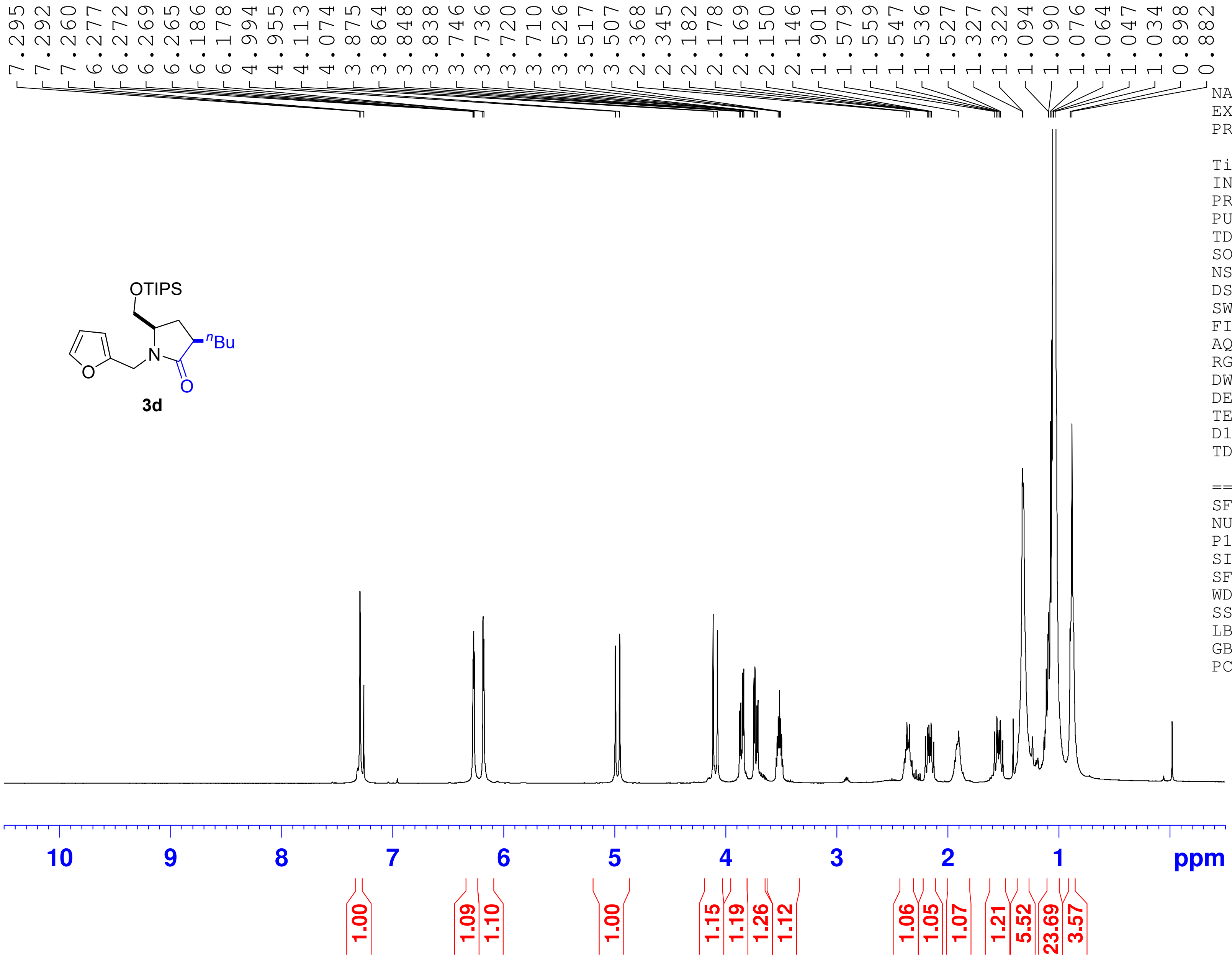

NAME	9-11-2
EXPNO	1
PROCNO	1
Time	10.22
INSTRUM	spect
PROBHD	5 mm PABBO BB/
PULPROG	zg30
TD	65536
SOLVENT	CDCl3
NS	8
DS	0
SWH	8012.820 Hz
FIDRES	0.122266 Hz
AQ	4.0894966 sec
RG	19.7
DW	62.400 usec
DE	6.50 usec
TE	296.8 K
D1	1.00000000 sec
TD0	1

======== CHANNEL f1 ========

SFO1	400.1324710 MHz
NUC1	1H
P1	14.50 usec
SI	65536
SF	400.1300098 MHz
WDW	EM
SSB	0
LB	0.30 Hz
GB	0
PC	1.00

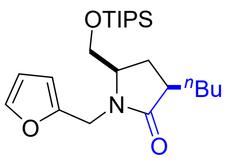

3d

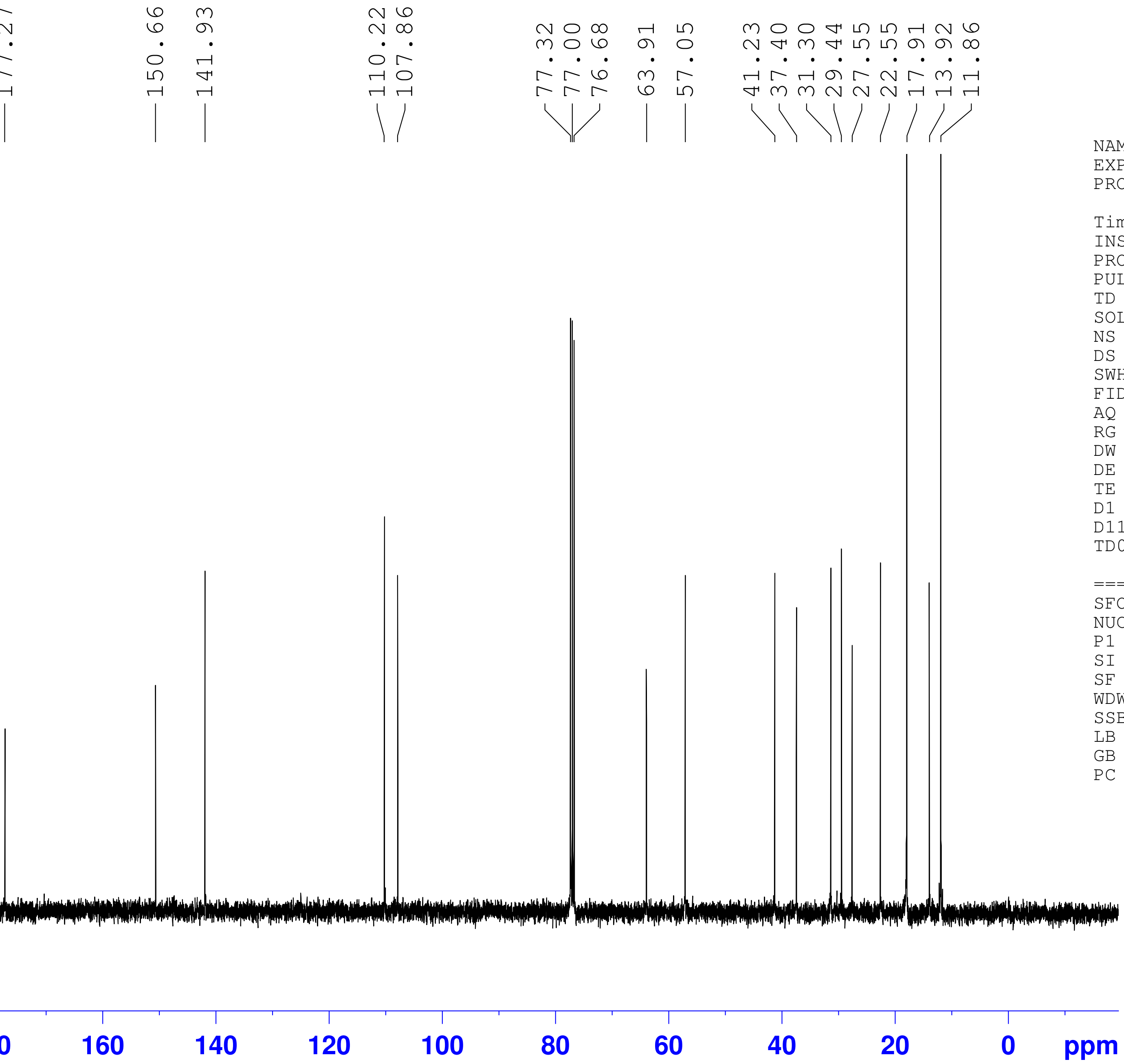

NAME	9-11-2
EXPNO	2
PROCNO	1
Time	10.24
INSTRUM	spect
PROBHD	5 mm PABBO BB/
PULPROG	zgpg30
TD	65536
SOLVENT	CDCl3
NS	48
DS	0
SWH	24038.461 Hz
FIDRES	0.366798 Hz
AQ	1.3631988 sec
RG	196.92
DW	20.800 usec
DE	6.50 usec
TE	297.6 K
D1	2.00000000 sec
D11	0.03000000 sec
TD0	1
======== CHANNEL f1 ========	
SFO1	100.6228298 MHz
NUC1	13C
P1	9.70 usec
SI	32768
SF	100.6127752 MHz
WDW	EM
SSB	0
LB	1.00 Hz
GB	0
PC	1.40

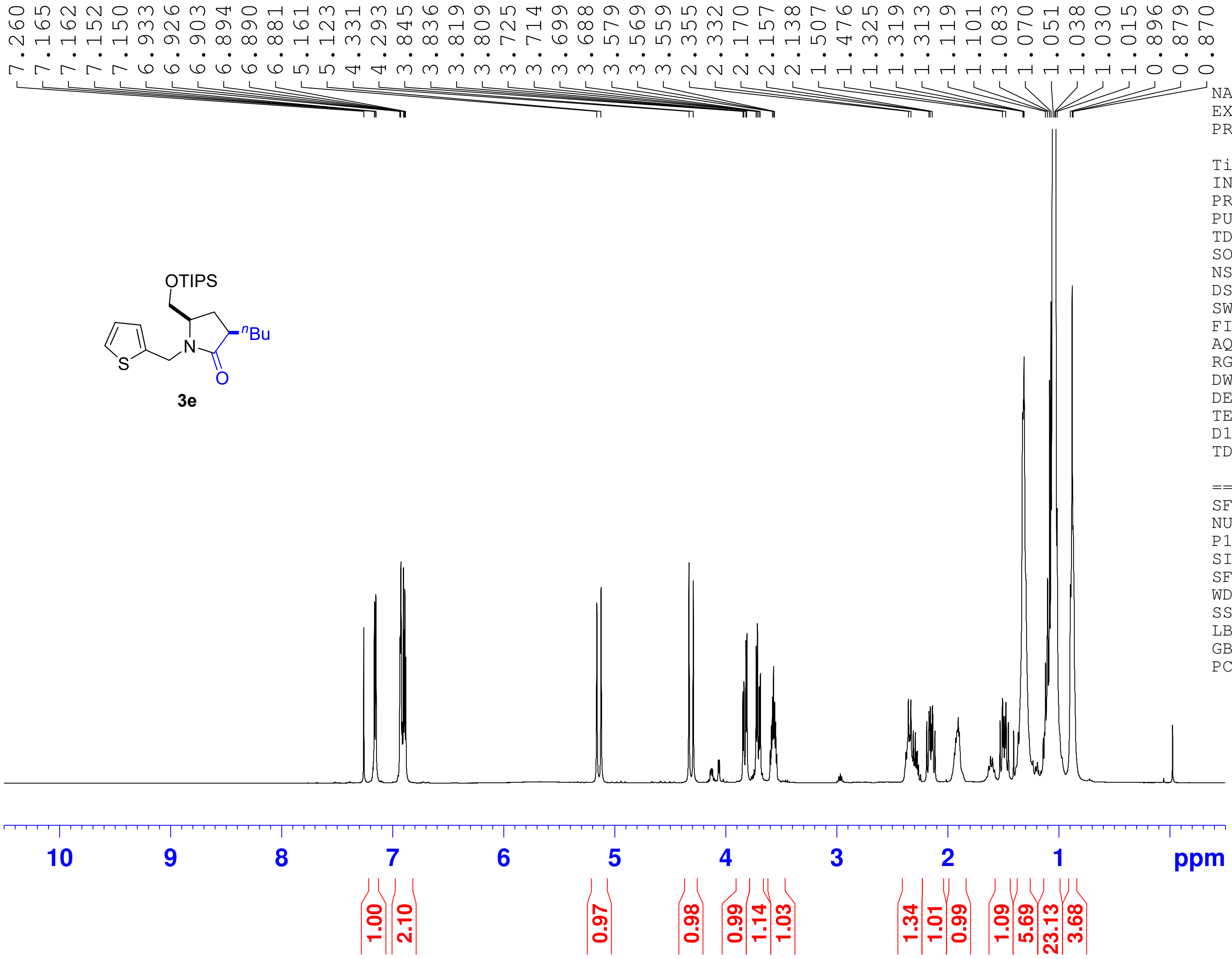

3e

NAME	9-3
EXPNO	1
PROCNO	1
Time	22.22
INSTRUM	spect
PROBHD	5 mm PABBO BB/
PULPROG	zg30
TD	65536
SOLVENT	CDCl3
NS	8
DS	0
SWH	8012.820 Hz
FIDRES	0.122266 Hz
AQ	4.0894966 sec
RG	15.71
DW	62.400 usec
DE	6.50 usec
TE	295.8 K
D1	1.00000000 sec
TD0	1
======== CHANNEL f1 ========	
SFO1	400.1324710 MHz
NUC1	1H
P1	14.50 usec
SI	65536
SF	400.1300098 MHz
WDW	EM
SSB	0
LB	0.30 Hz
GB	0
PC	1.00

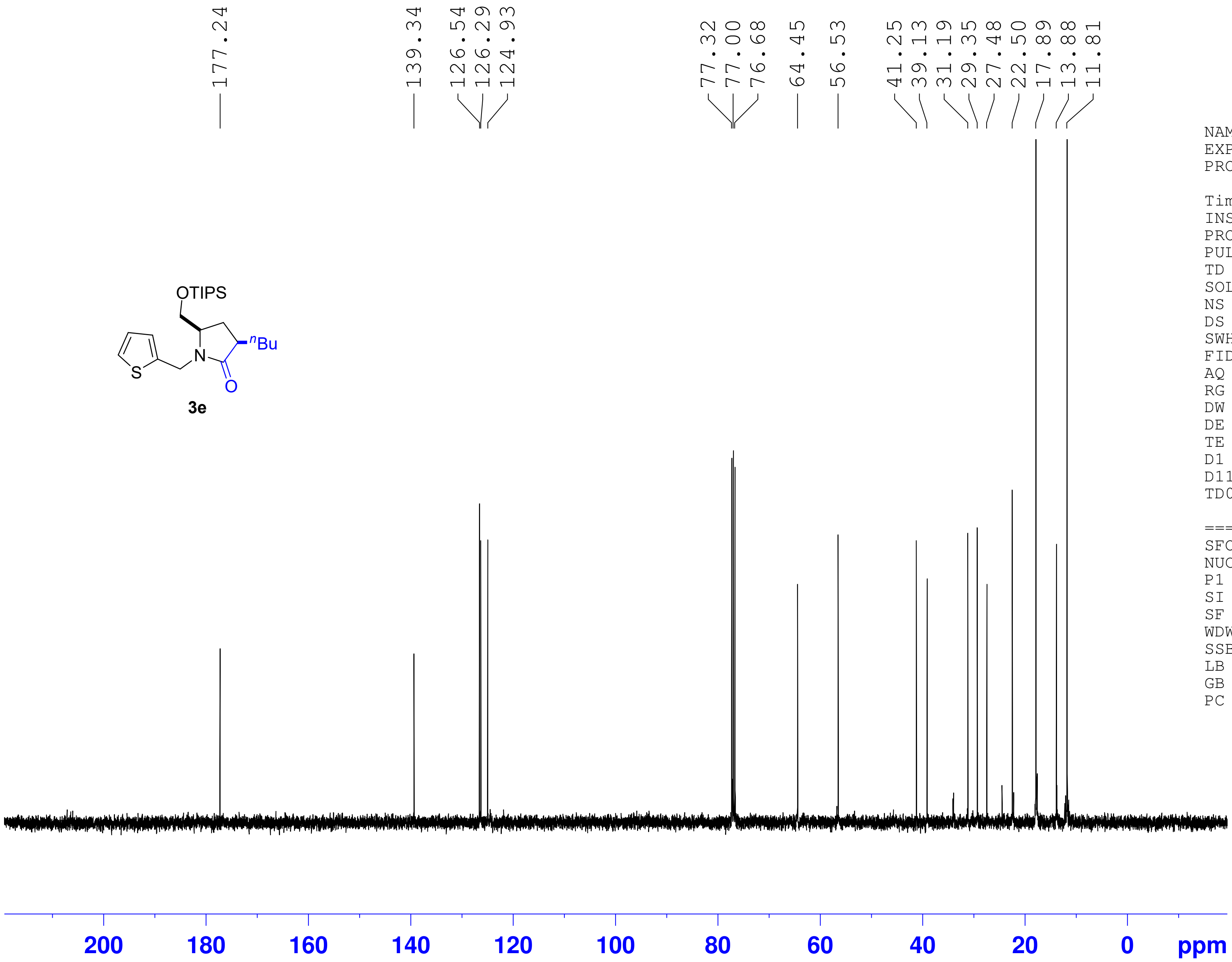

3e

NAME 9-3
EXPNO 2
PROCNO 1

Time 22.24
INSTRUM spect
PROBHD 5 mm PABBO BB/
PULPROG zgpg30
TD 65536
SOLVENT CDCl3
NS 28
DS 0
SWH 24038.461 Hz
FIDRES 0.366798 Hz
AQ 1.3631988 sec
RG 196.92
DW 20.800 usec
DE 6.50 usec
TE 296.3 K
D1 2.00000000 sec
D11 0.03000000 sec
TD0 1

======== CHANNEL f1 ========
SFO1 100.6228298 MHz
NUC1 13C
P1 9.70 usec
SI 32768
SF 100.6127802 MHz
WDW EM
SSB 0
LB 1.00 Hz
GB 0
PC 1.40

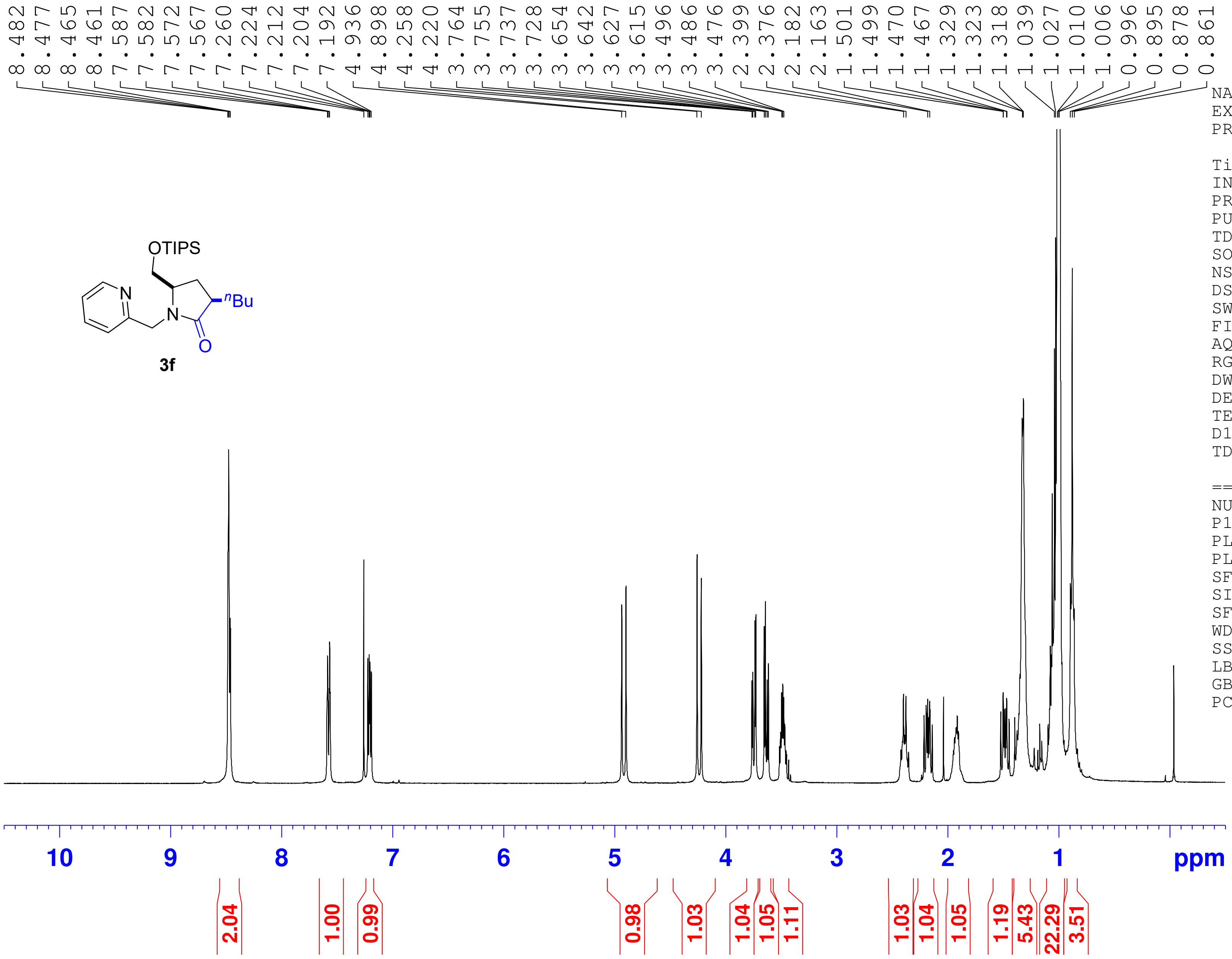

NAME	9-17
EXPNO	1
PROCNO	1
Time	22.16
INSTRUM	spect
PROBHD	5 mm PABBO BB-
PULPROG	zg30
TD	65536
SOLVENT	CDCl3
NS	8
DS	0
SWH	8223.685 Hz
FIDRES	0.125483 Hz
AQ	3.9846387 sec
RG	181
DW	60.800 usec
DE	6.00 usec
TE	295.6 K
D1	1.00000000 sec
TD0	1
======== CHANNEL f1 ========	
NUC1	1H
P1	15.80 usec
PL1	-1.00 dB
PL1W	12.17476940 W
SFO1	400.1324710 MHz
SI	32768
SF	400.1300095 MHz
WDW	EM
SSB	0
LB	0.30 Hz
GB	0
PC	1.00

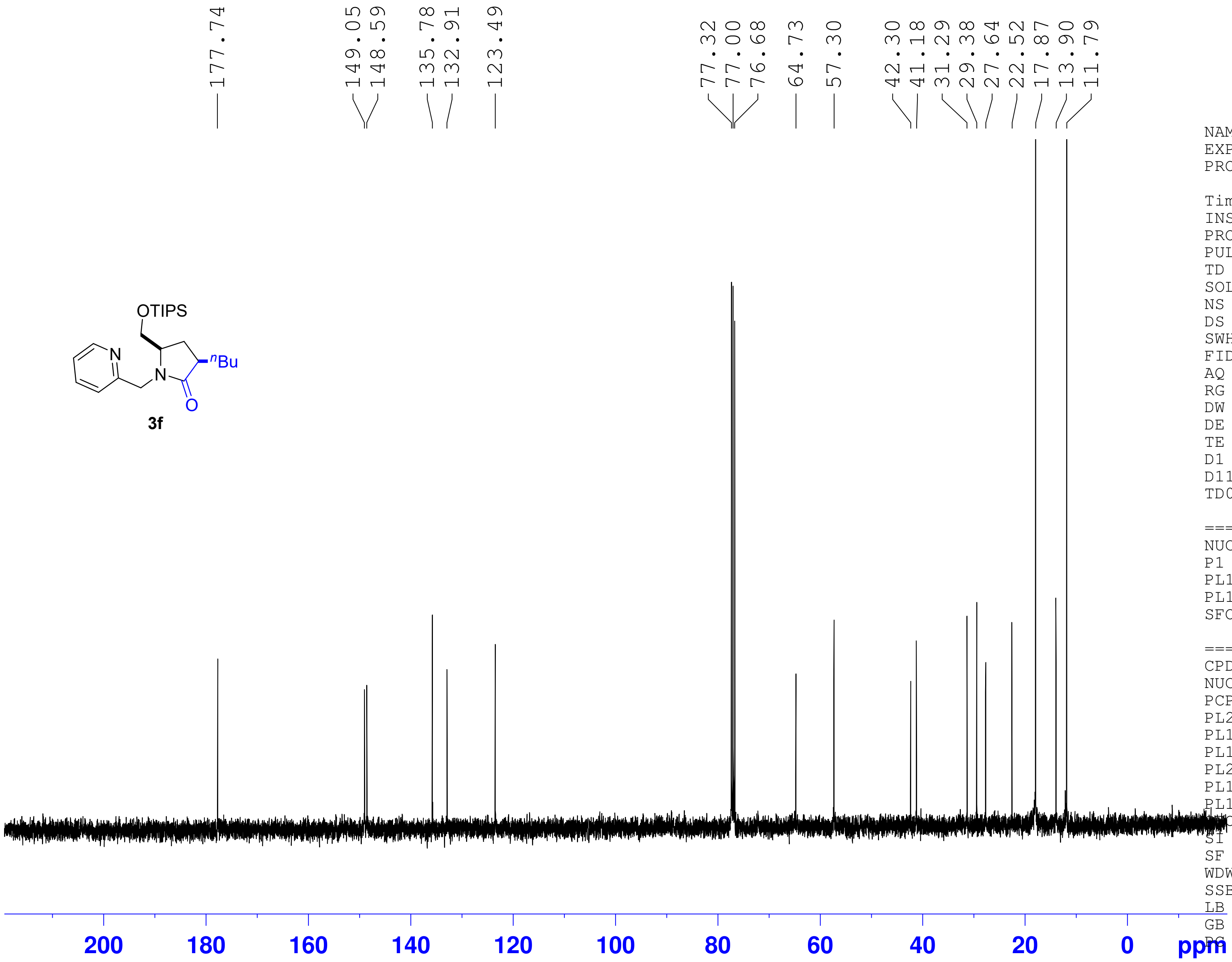

NAME	9-17
EXPNO	2
PROCNO	1
Time	22.19
INSTRUM	spect
PROBHD	5 mm PABBO BB-
PULPROG	zgpg30
TD	65536
SOLVENT	CDCl3
NS	60
DS	0
SWH	24038.461 Hz
FIDRES	0.366798 Hz
AQ	1.3631988 sec
RG	114
DW	20.800 usec
DE	6.00 usec
TE	295.8 K
D1	2.00000000 sec
D11	0.03000000 sec
TD0	1
======== CHANNEL f1 ========	
NUC1	13C
P1	8.60 usec
PL1	-3.00 dB
PL1W	60.64365387 W
SFO1	100.6228298 MHz
======== CHANNEL f2 ========	
CPDPRG2	waltz16
NUC2	1H
PCPD2	80.00 usec
PL2	-1.00 dB
PL12	14.39 dB
PL13	18.00 dB
PL2W	12.17476940 W
PL12W	0.35193357 W
PL13W	0.15327126 W
SFO2	400.1316005 MHz
SI	32768
SF	100.6127758 MHz
WDW	EM
SSB	0
LB	1.00 Hz
GB	0
PC	1.40

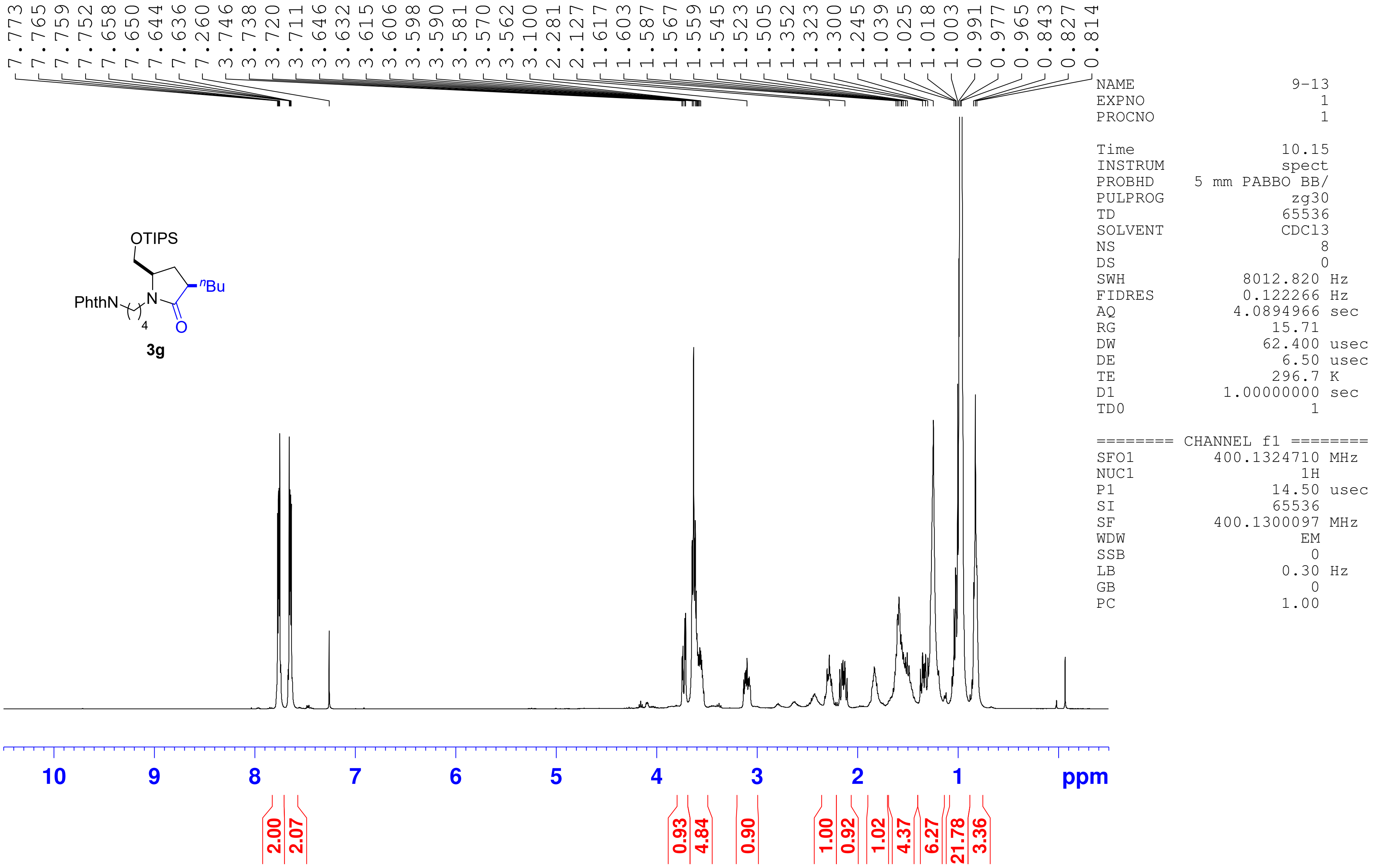
7.773
7.765
7.759
7.752
7.658
7.650
7.644
7.636
7.260
3.746
3.738
3.720
3.711
3.646
3.632
3.615
3.606
3.598
3.590
3.581
3.570
3.562
3.100
2.281
2.127
1.617
1.603
1.587
1.567
1.559
1.545
1.523
1.505
1.352
1.323
1.300
1.245
1.039
1.025
1.018
1.003
0.991
0.977
0.965
0.843
0.827
0.814
NAME 9-13
EXPNO 1
PROCNO 1
Time 10.15
INSTRUM spect
PROBHD 5 mm PABBO BB/
PULPROG zg30
TD 65536
SOLVENT CDCl3
NS 8
DS 0
SWH 8012.820 Hz
FIDRES 0.122266 Hz
AQ 4.0894966 sec
RG 15.71
DW 62.400 usec
DE 6.50 usec
TE 296.7 K
D1 1.00000000 sec
TD0 1
======== CHANNEL f1 ========
SFO1 400.1324710 MHz
NUC1 1H
P1 14.50 usec
SI 65536
SF 400.1300097 MHz
WDW EM
SSB 0
LB 0.30 Hz
GB 0
PC 1.00
OTIPS
PhthN
N
4
O
nBu
3g
10
9
8
7
6
5
4
3
2
1
ppm
2.00
2.07
0.93
4.84
0.90
1.00
0.92
1.02
4.37
6.27
21.78
3.36

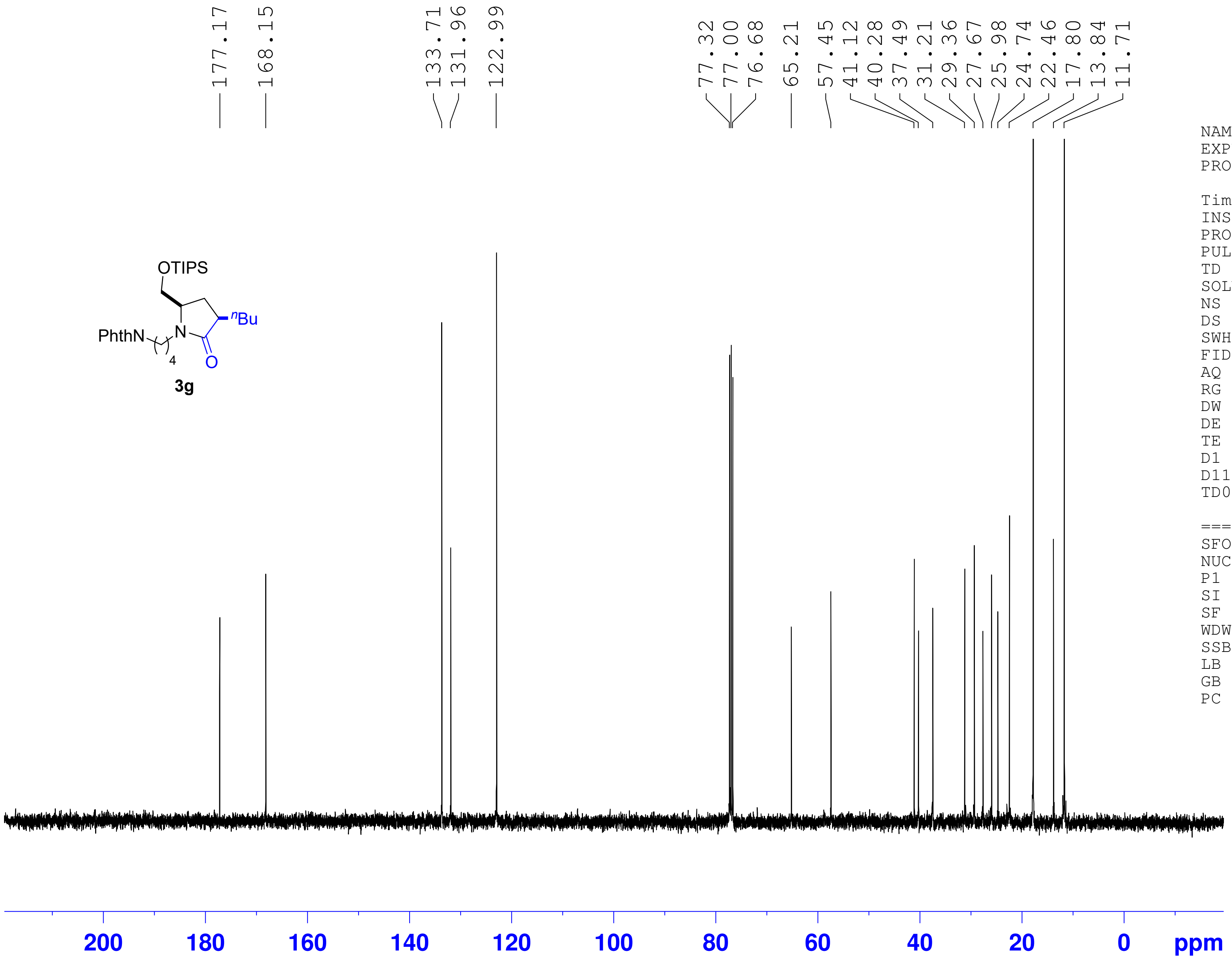

NAME	9-13
EXPNO	2
PROCNO	1
Time	10.18
INSTRUM	spect
PROBHD	5 mm PABBO BB/
PULPROG	zgpg30
TD	65536
SOLVENT	CDCl3
NS	40
DS	0
SWH	24038.461 Hz
FIDRES	0.366798 Hz
AQ	1.3631988 sec
RG	196.92
DW	20.800 usec
DE	6.50 usec
TE	297.4 K
D1	2.00000000 sec
D11	0.03000000 sec
TD0	1

======== CHANNEL f1 ========

SFO1	100.6228298 MHz
NUC1	13C
P1	9.70 usec
SI	32768
SF	100.6127810 MHz
WDW	EM
SSB	0
LB	1.00 Hz
GB	0
PC	1.40

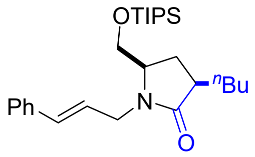

3h

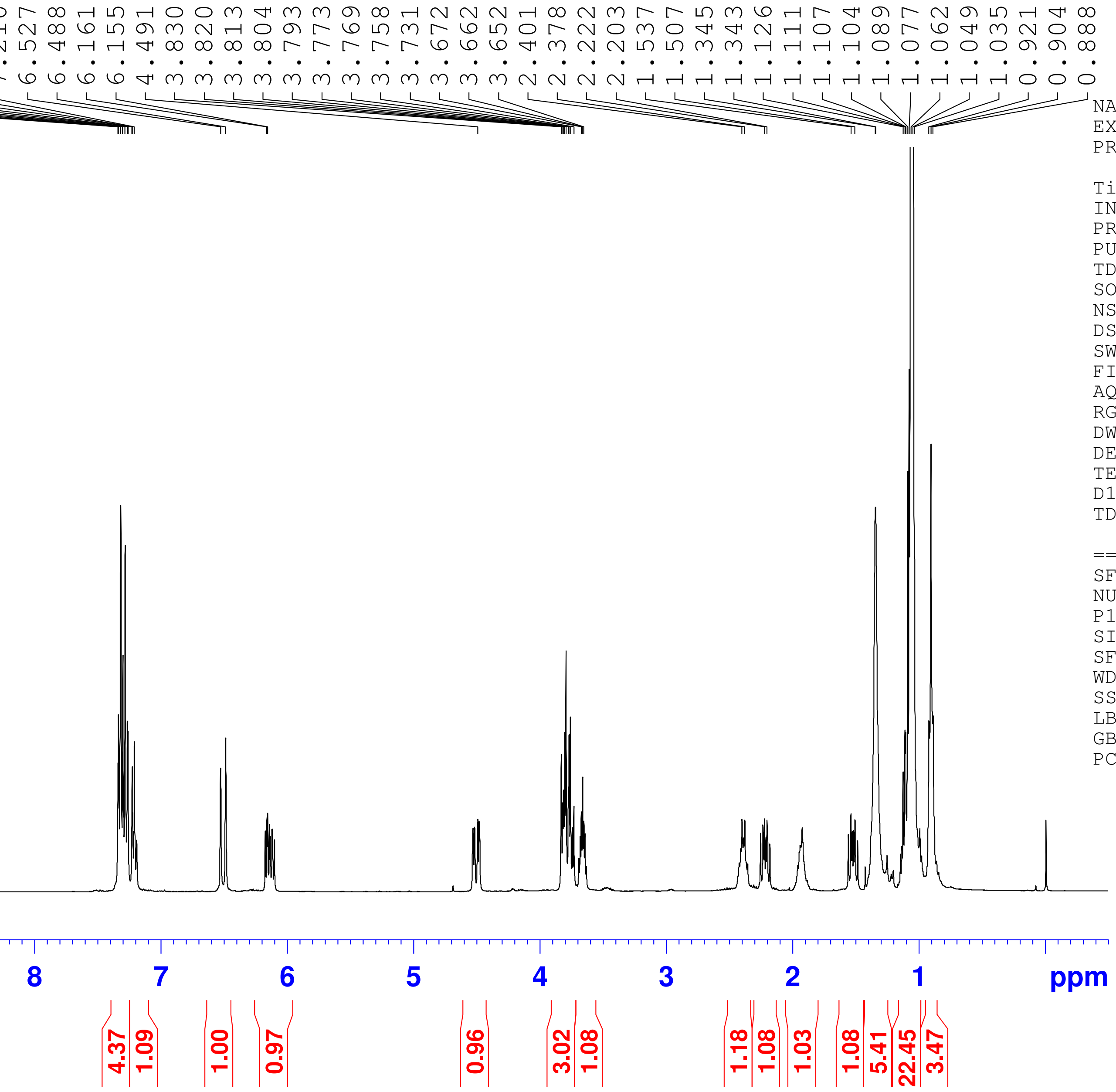

NAME 9-10-2-2
EXPNO 1
PROCNO 1

Time 10.28
INSTRUM spect
PROBHD 5 mm PABBO BB/
PULPROG zg30
TD 65536
SOLVENT CDCl3
NS 8
DS 0
SWH 8012.820 Hz
FIDRES 0.122266 Hz
AQ 4.0894966 sec
RG 17.38
DW 62.400 usec
DE 6.50 usec
TE 296.8 K
D1 1.00000000 sec
TD0 1

======== CHANNEL f1 ========
SFO1 400.1324710 MHz
NUC1 1H
P1 14.50 usec
SI 65536
SF 400.1300100 MHz
WDW EM
SSB 0
LB 0.30 Hz
GB 0
PC 1.00

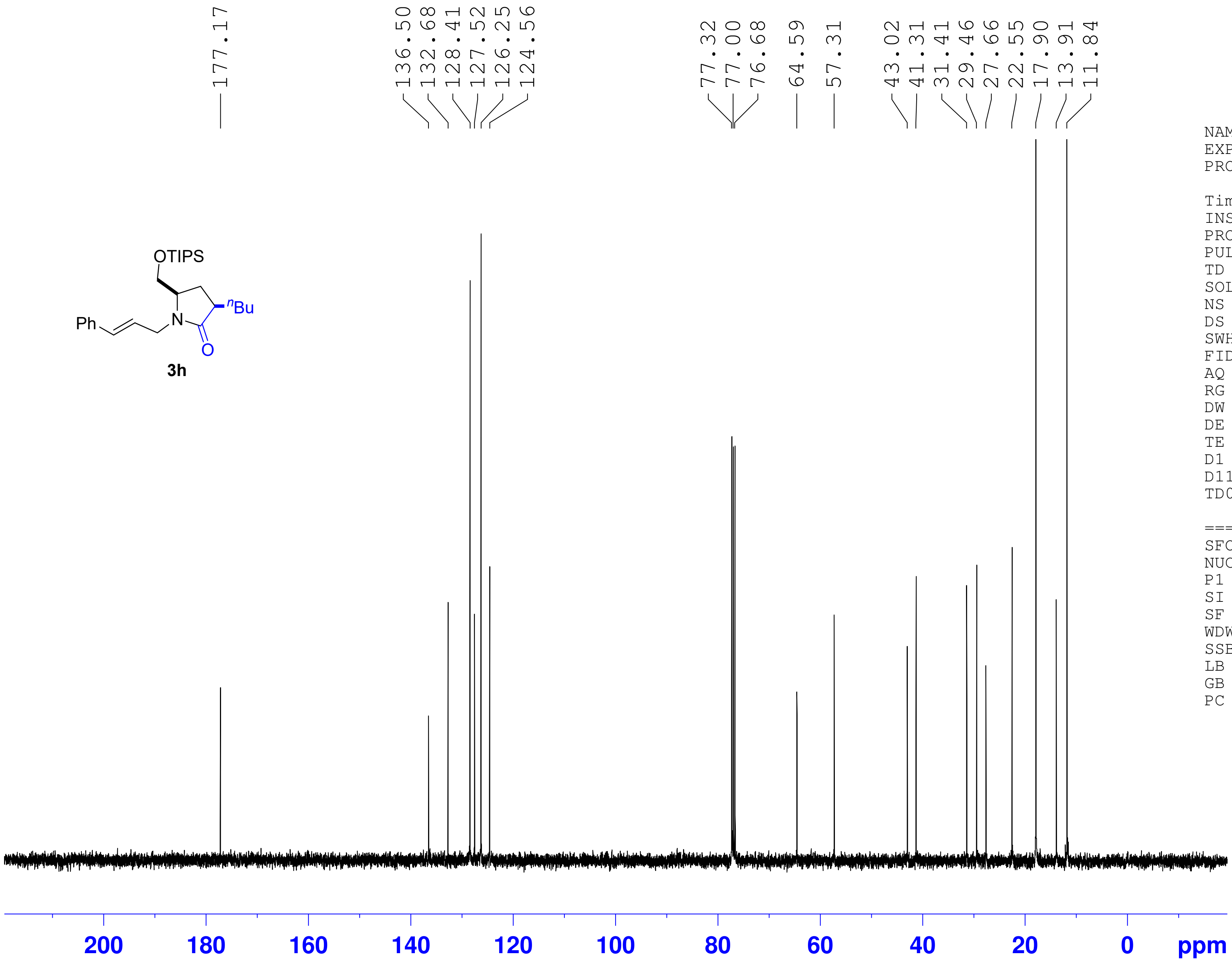

NAME	9-10-2-2
EXPNO	2
PROCNO	1
Time	10.30
INSTRUM	spect
PROBHD	5 mm PABBO BB/
PULPROG	zgpg30
TD	65536
SOLVENT	CDCl3
NS	40
DS	0
SWH	24038.461 Hz
FIDRES	0.366798 Hz
AQ	1.3631988 sec
RG	196.92
DW	20.800 usec
DE	6.50 usec
TE	297.4 K
D1	2.00000000 sec
D11	0.03000000 sec
TD0	1

======== CHANNEL f1 ========

SFO1	100.6228298 MHz
NUC1	13C
P1	9.70 usec
SI	32768
SF	100.6127796 MHz
WDW	EM
SSB	0
LB	1.00 Hz
GB	0
PC	1.40

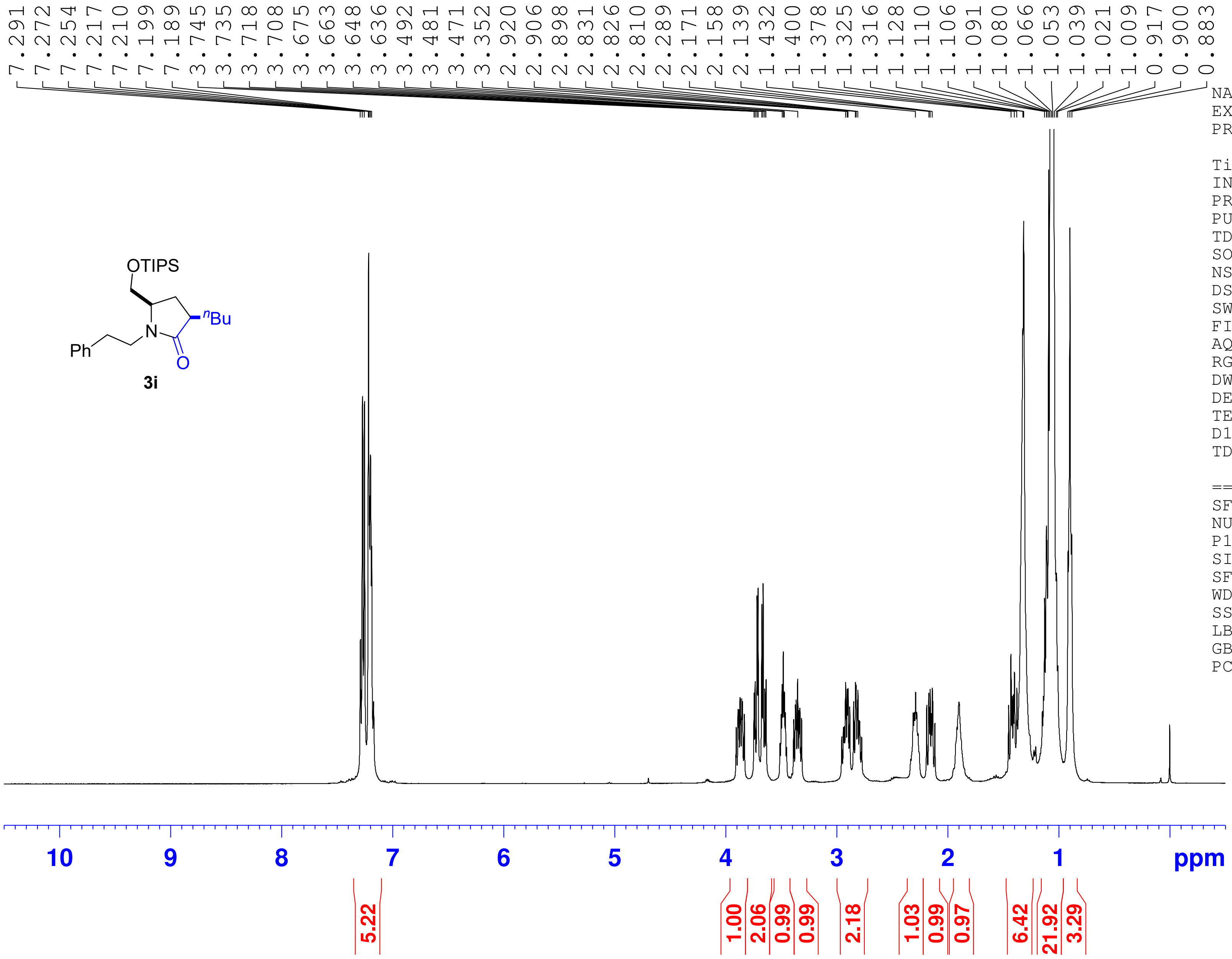

NAME	9-12-2
EXPNO	1
PROCNO	1
Time	17.15
INSTRUM	spect
PROBHD	5 mm PABBO BB/
PULPROG	zg30
TD	65536
SOLVENT	CDCl3
NS	8
DS	0
SWH	8012.820 Hz
FIDRES	0.122266 Hz
AQ	4.0894966 sec
RG	15.71
DW	62.400 usec
DE	6.50 usec
TE	296.9 K
D1	1.00000000 sec
TD0	1

======== CHANNEL f1 ========

SFO1	400.1324710 MHz
NUC1	1H
P1	14.50 usec
SI	65536
SF	400.1300063 MHz
WDW	EM
SSB	0
LB	0.30 Hz
GB	0
PC	1.00

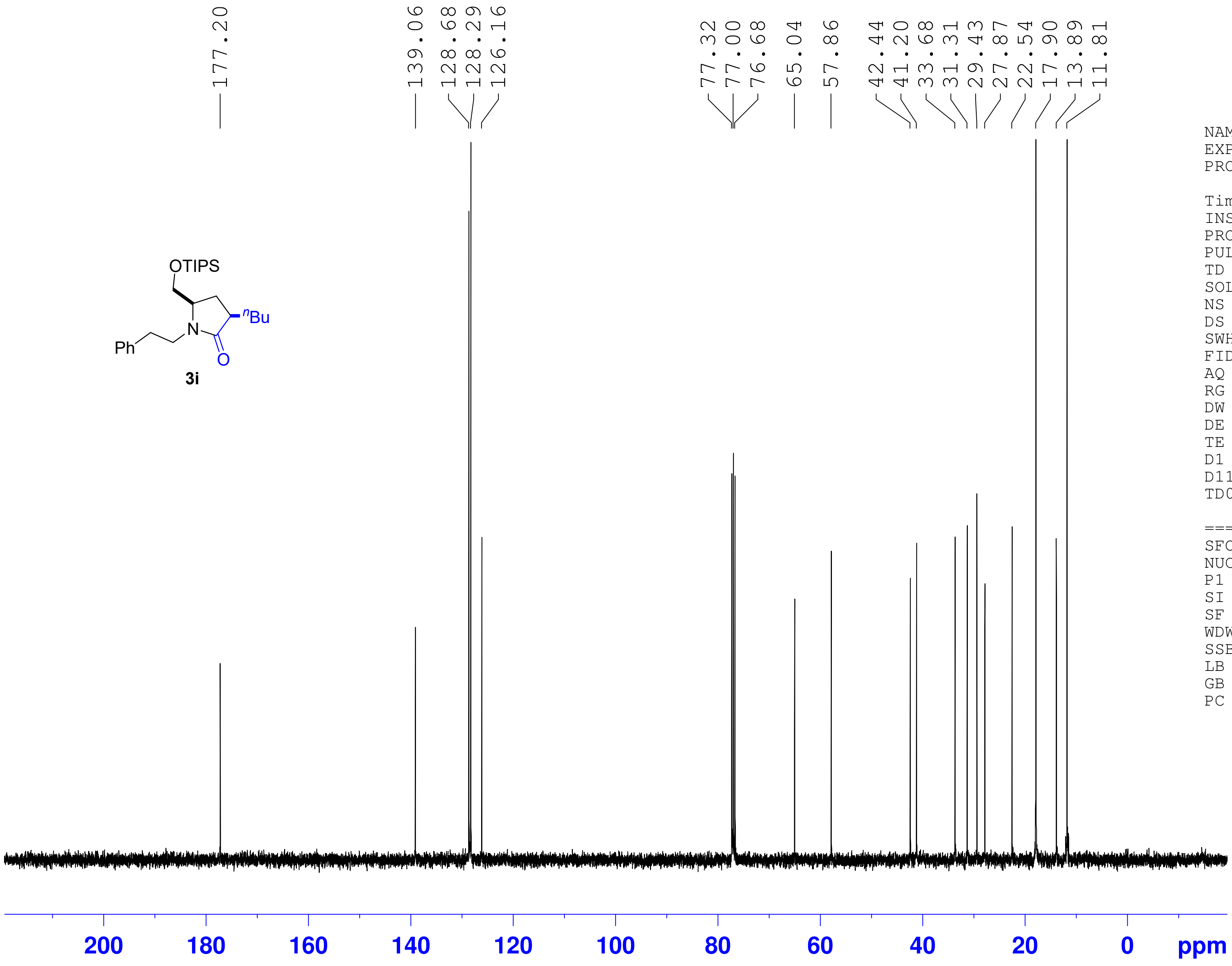

NAME	9-12-2
EXPNO	2
PROCNO	1
Time	17.17
INSTRUM	spect
PROBHD	5 mm PABBO BB/
PULPROG	zgpg30
TD	65536
SOLVENT	CDCl3
NS	40
DS	0
SWH	24038.461 Hz
FIDRES	0.366798 Hz
AQ	1.3631988 sec
RG	196.92
DW	20.800 usec
DE	6.50 usec
TE	297.6 K
D1	2.00000000 sec
D11	0.03000000 sec
TD0	1

======== CHANNEL f1 ========

SFO1	100.6228298 MHz
NUC1	13C
P1	9.70 usec
SI	32768
SF	100.6127802 MHz
WDW	EM
SSB	0
LB	1.00 Hz
GB	0
PC	1.40

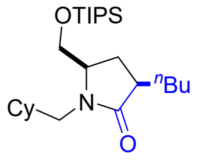

3j

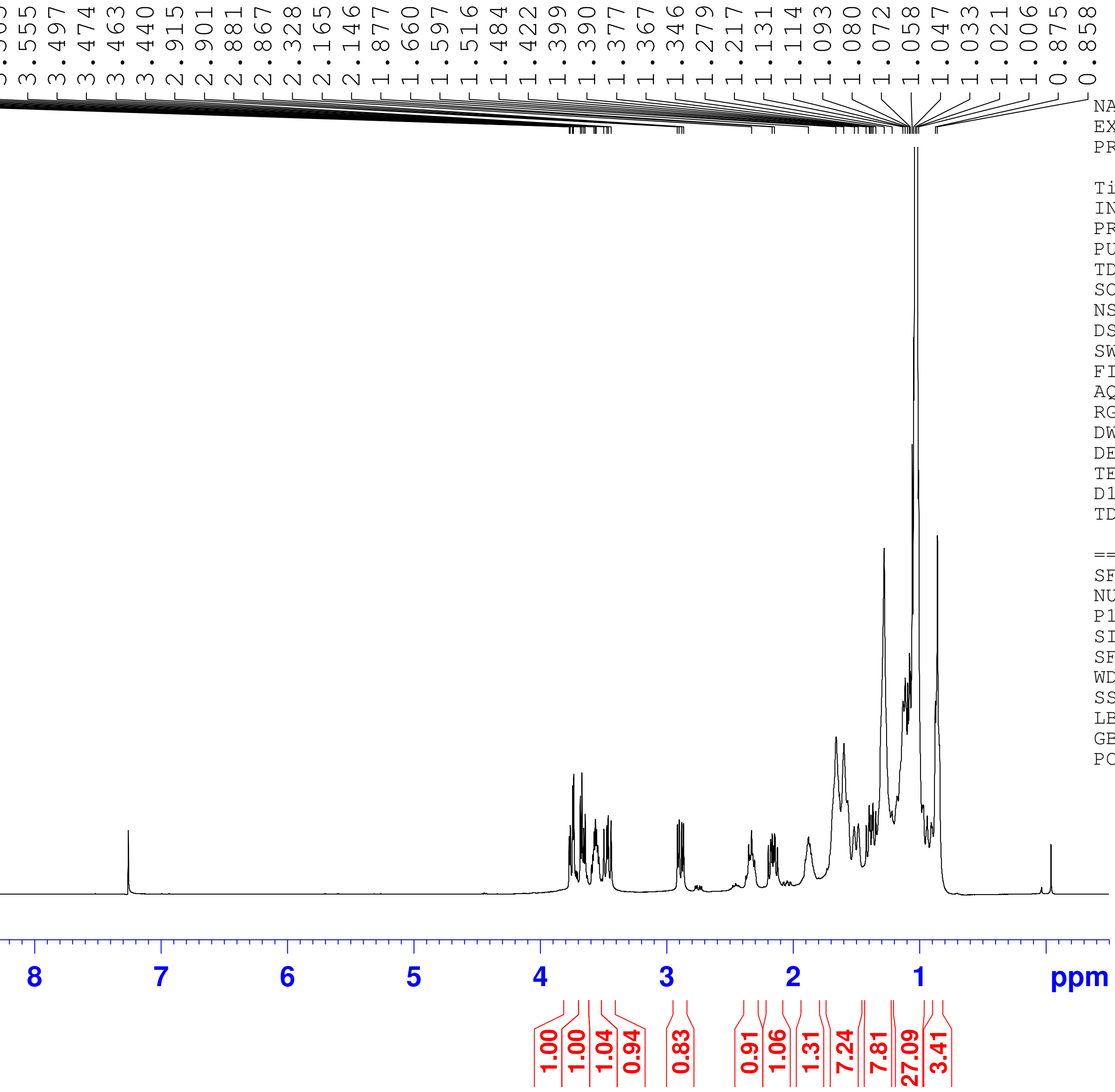

NAME	9-14
EXPNO	1
PROCNO	1
Time	17.08
INSTRUM	spect
PROBHD	5 mm PABBO BB/
PULPROG	zg30
TD	65536
SOLVENT	CDCl3
NS	8
DS	0
SWH	8012.820 Hz
FIDRES	0.122266 Hz
AQ	4.0894966 sec
RG	15.71
DW	62.400 usec
DE	6.50 usec
TE	297.2 K
D1	1.00000000 sec
TD0	1

======== CHANNEL f1 ========

SFO1	400.1324710 MHz
NUC1	1H
P1	14.50 usec
SI	65536
SF	400.1300100 MHz
WDW	EM
SSB	0
LB	0.30 Hz
GB	0
PC	1.00

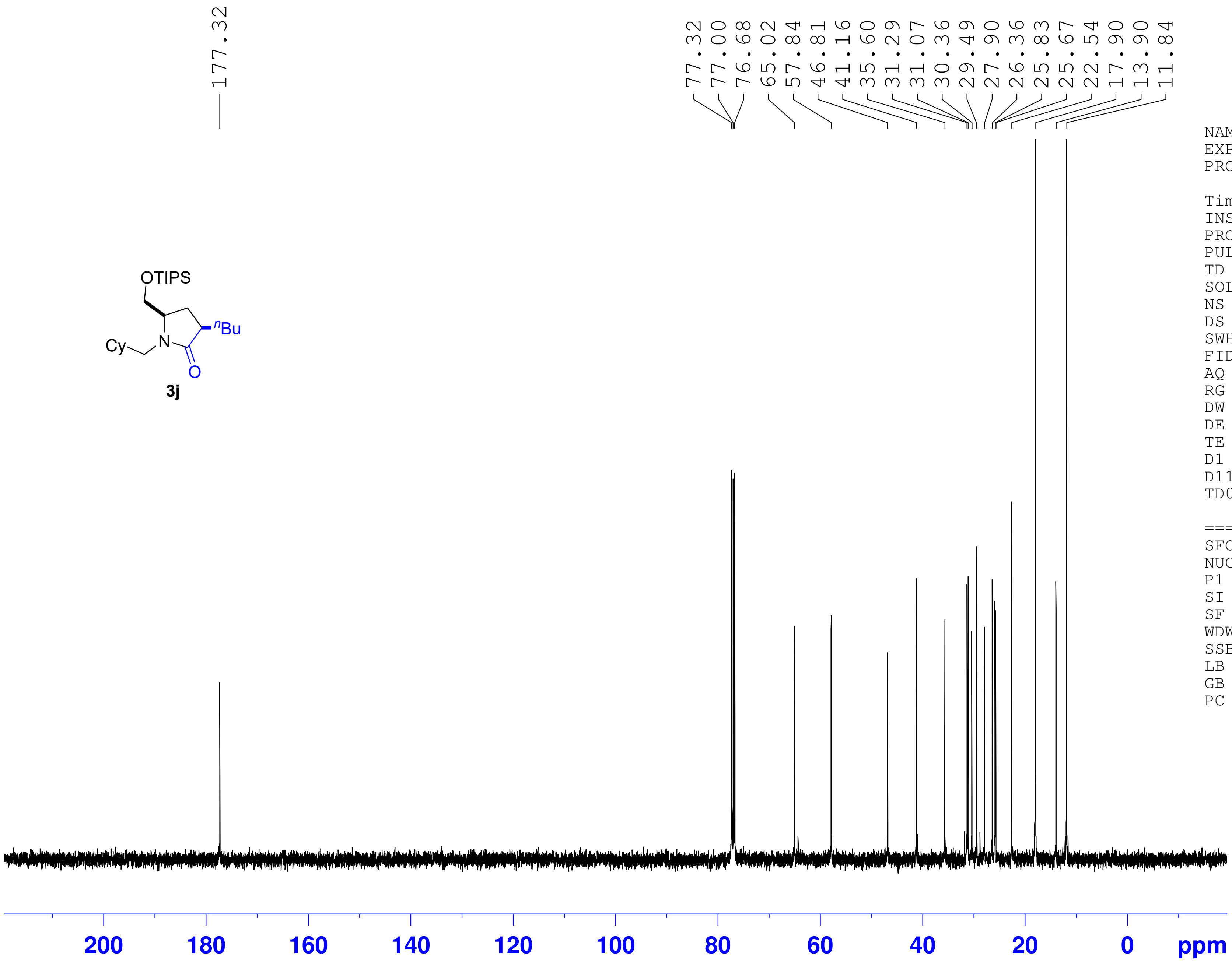

NAME	9-14
EXPNO	2
PROCNO	1
Time	17.10
INSTRUM	spect
PROBHD	5 mm PABBO BB/
PULPROG	zgpg30
TD	65536
SOLVENT	CDCl3
NS	48
DS	0
SWH	24038.461 Hz
FIDRES	0.366798 Hz
AQ	1.3631988 sec
RG	196.92
DW	20.800 usec
DE	6.50 usec
TE	297.8 K
D1	2.00000000 sec
D11	0.03000000 sec
TD0	1
======== CHANNEL f1 ========	
SFO1	100.6228298 MHz
NUC1	13C
P1	9.70 usec
SI	32768
SF	100.6127755 MHz
WDW	EM
SSB	0
LB	1.00 Hz
GB	0
PC	1.40

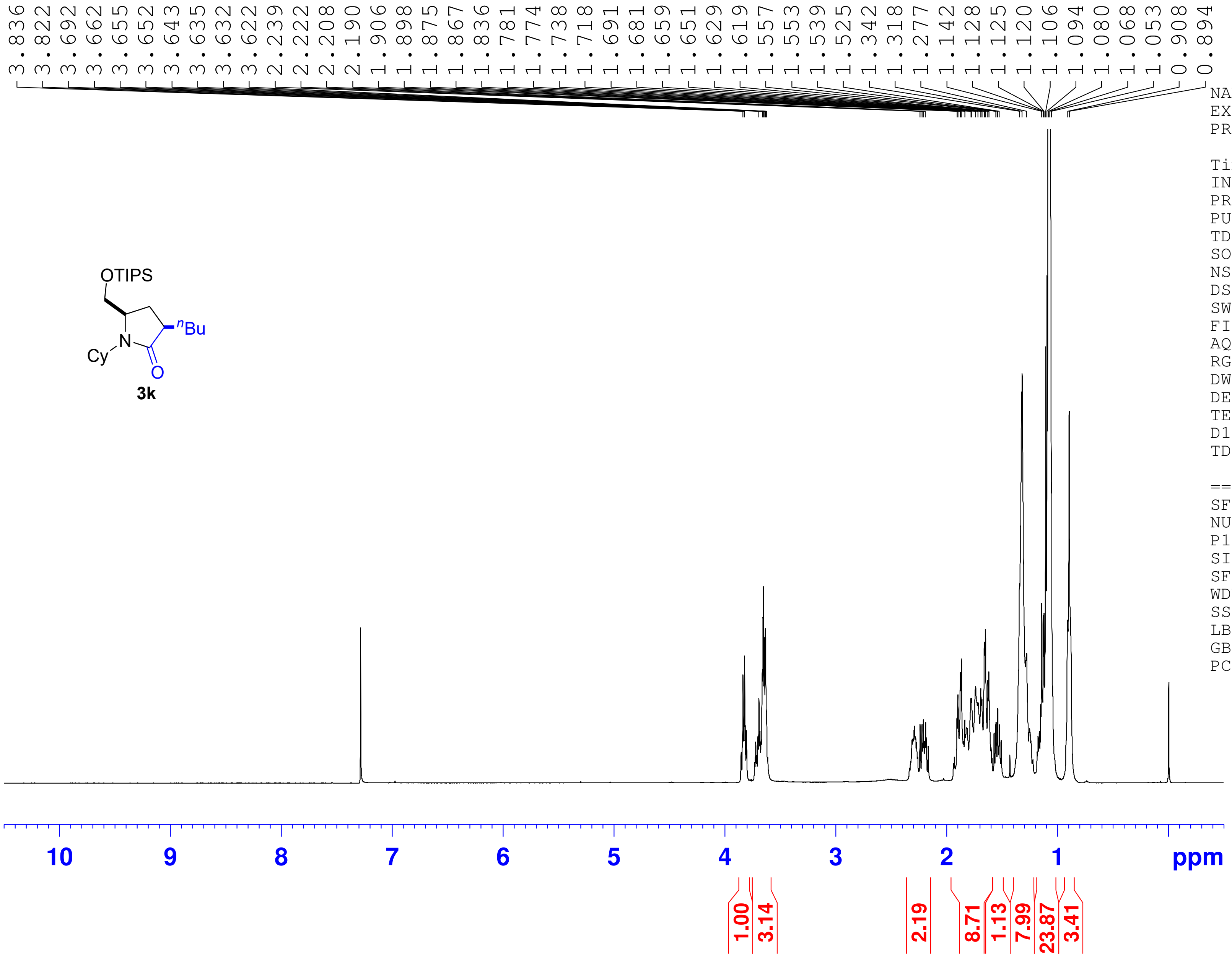

NAME	8-157-2
EXPNO	1
PROCNO	1
Time	21.34
INSTRUM	spect
PROBHD	5 mm PABBO BB/
PULPROG	zg30
TD	65536
SOLVENT	CDCl3
NS	8
DS	0
SWH	8012.820 Hz
FIDRES	0.122266 Hz
AQ	4.0894966 sec
RG	19.7
DW	62.400 usec
DE	6.50 usec
TE	297.2 K
D1	1.00000000 sec
TD0	1
======== CHANNEL f1 ========	
SFO1	400.1324710 MHz
NUC1	1H
P1	14.50 usec
SI	65536
SF	400.1300000 MHz
WDW	EM
SSB	0
LB	0.30 Hz
GB	0
PC	1.00

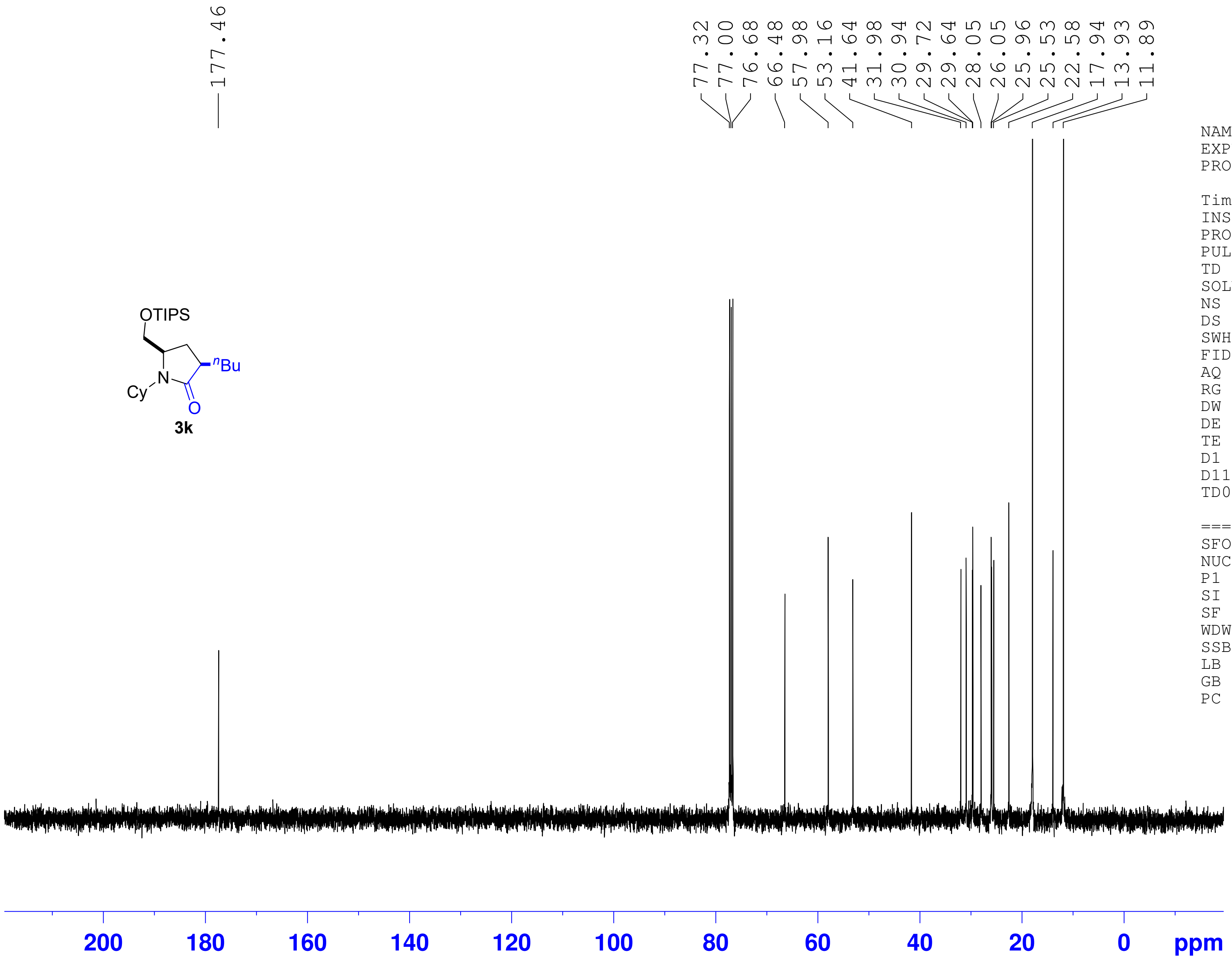

NAME	8-157-2
EXPNO	2
PROCNO	1
Time	21.17
INSTRUM	spect
PROBHD	5 mm PABBO BB/
PULPROG	zgpg30
TD	65536
SOLVENT	CDCl3
NS	40
DS	0
SWH	24038.461 Hz
FIDRES	0.366798 Hz
AQ	1.3631988 sec
RG	196.92
DW	20.800 usec
DE	6.50 usec
TE	298.4 K
D1	2.00000000 sec
D11	0.03000000 sec
TD0	1

======== CHANNEL f1 ========

SFO1	100.6228298 MHz
NUC1	13C
P1	9.70 usec
SI	32768
SF	100.6127738 MHz
WDW	EM
SSB	0
LB	1.00 Hz
GB	0
PC	1.40

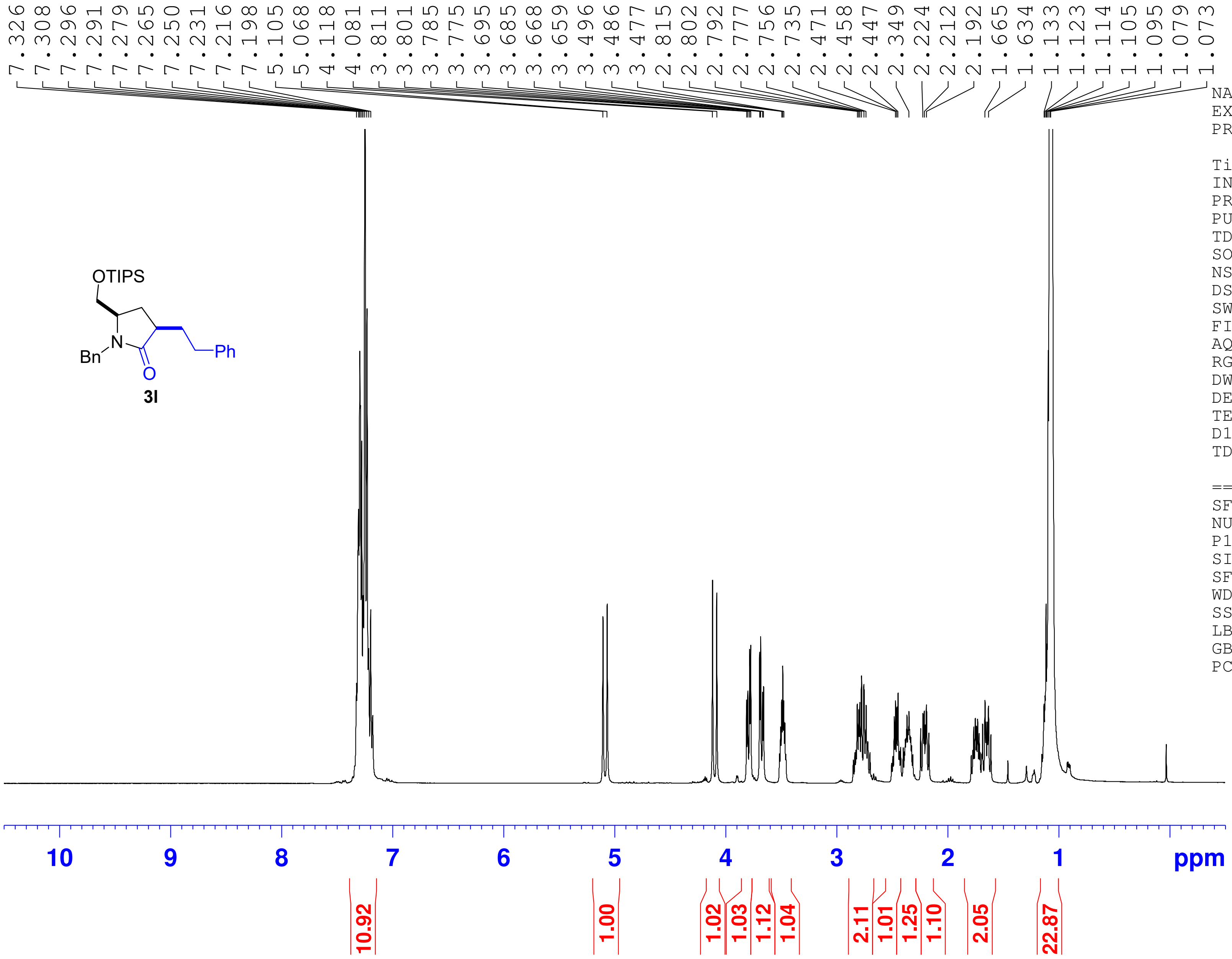

NAME	9-24
EXPNO	1
PROCNO	1
Time	11.11
INSTRUM	spect
PROBHD	5 mm PABBO BB/
PULPROG	zg30
TD	65536
SOLVENT	CDCl3
NS	8
DS	0
SWH	8012.820 Hz
FIDRES	0.122266 Hz
AQ	4.0894966 sec
RG	15.71
DW	62.400 usec
DE	6.50 usec
TE	296.6 K
D1	1.00000000 sec
TD0	1

======== CHANNEL f1 ========

SFO1	400.1324710 MHz
NUC1	1H
P1	14.50 usec
SI	65536
SF	400.1300102 MHz
WDW	EM
SSB	0
LB	0.30 Hz
GB	0
PC	1.00

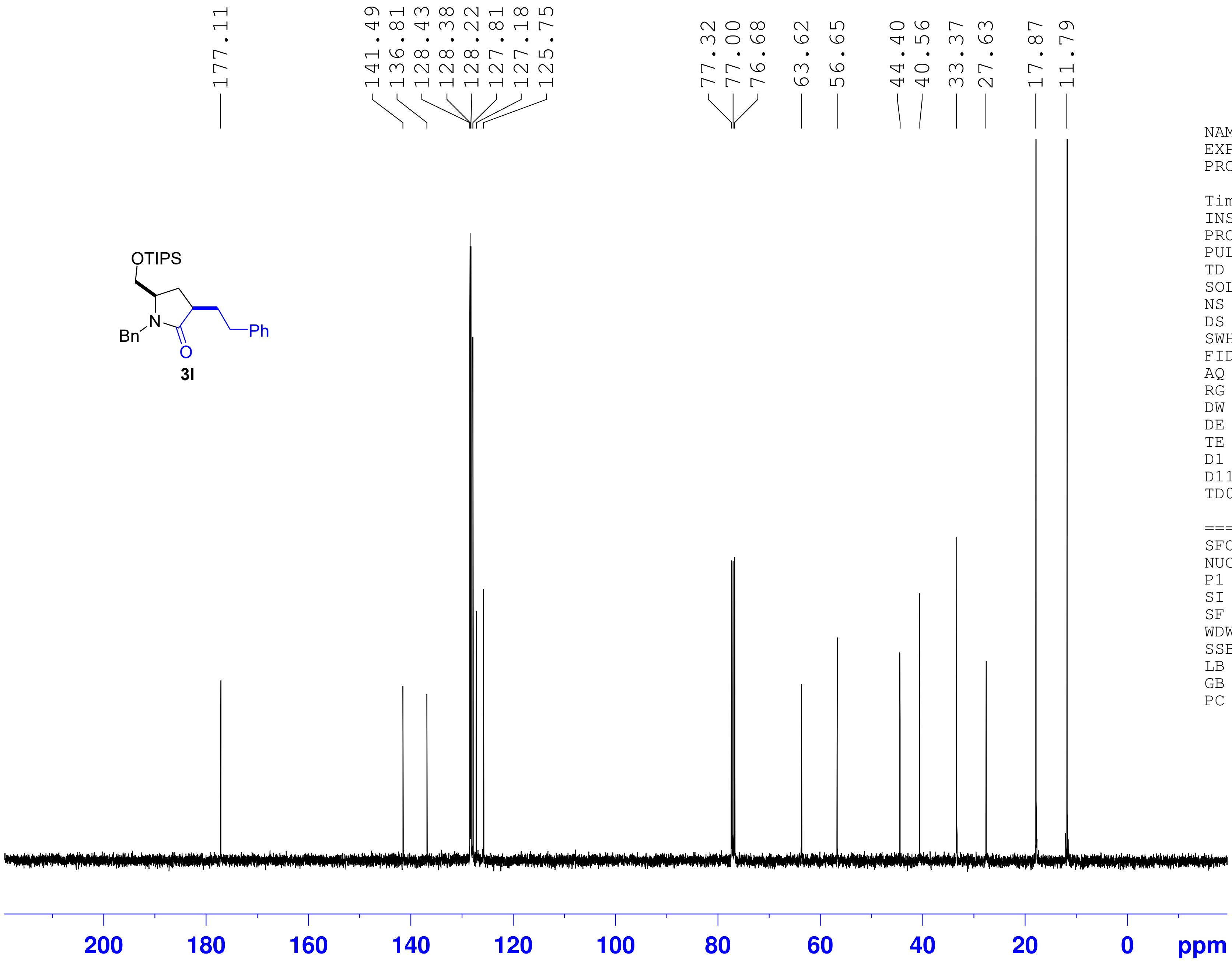

NAME 9-24
EXPNO 2
PROCNO 1

Time 11.13
INSTRUM spect
PROBHD 5 mm PABBO BB/
PULPROG zgpg30
TD 65536
SOLVENT CDCl3
NS 32
DS 0
SWH 24038.461 Hz
FIDRES 0.366798 Hz
AQ 1.3631988 sec
RG 196.92
DW 20.800 usec
DE 6.50 usec
TE 297.2 K
D1 2.00000000 sec
D11 0.03000000 sec
TD0 1

======== CHANNEL f1 ========
SFO1 100.6228298 MHz
NUC1 13C
P1 9.70 usec
SI 32768
SF 100.6127864 MHz
WDW EM
SSB 0
LB 1.00 Hz
GB 0
PC 1.40

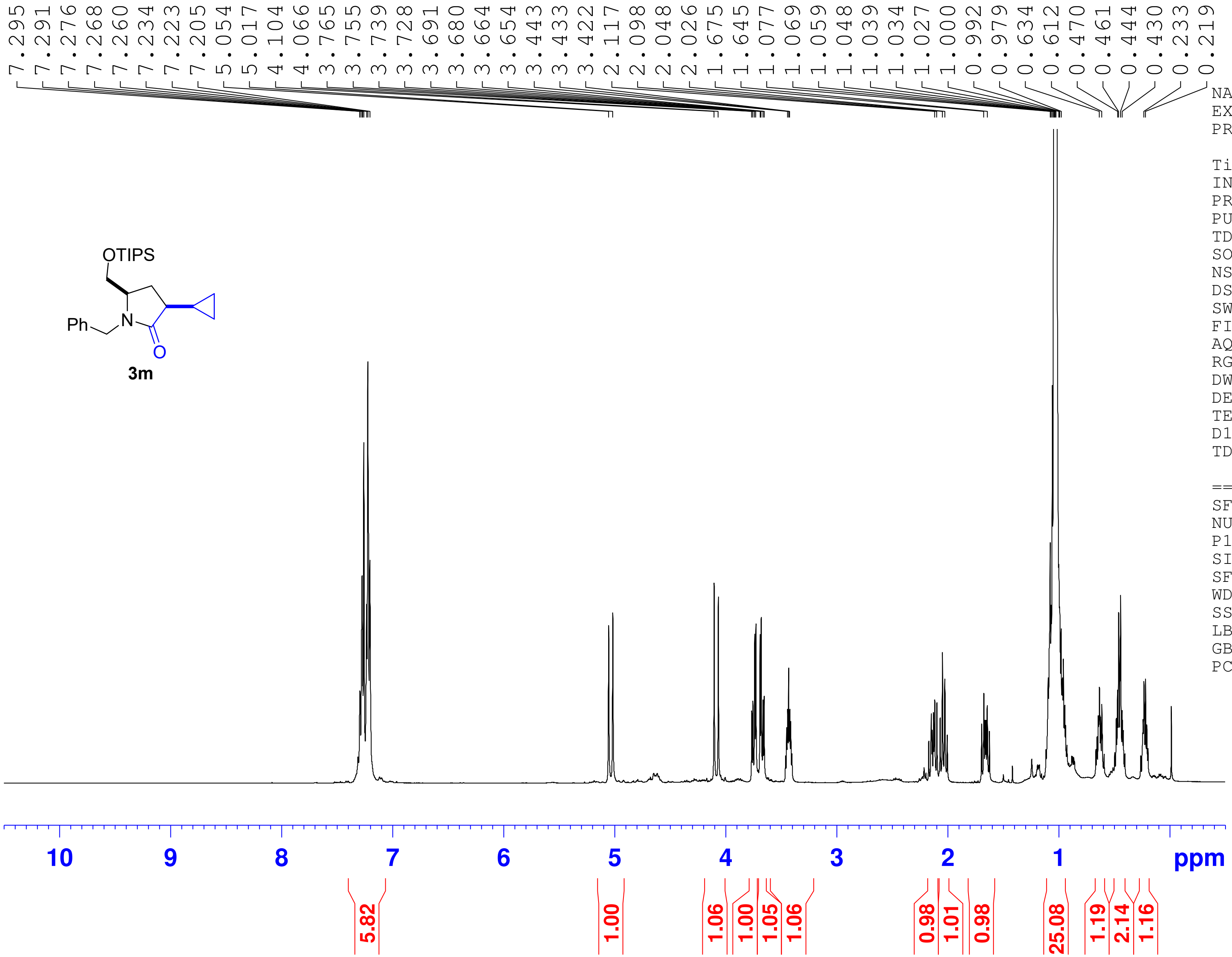

NAME	9-21
EXPNO	1
PROCNO	1
Time	21.26
INSTRUM	spect
PROBHD	5 mm PABBO BB/
PULPROG	zg30
TD	65536
SOLVENT	CDCl3
NS	8
DS	0
SWH	8012.820 Hz
FIDRES	0.122266 Hz
AQ	4.0894966 sec
RG	19.7
DW	62.400 usec
DE	6.50 usec
TE	296.1 K
D1	1.00000000 sec
TD0	1

======== CHANNEL f1 ========

SFO1	400.1324710 MHz
NUC1	1H
P1	14.50 usec
SI	65536
SF	400.1300093 MHz
WDW	EM
SSB	0
LB	0.30 Hz
GB	0
PC	1.00

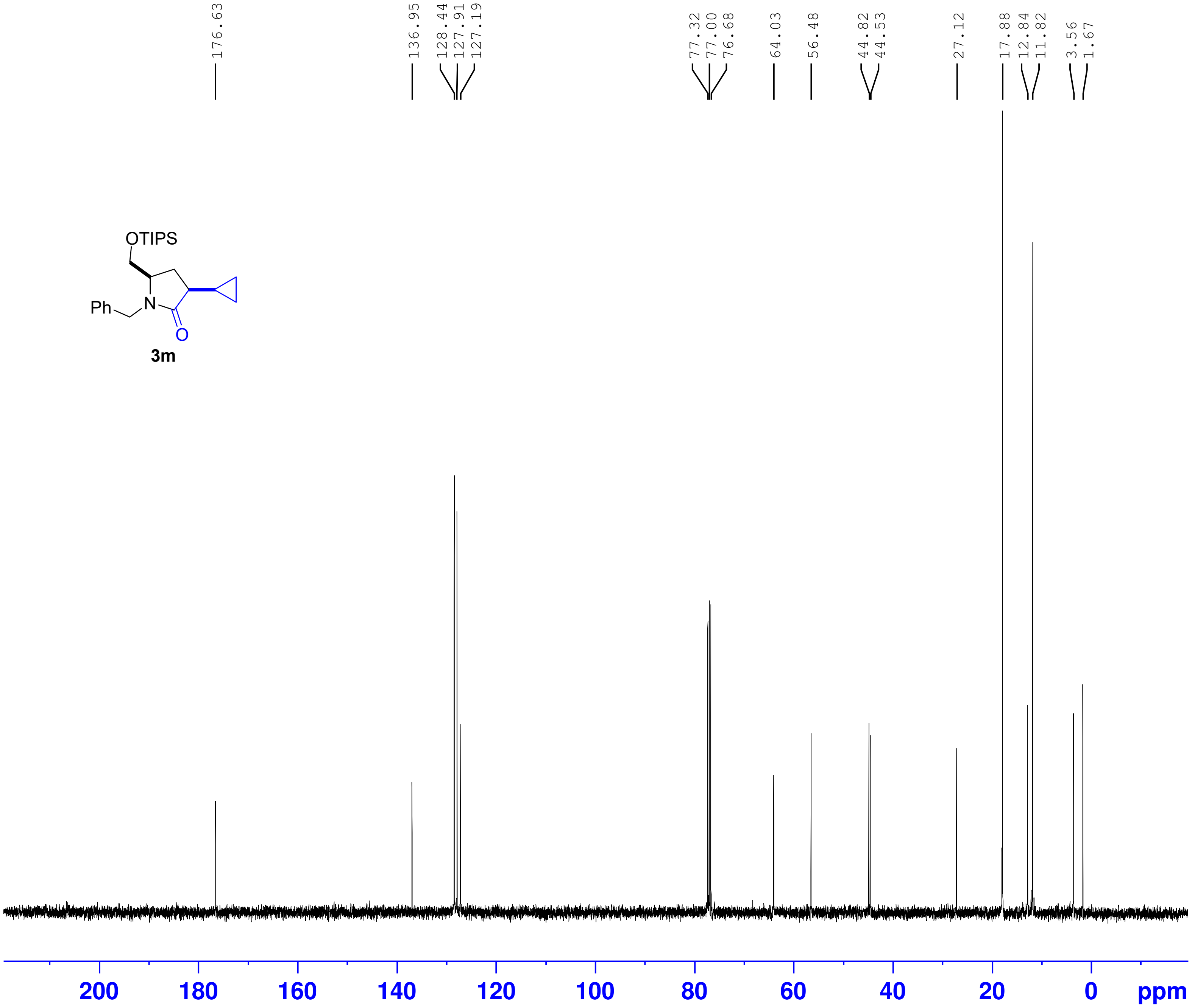

NAME	9-21
EXPNO	2
PROCNO	1

F2 - Acquisition Parameters

Time	11.07
INSTRUM	spect
PROBHD	5 mm PABBO BB/
PULPROG	zgpg30
TD	65536
SOLVENT	CDCl3
NS	32
DS	0
SWH	24038.461 Hz
FIDRES AQ	0.366798 Hz
	1.3631488 sec
RG	196.92
DW	20.800 usec
DE	6.50 usec
TE	296.7 K
D1	2.00000000 sec
D11	0.03000000 sec
TD0	1

======== CHANNEL f1 ========

SFO1	100.6228298 MHz
NUC1	13C
P1	9.70 usec
PLW1	46.98899841 W

======== CHANNEL f2 ========

SFO2	400.1316005 MHz
NUC2	1H
CPDPRG[2	waltz16
PCPD2	90.00 usec
PLW2	11.99499989 W
PLW12	0.34213999 W
PLW13	0.27713001 W

F2 - Processing parameters

SI	32768
SF	100.6127786 MHz
WDW	EM
SSB	0
LB	1.00 Hz
GB	0
PC	1.40

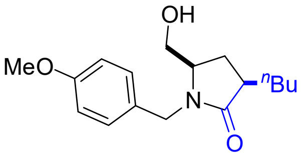

3c'

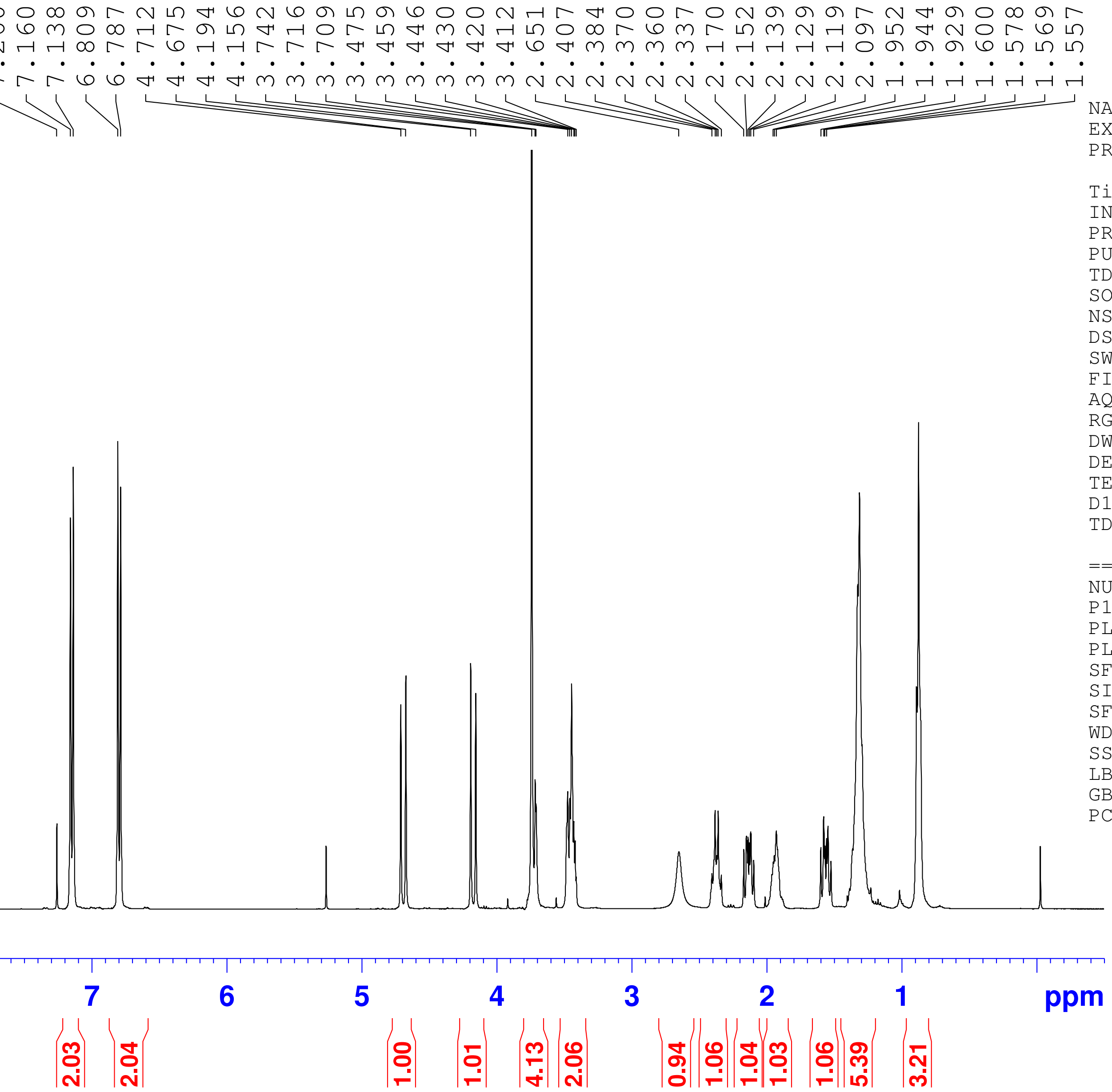

NAME	8-134
EXPNO	1
PROCNO	1
Time	22.09
INSTRUM	spect
PROBHD	5 mm PABBO BB-
PULPROG	zg30
TD	65536
SOLVENT	CDCl3
NS	8
DS	0
SWH	8223.685 Hz
FIDRES	0.125483 Hz
AQ	3.9846387 sec
RG	64
DW	60.800 usec
DE	6.00 usec
TE	295.4 K
D1	1.00000000 sec
TD0	1
======== CHANNEL f1 ========	
NUC1	1H
P1	15.80 usec
PL1	-1.00 dB
PL1W	12.17476940 W
SFO1	400.1324710 MHz
SI	32768
SF	400.1300094 MHz
WDW	EM
SSB	0
LB	0.30 Hz
GB	0
PC	1.00

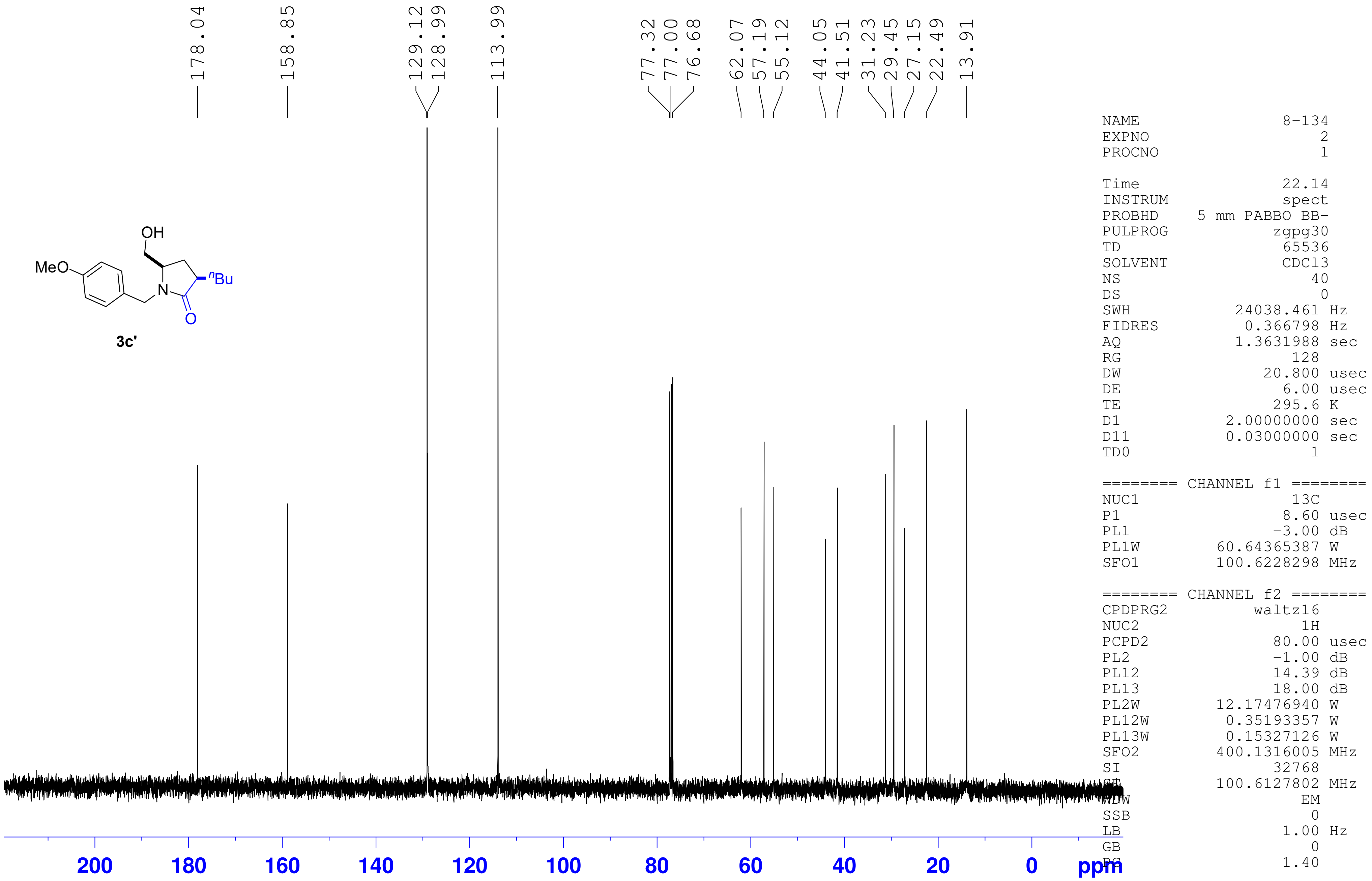
178.04
158.85
129.12
128.99
113.99
77.32
77.00
76.68
62.07
57.19
55.12
44.05
41.51
31.23
29.45
27.15
22.49
13.91
MeO
OH
N
O
nBu
3c'
200
180
160
140
120
100
80
60
40
20
0
ppm
NAME 8-134
EXPNO 2
PROCNO 1
Time 22.14
INSTRUM spect
PROBHD 5 mm PABBO BB-
PULPROG zgpg30
TD 65536
SOLVENT CDCl3
NS 40
DS 0
SWH 24038.461 Hz
FIDRES 0.366798 Hz
AQ 1.3631988 sec
RG 128
DW 20.800 usec
DE 6.00 usec
TE 295.6 K
D1 2.00000000 sec
D11 0.03000000 sec
TD0 1
======== CHANNEL f1 ========
NUC1 13C
P1 8.60 usec
PL1 -3.00 dB
PL1W 60.64365387 W
SFO1 100.6228298 MHz
======== CHANNEL f2 ========
CPDPRG2 waltz16
NUC2 1H
PCPD2 80.00 usec
PL2 -1.00 dB
PL12 14.39 dB
PL13 18.00 dB
PL2W 12.17476940 W
PL12W 0.35193357 W
PL13W 0.15327126 W
SFO2 400.1316005 MHz
SI 32768
SF 100.6127802 MHz
WDW EM
SSB 0
LB 1.00 Hz
GB 0
PC 1.40

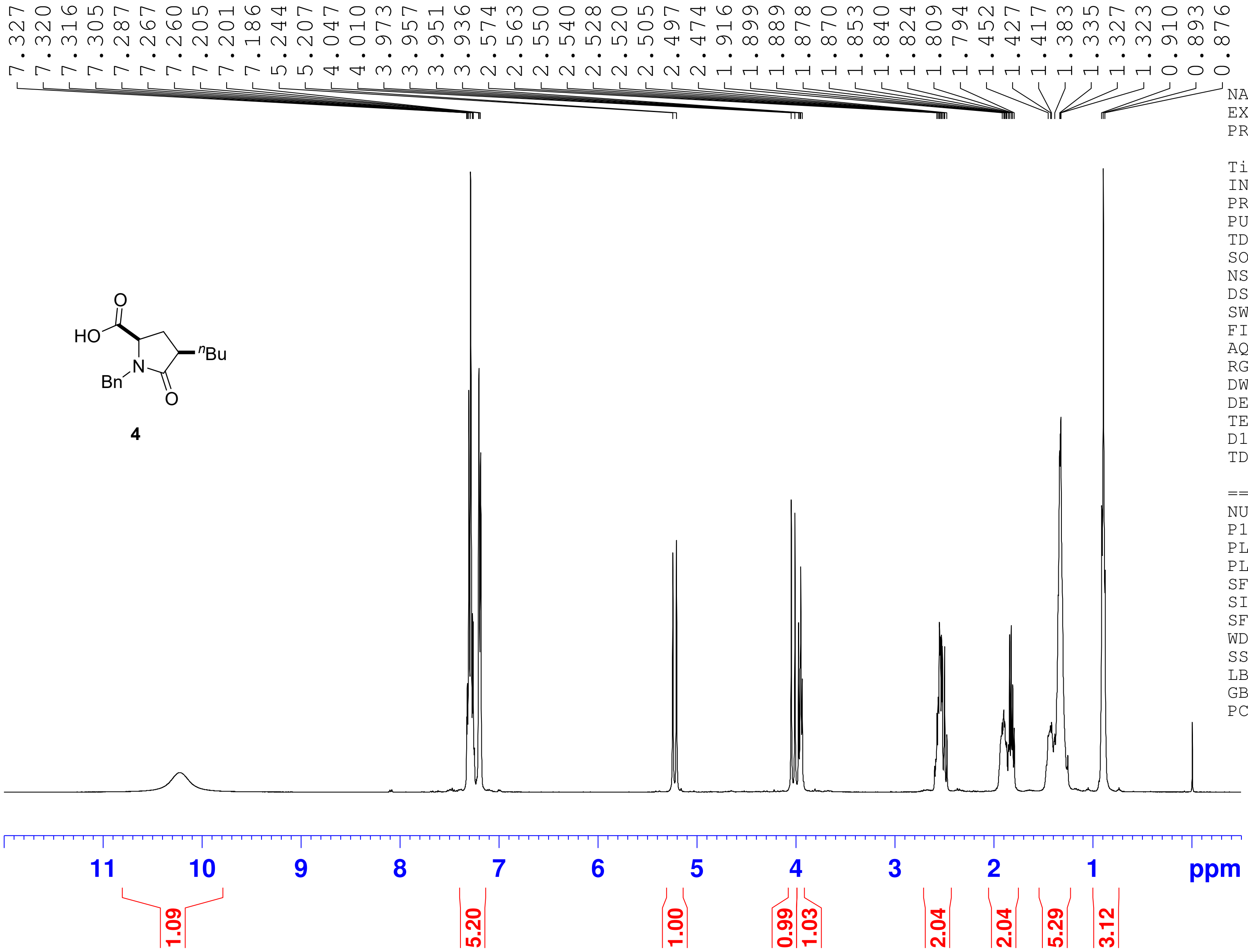

NAME	9-72
EXPNO	1
PROCNO	1
Time	20.37
INSTRUM	spect
PROBHD	5 mm PABBO BB-
PULPROG	zg30
TD	65536
SOLVENT	CDCl3
NS	8
DS	0
SWH	8223.685 Hz
FIDRES	0.125483 Hz
AQ	3.9846387 sec
RG	64
DW	60.800 usec
DE	6.00 usec
TE	294.6 K
D1	1.00000000 sec
TD0	1

======== CHANNEL f1 ========

NUC1	1H
P1	15.80 usec
PL1	-1.00 dB
PL1W	12.17476940 W
SFO1	400.1324710 MHz
SI	32768
SF	400.1300096 MHz
WDW	EM
SSB	0
LB	0.30 Hz
GB	0
PC	1.00

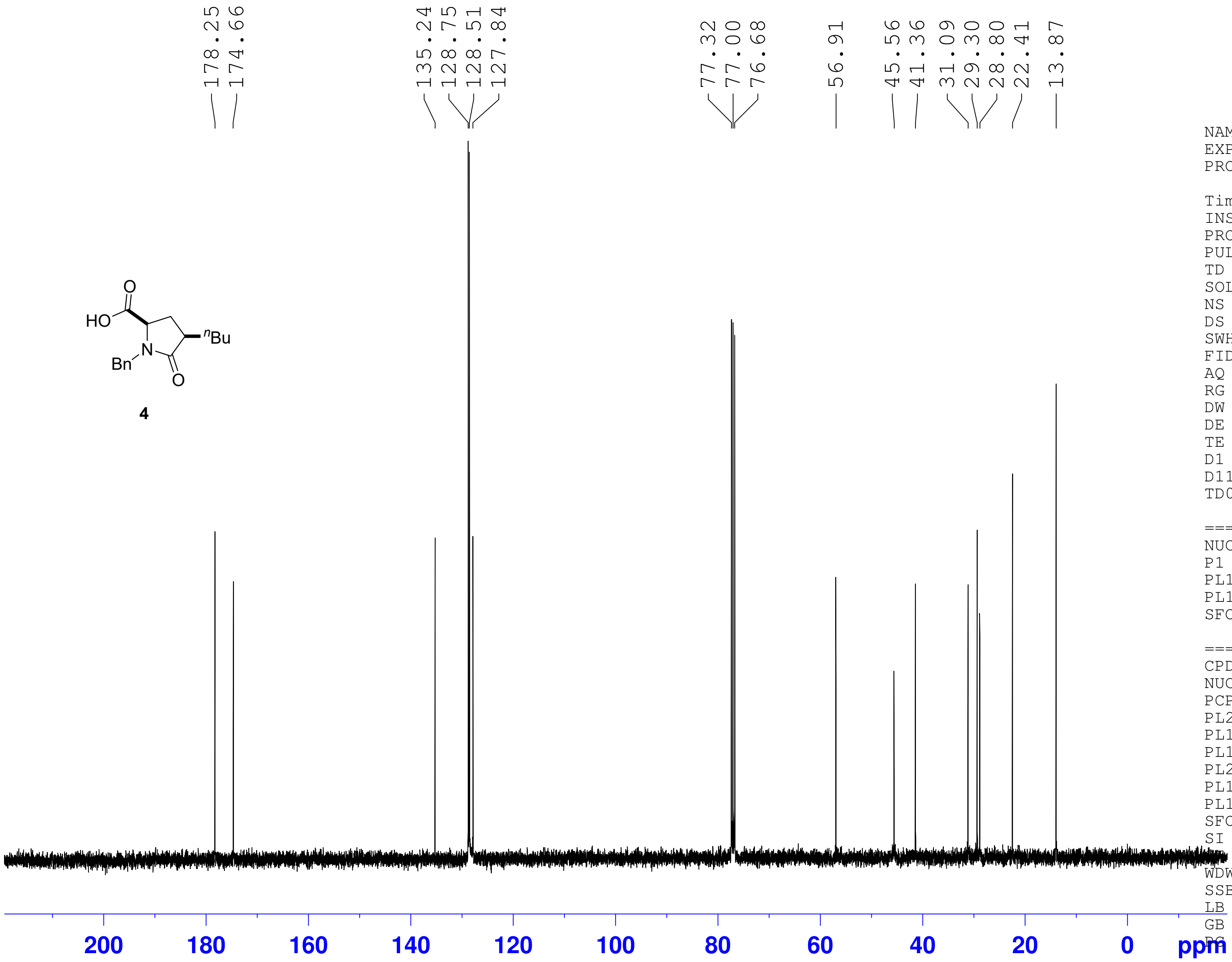

4

NAME	9-72
EXPNO	2
PROCNO	1
Time	20.39
INSTRUM	spect
PROBHD	5 mm PABBO BB-
PULPROG	zgpg30
TD	65536
SOLVENT	CDCl3
NS	80
DS	0
SWH	24038.461 Hz
FIDRES	0.366798 Hz
AQ	1.3631988 sec
RG	322
DW	20.800 usec
DE	6.00 usec
TE	294.9 K
D1	2.00000000 sec
D11	0.03000000 sec
TD0	1
======== CHANNEL f1 ========	
NUC1	13C
P1	8.60 usec
PL1	-3.00 dB
PL1W	60.64365387 W
SFO1	100.6228298 MHz
======== CHANNEL f2 ========	
CPDPRG2	waltz16
NUC2	1H
PCPD2	80.00 usec
PL2	-1.00 dB
PL12	14.39 dB
PL13	18.00 dB
PL2W	12.17476940 W
PL12W	0.35193357 W
PL13W	0.15327126 W
SFO2	400.1316005 MHz
SI	32768
SF	100.6127780 MHz
WDW	EM
SSB	0
LB	1.00 Hz
GB	0
PC	1.40

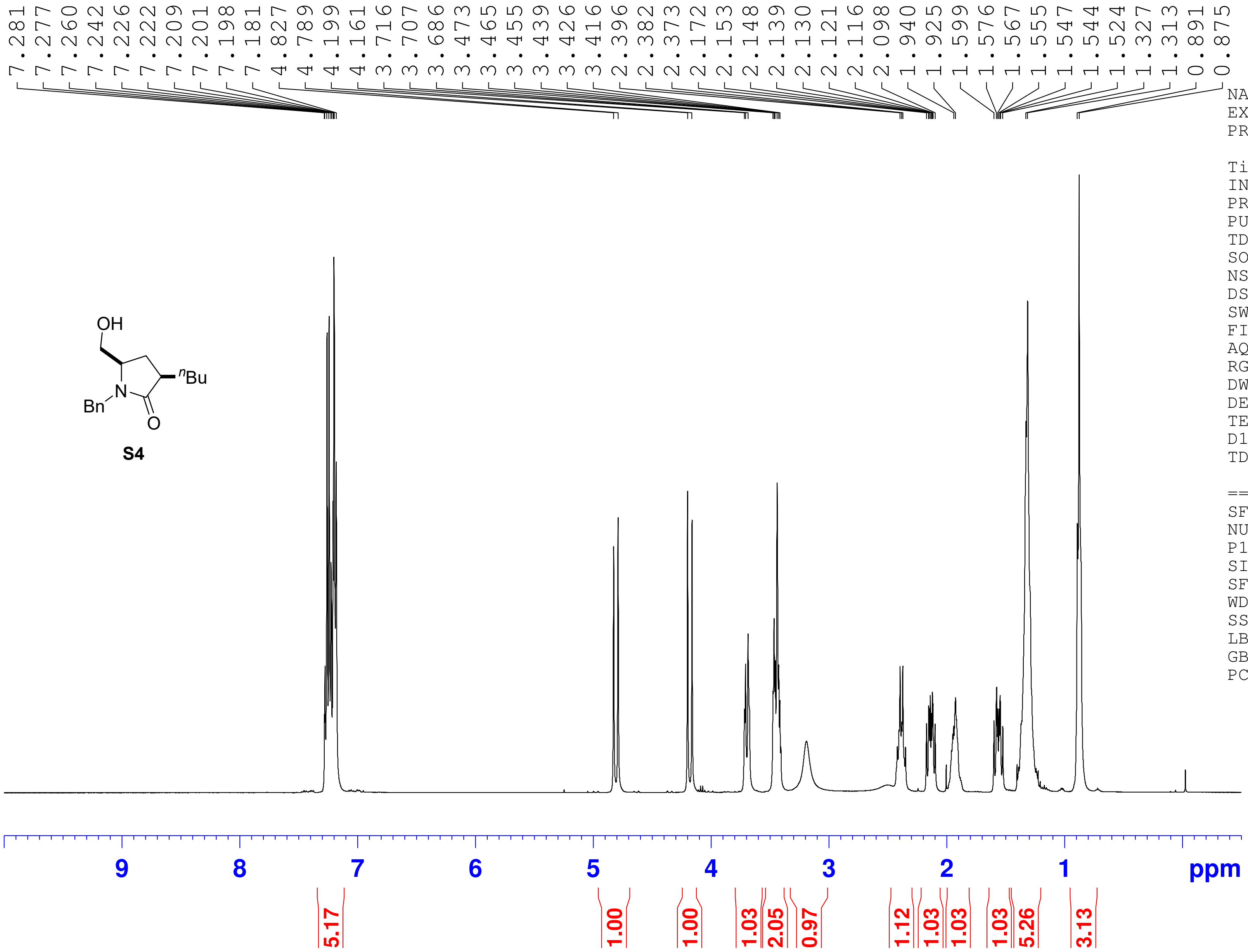

S4

NAME	8-125
EXPNO	1
PROCNO	1
Time	21.13
INSTRUM	spect
PROBHD	5 mm PABBO BB/
PULPROG	zg30
TD	65536
SOLVENT	CDCl3
NS	8
DS	0
SWH	8012.820 Hz
FIDRES	0.122266 Hz
AQ	4.0894966 sec
RG	17.38
DW	62.400 usec
DE	6.50 usec
TE	297.1 K
D1	1.00000000 sec
TD0	1

======== CHANNEL f1 ========

SFO1	400.1324710 MHz
NUC1	1H
P1	14.50 usec
SI	65536
SF	400.1300100 MHz
WDW	EM
SSB	0
LB	0.30 Hz
GB	0
PC	1.00

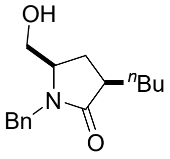

S4

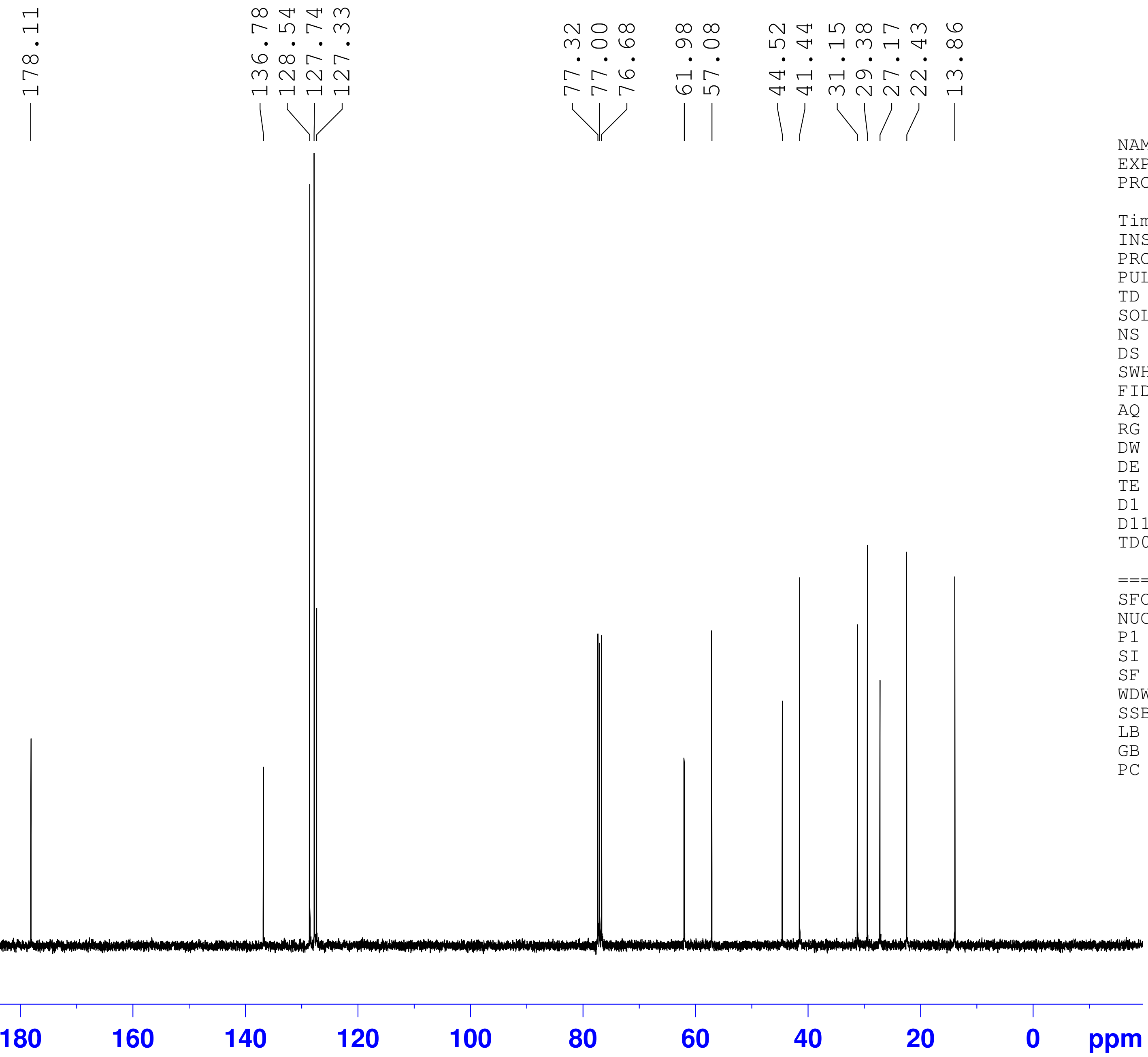

NAME 8-125
EXPNO 2
PROCNO 1

Time 21.15
INSTRUM spect
PROBHD 5 mm PABBO BB/
PULPROG zgpg30
TD 65536
SOLVENT CDCl3
NS 40
DS 0
SWH 24038.461 Hz
FIDRES 0.366798 Hz
AQ 1.3631988 sec
RG 196.92
DW 20.800 usec
DE 6.50 usec
TE 297.8 K
D1 2.00000000 sec
D11 0.03000000 sec
TD0 1

======== CHANNEL f1 ========
SFO1 100.6228298 MHz
NUC1 13C
P1 9.70 usec
SI 32768
SF 100.6127858 MHz
WDW EM
SSB 0
LB 1.00 Hz
GB 0
PC 1.40
